# An Unexpected and Significantly Lower Hydrogen-Bond-Donating Capacity of Fluorohydrins Compared to Nonfluorinated Alcohols**

**DOI:** 10.1002/anie.201202059

**Published:** 2012-05-10

**Authors:** Jérôme Graton, Zhong Wang, Anne-Marie Brossard, Daniela Gonçalves Monteiro, Jean-Yves Le Questel, Bruno Linclau

**Affiliations:** Chemistry, University of SouthamptonHighfield, Southampton SO17 1BJ (UK); CEISAM UMR CNRS 6230, Faculté des Sciences et des Techniques, Université de Nantes2, rue de la Houssinière – BP 92208, 44322 NANTES Cedex 3 (France)

**Keywords:** computational chemistry, conformation analysis, fluorine, fluorohydrins, hydrogen bonding

The success of fluorination in improving molecular properties over a wide range of applications (including pharmaceuticals,^1^ agrochemicals,^2^ materials,^3^ and crystal engineering^4^) has been remarkable. Up to 20 % of the pharmaceuticals prescribed or administered in the clinic, and a third of the leading 30 blockbuster drugs, contain at least one fluorine atom1a and 30–40 % of currently marketed agrochemicals contain fluorine.^5^

In many cases, fluorine is introduced following a particular rationale.^6^ Examples include enhancement of metabolic stability, functional-group (FG) reactivity or acid/base-property modification, and conformational stabilization. Importantly, these alterations cannot be considered individually as usually a number of properties are influenced simultaneously.^7^ For example, fluorination of amines in order to decrease their p*K*_a_ value also leads to an increase in their lipophilicity and may induce significant conformational changes. Furthermore, this decrease in p*K*_a_ can be attenuated if intramolecular NH^+^⋅⋅⋅F electrostatic interactions can occur.^8^ Hence, a comprehensive understanding of the effects of fluorination is a prerequisite for successful planning and rationalization of fluorine introduction, and research that increases our knowledge in that respect is highly relevant.

The hydrogen bond (H-bond) is an important specific interaction between a molecule and its local environment.^9^ Crucial functional roles include the binding of ligands to protein receptors and the promotion of enzyme catalysis. In the design of bioactive compounds, H-bonding impacts on a wide range of molecular properties such as potency, selectivity, permeability, and solubility.^10^ Given the strong electrostatic contribution to the overall energy of an H-bond,^11^ introduction of the small and highly electronegative fluorine atom is expected to significantly modify the H-bond properties of an adjacent FG. It is therefore surprising that despite H-bond acidity of alcohols has been previously studied,^12^ a thorough investigation of the influence of fluorination on H-bond acidity appears limited to that of polyfluorinated solvents such as trifluoroethanol (TFE) and hexafluoroisopropyl alcohol (HFIP),^13^ and to certain supramolecular receptor systems.^14^ TFE and HFIP are very strong H-bond donors (and very poor acceptors), which has been exploited, when they were used as solvents, to influence the reactivity of certain reagents.13a, 15 The H-bond properties of TFE and HFIP are generally considered to originate from the strong inductive effect of fluorine, leading to statements in the literature such as “the ability of fluorine …… as an inductive activator of a H-bond donor group”^16^ and “fluorination always increases H-bond acidity”.^17^

Herein, we show that this is incorrect as a general rule. Indeed, experimental determination of H-bond acidities of a range of fluorohydrins shows that fluorination can lead to an attenuation, in some cases very pronounced, of H-bond acidity. In order to exclude conformational complications (e.g., the fluorohydrin *gauche* effect),^18^, ^19^ this study was carried out using conformationally restricted model compounds **1**–**8** (Scheme 1), which adopt only chair conformations as confirmed by computational analysis (see below). The obtained values have been compared to the H-bond acidities of the corresponding nonfluorinated alcohols **9** and **10**.

**Scheme 1:**
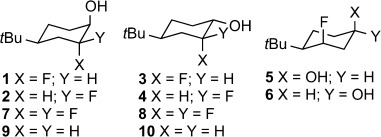
Fluorohydrin model compounds and nonfluorinated reference alcohols.

The synthesis of fluorohydrins **1**–**8** is detailed in the Supporting Information. Of note is the diastereoselectivity observed in the reductions of 2-fluoroketones **11** and **12** (Scheme 2). The reduction of **11** with L-selectride gave only equatorial attack, as observed with (nonfluorinated) 4-*tert*-butylcyclohexanone,^20^ but a complete reversal in diastereoselectivity was found for the reduction of **12** using the same reagent. This is the first report of a fully diastereoselective reduction of each of the diastereomeric 2-fluorocyclohexanones.18c, 21

**Scheme 2:**
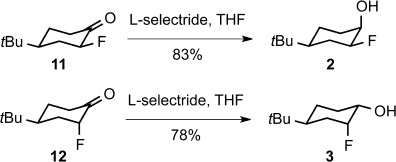
Stereoselective reduction of 2-fluorocyclohexanone diastereomers.

The relative H-bond acidities p*K*_AHY_ of **1**–**10** (Table 1, Figure 1) were determined by adapting an established procedure using FTIR spectroscopy.^22^ The decrease in absorbance of the ν_OH_ stretching band of the hydroxy group upon complexation with *N*-methylpyrrolidinone (NMP) was monitored in dilute CCl_4_ solution.

**Figure 1:**
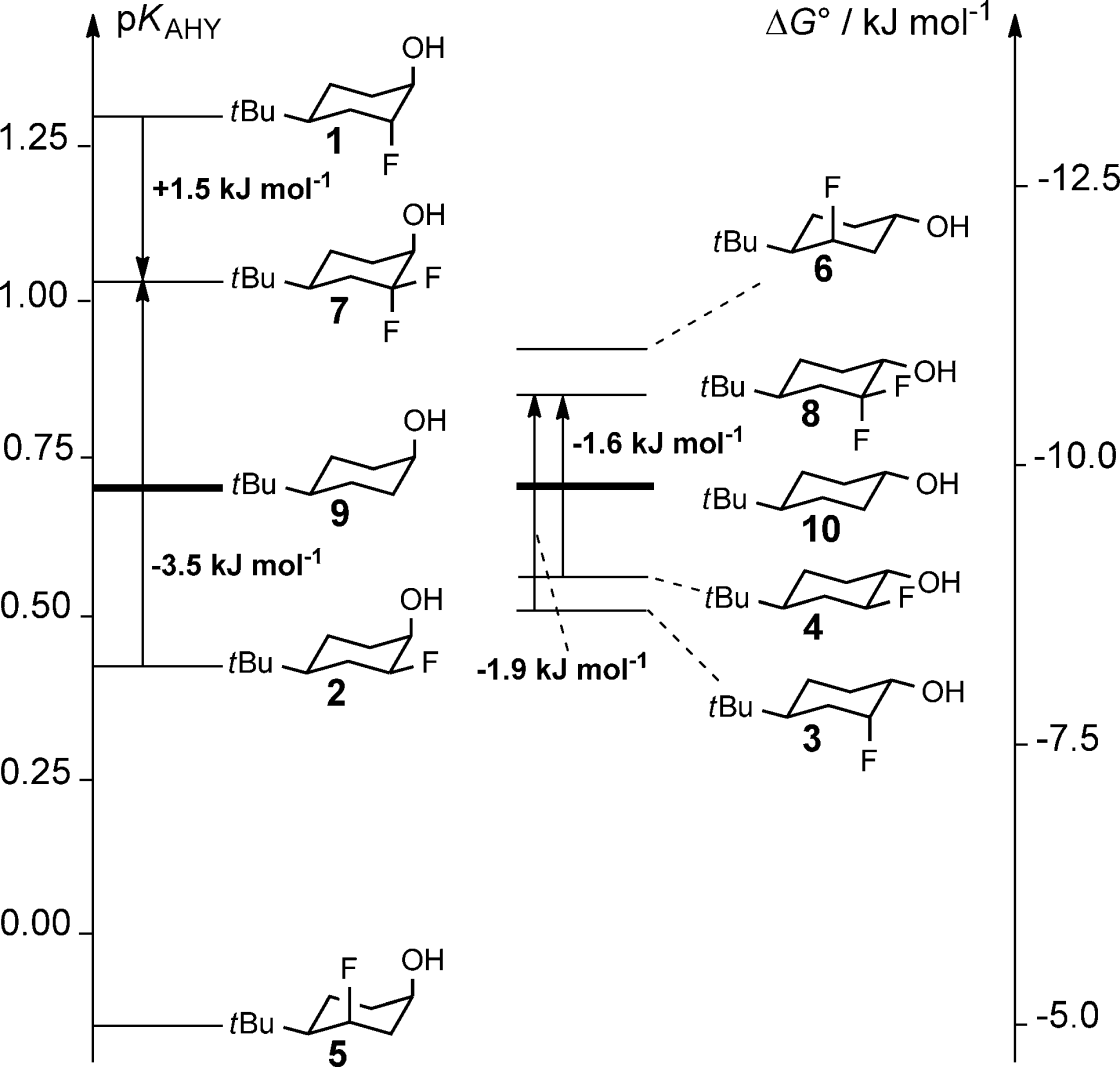
Visualization of the H-bond acidity range of **1**–**10**, with selected energetic differences between mono- and difluorinated alcohols.

**Table 1:** Determination of fluorohydrin H-bond acidity in CCl_4_ at 25 °C.
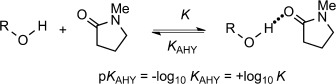

Compound	*K*^a^	p*K*_AHY_	Δ*G*° b	*δ*Δ*G*° b,c
**9**	5.10	0.71	−9.8	–
**10**	5.06	0.70	−9.8	–
**1**	19.94	1.30	−13.2	−3.4
**2**	2.70	0.43	−8.2	+1.6
**3**	3.26	0.51	−8.7	+1.1
**4**	3.63	0.56	−9.0	+0.8
**5**	0.71	−0.15	−4.9	+4.9
**6**	8.45	0.93	−11.1	−1.3
**7**	10.8	1.03	−11.7	−1.9
**8**	7.00	0.85	−10.6	−0.8

a In dm^3^ mol^−1^.

b Δ*G°* [kJ mol^−1^]=−5.708 p*K*_AHY_−5.781.

c Defined as Δ*G*°(fluorohydrin)−Δ*G*°(corresponding nonfluorinated alcohol).

The equilibrium constant *K* was first determined for the reference compounds, *cis*- and *trans-*4-*tert*-butylcyclohexanol, **9** and **10**, respectively. Interestingly, there was no difference between these two alcohols (axial and equatorial), which to the best of our knowledge has not previously been established.^23^ Of the monosubstituted 1,2-fluorohydrins, only *trans* diaxial **1** showed the expected increase in H-bond acidity, with an almost four-fold higher *K* value. In contrast, fluorohydrins **2**–**4** showed a decreased H-bond acidity, with equilibrium constants amounting to 53–72 % of the value of the nonfluorinated alcohols. For 1,3-fluorohydrins, the *cis* isomer **5** had a dramatically decreased H-bond acidity, with the hydroxy group virtually losing its ability to act as a H-bond donor. In contrast, the *trans* isomer **6** shows a significant increase of the H-bond donating capacity. Both 2,2-difluoroalcohols, **7** and **8**, have a greater H-bond acidity than the nonfluorinated alcohols, with a stronger enhancement for the axial hydroxy group in **7**.

These results demonstrate the importance of the relative fluorohydrin configuration on H-bond acidity, and that the fluorine inductive effect cannot be the only factor determining the magnitude of the acidity. Apart from the reduced H-bond acidities of **2**–**4** compared to **9** and **10**, the most surprising observations are the reduced H-bond acidity of the difluorohydrins compared to certain monofluorohydrins (e.g., **7** and **1**, and even **8** and **6**, which is though a 1,3-fluorohydrin), and the dramatically reduced H-bond acidity of **5** compared to **9**.

Quantum chemical calculations strongly support these experimental trends. Conformation analysis (MPWB1K/6-31+G(d,p) level in vacuum) of all cyclohexanols, which only involves rotation around the C–O bond (Table 2), shows that there is only one predominant conformer (two in **8**) for those structures in which the hydroxy proton can be located close to the fluorine atom. When the modeling of bulk-solvent effects is included (in this case CCl_4_), through the polarizable continuum model (PCM),^24^ the relative population of the solvated conformers is distributed similarly to that of the isolated structures (see the Supporting Information). For **2**–**4**, **7**, and **8**, the energy barrier Δ*G*_TS_ to rotation of the O–H bond is calculated to be in the range of 11–13 kJ mol^−1^, whereas it is only 3–5 kJ mol^−1^ for **1**, **6**, **9**, and **10**. With the 1,3-coaxial fluorohydrin **5**, this energy barrier is raised to 18 kJ mol^−1^.^25^ This significant variation is attributed to the occurrence of an F⋅⋅⋅HO interaction, which is particularly favorable in the case of a 1,3-coaxial fluorohydrin, as illustrated by NMR spectroscopy (^h1^*J*_F-HO_≍12 Hz),^26^ and by the significantly shorter calculated *d*_F⋅⋅⋅H_ distance in **5** (2.033 Å) than found in the 1,2-fluorohydrins (2.3–2.4 Å).

**Table 2:** Relative Gibbs energies (Δ*G*) and populations (*p_i_* ) of 1–10, with characteristics of the O–H bond.
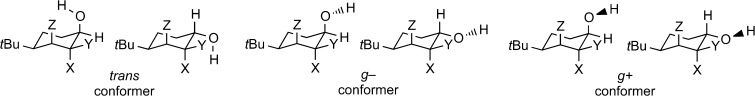

Entry	*trans*		*g−*		*g+*		Δ*G*_TS_^a^,^c^	*E*^(2)^_n→σ^*^_^a^,^d^	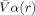 e,f	*d*_H⋅⋅⋅F_^g^
	Δ*G*^a^	*p_i_*^b^	Δ*G*^a^	*p_i_*^b^	Δ*G*^a^	*p_i_*^b^				
**9**	1.0	24.9	0.0	75.1^h^	h	h	4.0	–	0.3184	–
**10**	0.8	26.8	0.0	73.2^h^	h	h	4.6	–	0.3177	–
**1**	3.6	12.5	0.0	54.4	1.2	33.0	3.1	–	0.3315	–
**2**	14.9	0.2	10.7	1.3	0.0	**98.4**	12.2	4.1	0.3127	2.287 (*g*+)
**3**	0.0	**97.5**	11.7	0.9	10.1	1.6	10.9	4.1	0.3108	2.291 (*trans)*
**4**	12.2	0.7	10.8	1.2	0.0	**98.1**	11.7	3	0.3133	2.372 (*g*+)
**5**	0.0	**99.7**	14.7	0.3	i	i	17.9	17.1	0.2915	2.033 (*trans)*
**6**							3.6	–	0.3227	–
**7**	14.8	0.2	9.7	2.0	0.0	**97.8**	11.3	2.2	0.3262	2.332 (*g*+)
**8**	0.6	**43.4**	11.9	0.5	0.0	**56.1**	12.9	4.2	0.3193	2.275 (*trans)* 2.364 (*g*+)

a In kJ mol^−1^.

b Boltzmann population in %.

c Barrier height for O–H rotation.

d Interaction energy from F lone pair to σ^*^_O–H_ charge transfer.

e Kenny electrostatic potential, weighted by the conformer’s Boltzmann population.

h In a.u.

g F⋅⋅⋅HO distance, in Å.

h Degenerated conformation.

i No local minima found.

The topic of fluorine-mediated H-bonding has been subject to extensive debates,9c, 27 but the stabilizing nature of 1,3-*syn* F⋅⋅⋅HX interactions has been invoked to explain conformational effects in fluorinated amines and amides,^7^ as well as p*K*_a_ modulations in the former.^7^, ^8^ Interestingly, NBO (natural bond orbital) and AIM (atoms in molecules) analyses reveal, for fluorohydrin **5**, a significant charge transfer (17.1 kJ mol^−1^) from the fluorine lone pair to the antibonding σ*_OH_ orbital and a bond critical point (BCP) between the F and H atoms (*ρ*=0.0192 *e*), a weak value typical of an H-bond interaction.^28^ Much weaker charge transfer (between 2.2 and 4.2 kJ mol^−1^, Table 2) and no BCP are found for the vicinal fluorohydrins, suggesting that in these cases, this intramolecular OH⋅⋅⋅F interaction probably represents more a weak electrostatic stabilization than a typical H-bond.^29^ Interestingly, no BCP was found between the F and H atoms in 2-fluorophenol,^30^ and in glucopyranose, AIM analysis detected intramolecular H-bonding between 1,3-diol groups, but not for vicinal diols.^31^

Because of the electrostatic character of H-bond interactions, various theoretical descriptors based on the electrostatic potential have been extensively used.^32^ The *V*_α_(*r*) descriptor,^33^ which is calculated at a distance of 0.55 Å from the hydrogen atom along the O–H bond, was calculated (in vacuum) for all fluorohydrin conformers, and weighted by their relative populations. Pleasingly, an excellent correlation was found between *V*_α_(*r*) and the experimentally determined H-bond acidity (*r*^2^=0.978, s=0.06, Figure 2). Hence, this descriptor accurately predicts the H-bond acidity for alcohols that are predominantly in a conformation with F⋅⋅⋅HO contact.

**Figure 2:**
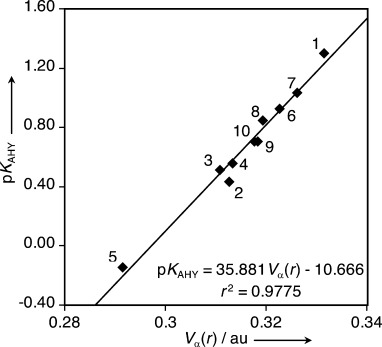
Correlation between the H-bond acidity of **1**–**10** and the Kenny electrostatic potential descriptor *V*_α_(*r*).

The above analysis allows rationalization of the experimental trends: whereas the fluorine inductive effect is responsible for the four-fold increase in H-bond acidity of **1** (compared to **9**), any decrease in H-bond acidity is attributed to the unavailability of the OH group owing to an intramolecular F⋅⋅⋅HO interaction. It also explains (Figure 1) why the introduction of a second fluorine atom into **1** (to give **7**) leads to a decrease in H-bond acidity (this new fluorine atom can engage in an intramolecular F⋅⋅⋅HO interaction), and why the introduction of an *anti* fluorine substituent into **2** (to also give **7**) leads to a strong H-bond acidity increase. The reduction of H-bond acidity due to intramolecular H-bonding had been reported previously for a series of phenols, and the energy of the intramolecular H-bond was shown to be unrelated to the overall H-bond acidity of the studied structures.^34^

Another important conclusion is that the electronegativity of a *gauche* fluorine substituent is only partially translated to the hydroxy group: for compounds **2**–**4** (all containing F⋅⋅⋅HO), the increase in H-bond acidity resulting from introduction of an *anti* fluorine substituent is about twice the increase in H-bond acidity resulting from introduction of a *gauche* fluorine substituent (Figure 1: compare **2**→**7** with **3**,**4**→**8**). These observations are corroborated by Bols et al., in the context of amine basicity and glycoside hydrolysis, who concluded that the electronegativity of a polar substituent (e.g., OH, F) is greater in an antiperiplanar arrangement, compared to a *gauche*,^8^, ^35^ which may originate from a stereochemical dependence of hyperconjugation donor/acceptor abilities of σ bonds involved.^36^

Given the weak nature of an F⋅⋅⋅HO interaction, its ability to overturn the influence of the fluorine electronegativity is most surprising, even if the incomplete translation of electronegativity due to a *gauche* dihedral angle is taken into account. Consideration of the *V*_α_(*r*) values of the unchelated conformers of **2**–**5**, **7**, and **8** allows estimation of a putative H-bond acidity, the difference with the experimental value then being an estimate of the loss in H-bond acidity caused by the F⋅⋅⋅HO interaction. This amounts to around 2–4 kJ mol^−1^ for the 2-, and 6 kJ mol^−1^ for the 3-fluorocyclohexanols (see the Supporting Information). In this context, it is interesting to observe that the F⋅⋅⋅HN^+^ interaction is *not* overriding the effect of fluorine electronegativity on the p*K*_a_ of ammonium ions, but only attenuates the decrease in p*K*_a_.^8^

In summary, we reported alcohol H-bond acidity measurements of a range of conformationally restricted fluorohydrins. The results force the conclusion that contrary to current assumptions, fluorination can lead to a significant attenuation of alcohol H-bond acidity compared to the corresponding nonfluorinated alcohols. DFT calculations indicate that intramolecular F⋅⋅⋅HO interactions are responsible for the H-bond acidity attenuation, and it is most remarkable that these weak interactions outcompete the fluorine electron-withdrawing effect. However, the obtained data also indicate that the effect of fluorine electronegativity strongly depends on the fluorohydrin dihedral angle, with a 180° dihedral angle required for maximum effect, and with an estimated halving of the fluorine electron-withdrawing power for a 60° (*gauche*) dihedral angle. These insights open up new opportunities for compound-property modification through fluorination in a wide range of applications where H-bonding is important. Research on a wider range of substrates is currently ongoing in our groups.

## Supplementary material

Detailed facts of importance to specialist readers are published as ”Supporting Information”. Such documents are peer-reviewed, but not copy-edited or typeset. They are made available as submitted by the authors.
