# Supplementary material for: An Unexpected and Significantly Lower Hydrogen-Bond-Donating Capacity of Fluorohydrins Compared to Nonfluorinated Alcohols
Source: Angew Chem Int Ed Engl. 2012 May 10;51(25):6176–80. doi: 10.1002/anie.201202059 (PMC3601419; doi:10.1002/anie.201202059)

Angewandte Chemie
Eine Zeitschrift der Gesellschaft Deutscher Chemiker

Supporting Information

© Wiley-VCH 2012

69451 Weinheim, Germany

# An Unexpected and Significantly Lower Hydrogen-Bond-Donating Capacity of Fluorohydrins Compared to Nonfluorinated Alcohols**

*Jérôme Graton,* Zhong Wang, Anne-Marie Brossard, Daniela Gonçalves Monteiro, Jean-Yves Le Questel, and Bruno Linclau**

anie_201202059_sm_miscellaneous_information.pdf

**Table of contents**


1. Synthesis of the fluoroalcohols 2

1.1 Stereoselective synthesis of fluoroalcohols **2** and **3**: reduction of fluoroketones **11** and **12** by L-Selectride (see Scheme 2) 2

1.2 NMR spectra of the crude product obtained from reduction of **11**. 4

1.3 NMR spectra of the crude product obtained from reduction of **12**. 5

1.4 Synthesis of fluorohydrins **1** and **4**. 6

1.5 Synthesis of the 1,3-fluorohydrins **5** and **6** 8

1.5.1 Synthesis of **6** 8

1.5.2 Synthesis of **5** 11

1.6 Synthesis of the 2,2-difluoroalcohols **7** and **8** 13

2. Hydrogen bond acidity measurements 15

3. Quantum chemistry calculations 16

3.1 Conformational study 16

3.2 Hydrogen bond acidity characterisation 17

4. Copies of $^{1}H$, $^{13}C$, and $^{19}F$ spectra 19

4.1 Fluorohydrin **1** 19

4.2 Fluorohydrin **2** 21

4.3 Fluorohydrin **3** 23

4.4 Fluorohydrin **4** 25

4.5 Fluorohydrin **5** 27

4.6 Fluorohydrin **6** 29

4.7 Difluorohydrin **7** 31

4.8 Difluorohydrin **8** 33

4.9 Allylic alcohol **SI1** 35

4.10 Iodofluorination product **SI2** 36

4.11 Mitsunobu product **SI3** 38

## 1. Synthesis of the fluoroalcohols

### 1.1 Stereoselective synthesis of fluoroalcohols 2 and 3: reduction of fluoroketones 11 and 12 by L-Selectride (see Scheme 2):

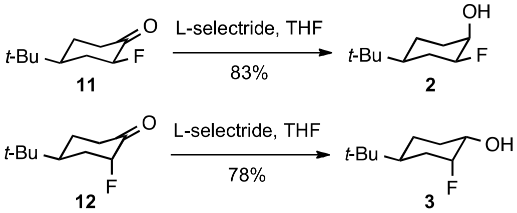


Compound **2**: To a solution of L-Selectride (4.36 ml, 1 M in THF, 4.36 mmol, 1.5 equiv.) in dry THF (4.4 mL) was added a solution of fluoroketone **11**[1] (500 mg, 2.91 mmol, 1 equiv.) in THF (2.3 mL) at -78 °C. The temperature was maintained for 2 h, then stirred at room temperature overnight. On the following day, the reaction mixture was cooled to 0 °C and quenched with water (20 mL). After warmed to room temperature, NaOH (2 mL, 3 M) and $H_2O_2$ (2 mL, 30%) was added and stirred for another 2 h. The mixture was then extracted with diethyl ether (3 × 30 mL). The combined organic layer was washed with brine (40 mL) and water (40 mL), dried over $MgSO_4$, filtered, evaporated. The crude was purified by flash chromatography to afford **2,** 421 mg (colourless oil, 2.41 mmol, 83%).

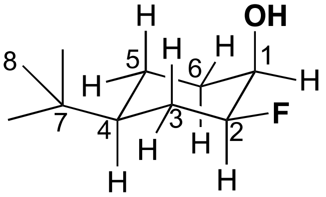


**IR** 3598(br,w), 3429(br,w), 2956(s), 2868(m), 1483(w), 1381(m), 1363(m), 1248(m), 1128(m), 1040(m), 998(s) $cm^{-1}$; **$^{1}$H NMR** (400 MHz, $CDCl_3$) δ 4.50 (1H, dddd, *J* 47.1, 11.6, 4.8, 2.8 Hz, H2), 4.10–4.20 (1H, m, H1), 1.97–2.08 (2H, m, H6-eq + OH), 1.82–1.91 (1H, m, H3-eq), 1.64 (1H, ddd, *J* 24.0, 11.9, 8.7 Hz, H3-ax), 1.29–1.49 (3H, m, H5-eq + H6-ax + H5-ax), 1.02–1.11 (1H, m, H4), 0.89 (9H, s, H8) ppm; **$^{1}$H{$^{19}$F} NMR** (400 MHz, $CDCl_3$) δ 4.46–4.54 (1H, m, a coupling of 11.5 Hz can be observed, H2), 4.15 (1H, br. s., H1), 1.95–2.06 (2H, m, H6-eq + OH), 1.87 (1H, br. d, *J* 11.4 Hz, H3-eq), 1.64 (1H, dd, *J* 24.0, 12.1 Hz, H3-ax), 1.29–1.49 (3H, m, H5-eq + H6-ax + H5-ax), 1.02–1.11 (1H, m, H4), 0.90 (9H, s, H8) ppm; **$^{13}$C NMR** (75 MHz, $CDCl_3$) δ 94.7 (d, *J* 174.7 Hz, C2), 66.9 (d, *J* 17.7 Hz, C1), 46.0 (d, *J* 10.0 Hz, C4), 32.3 (C7), 29.6 (d, *J* 6.6 Hz, C6), 27.5 (C8), 26.9 (d, *J* 16.6 Hz, C3), 19.4 (C5) ppm; **$^{19}$F NMR** (282 MHz, $CDCl_3$) δ -179.8 (d, *J* 46.2 Hz) ppm; **MS**(ESI-) *m/z* 173.0 $[M-H]^-$.

[1] S. E. Denmark, Z. Wu, C. M. Crudden, H. Matsuhashi, *J. Org. Chem.* **1997**, *62*, 8288–8289.

The $^{19}$F NMR chemical shift, and $J_{C1\text{-}3\text{–}F}$ values correspond to the literature.[2]

Compound **3**: To a solution of L-Selectride (35.7 ml, 1 M in THF, 35.7 mmol, 1.5 equiv.) in dry THF (35 mL) was added a solution of fluoroketone **12**[1] (4.1 g, 23.8 mmol, 1 equiv.) in THF (20 mL) at -78 °C. The temperature was maintained for 2 h, then stirred at room temperature overnight. On the following day, the reaction mixture was cooled to 0 °C and quenched with water (80 mL). After warmed to room temperature, NaOH (20 mL, 3 M) and $H_2O_2$ (20 mL, 30%) was added and stirred for another 2 h. The mixture was then extracted with diethyl ether (3 × 150 mL). The combined organic layer was washed with brine (150 mL) and water (150 mL), dried over $MgSO_4$, filtered, evaporated. The crude was purified by flash chromatography to afford **3**, 3.61g (20.73 mmol, 87%).

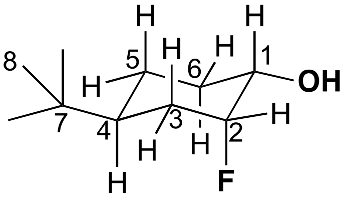


**mp** 88–91 °C (lit[3] 76 °C); **IR** 3256(m, br), 2936(s), 2869(m), 1474(s), 1385(m), 1362(m), 1270(m), 1080(m), 1012(s), 982(s) cm$^{-1}$; **$^{1}$H NMR** (400 MHz, $CDCl_3$) δ 4.68–5.02 (1H, m, *J* 51.5 can be observed, H2), 3.36–3.66 (1H, m, H1, simplifies to a ddd, *J* 28.9, 11.6, 2.7 Hz upon $D_2O$ exchange), 2.07–2.29 (1H, m, H3-eq), 1.86–1.98 (2H, m, -OH + H6-eq, integration decreases by 1 upon $D_2O$ exchange), 1.77–1.85 (1H, m, H5-eq, simplifies to a dquin, *J* 13.2, 3.4 upon $D_2O$ exchange), 1.51–1.67 (1H, m, H6-ax, simplifies to a qd, *J* 12.3, 2.5 Hz upon $D_2O$ exchange), 1.36–1.46 (1H, m, H4, simplifies to a tt, *J* 12.6, 2.8 upon $D_2O$ exchange), 1.13–1.35 (1H, m, H3-ax, simplifies to dddd, *J* 45.2, 14.3, 12.8, 1.5 Hz upon $D_2O$ exchange), 0.97–1.12 (1H, m, H5-ax), 0.88 (9H, s, H8) ppm; **$^{1}$H{$^{19}$F} NMR** (400 MHz, $CDCl_3$) δ 4.82–4.88 (1H, m, H2), 3.45–3.54 (1H, m, H1), 2.12–2.21 (1H, m, H3-eq), 1.86–1.97 (2H, m, OH + H6-eq), 1.76–1.84 (1H, m, simplifies to dquin, *J* 13.2, 3.3 Hz upon $D_2O$ exchange, H5-eq), 1.59 (1H, qd, *J* 12.5, 3.4 Hz, H6-ax), 1.35–1.45 (1H, m, simplifies to tt, *J* 12.6, 3.1 Hz upon $D_2O$ exchange, H4), 1.18–1.29 (1H, m, H3-ax), 0.99–1.11 (1H, qd, *J* 12.7, 3.4 Hz, H5-ax), 0.86 (9H, s, H8) ppm; **$^{13}$C NMR** (101 MHz, $CDCl_3$) δ 92.5 (d, *J* 171.3 Hz. C2), 71.3 (d, *J* 20.5 Hz, C1), 40.2 (C4), 31.8 (C7), 31.0 (d, *J* 20.5 Hz, C3), 29.7 (d, *J* 2.9 Hz, C6), 27.4 (C8), 24.9 (C5) ppm; **$^{19}$F NMR** (282 MHz, $CDCl_3$) δ -204.1 (dddd, *J* 51.0, 45.1, 29.0, 8.9 Hz) ppm; **MS**(ESI-) *m/z* 173.1 [M-H]$^-$.

[2] P. R. Anizelli, D. C. Favaro, R. H. Contreras, C. F. Tormena, *J. Phys. Chem. A* **2011**, *115*, 5684–5692.
[3] P. Moreau, A. Casadevall, E. Casadevall, *Bull Chem. Soc. Fr.* **1969**, 2013–2020.

The $^{19}$F NMR chemical shift, and $J_{C1\text{-}3\text{–}F}$ values correspond to the literature.[2]

In both cases, the $^{1}$H and $^{19}$F NMR spectrum of the crude product only shows one diastereoisomer:

**1.2 NMR spectra of the crude product obtained from reduction of 11**:

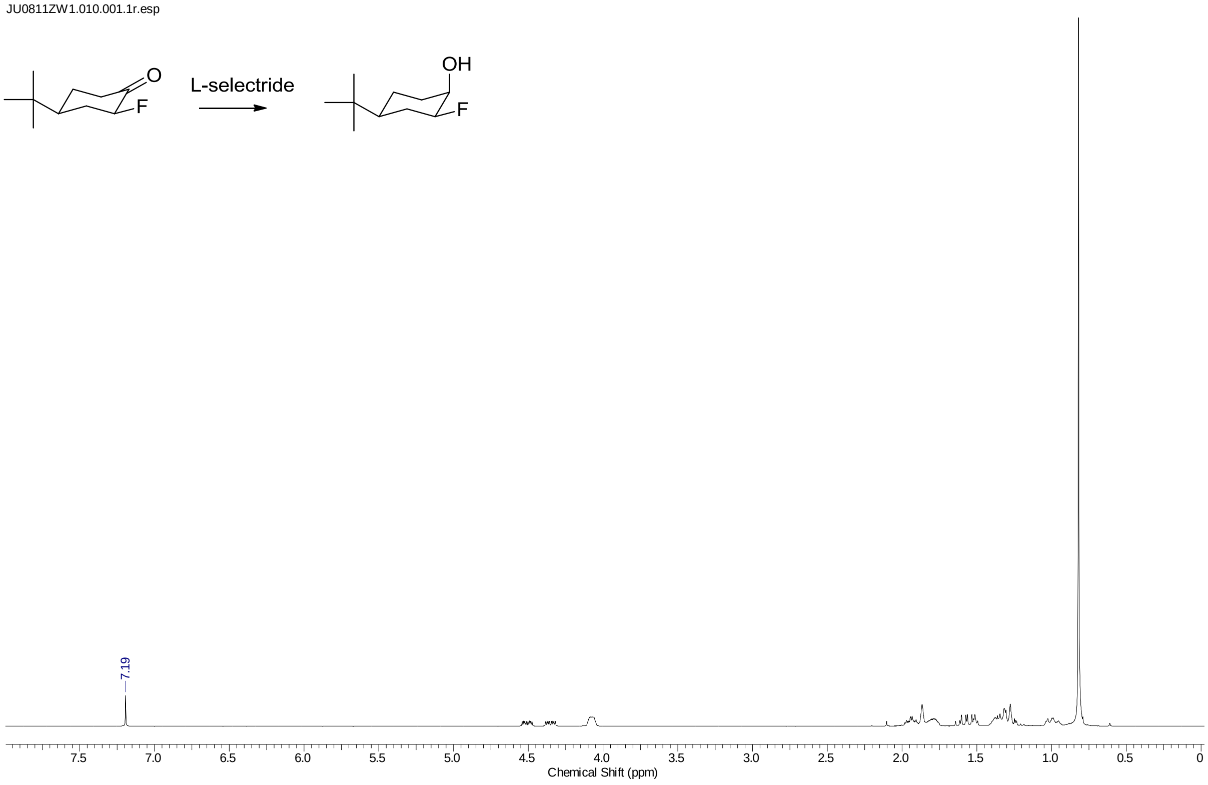


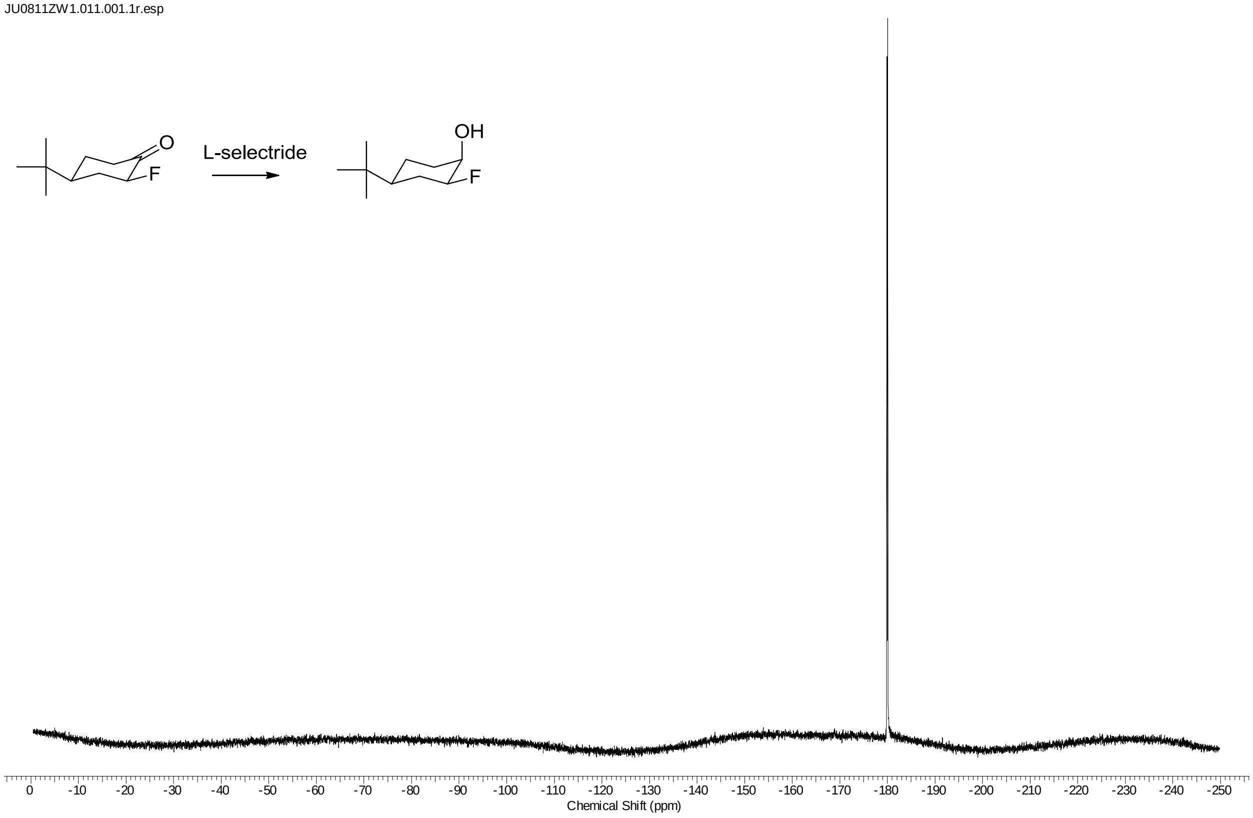

**1.3 NMR spectra of the crude product obtained from reduction of 12**:

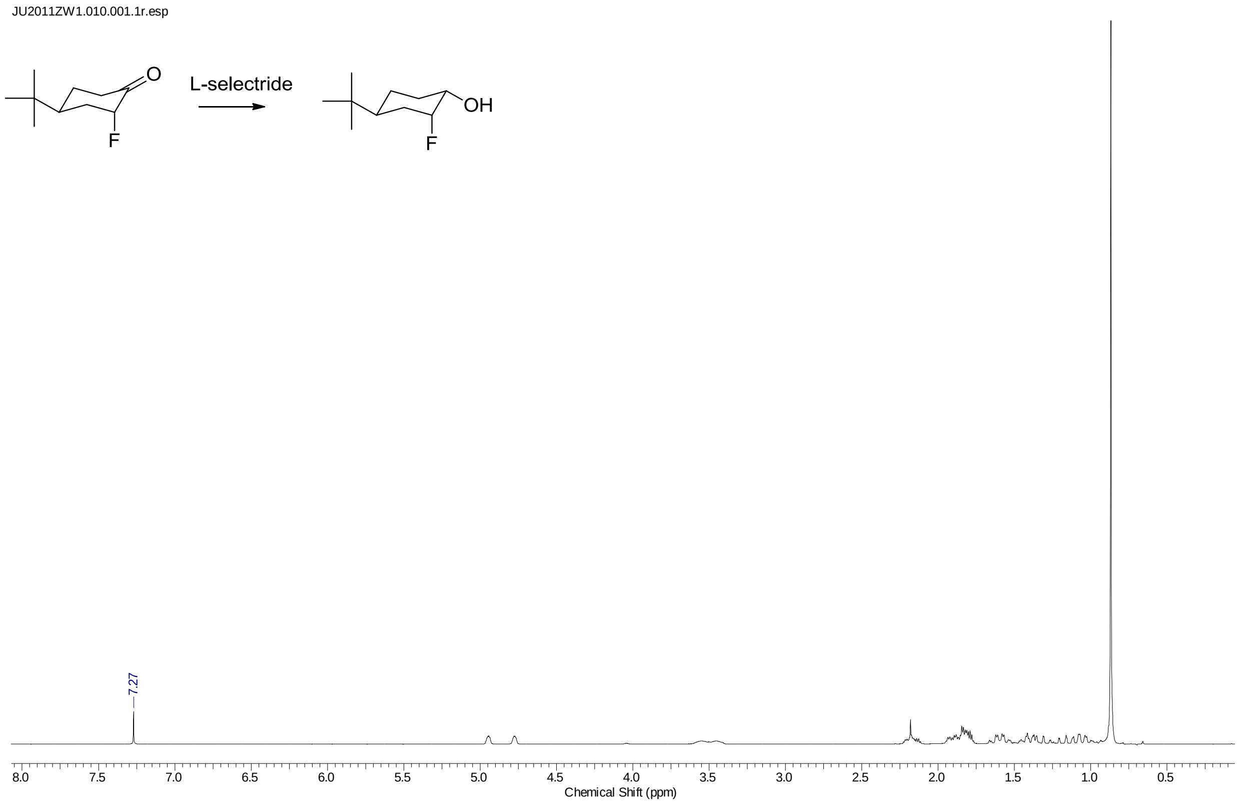


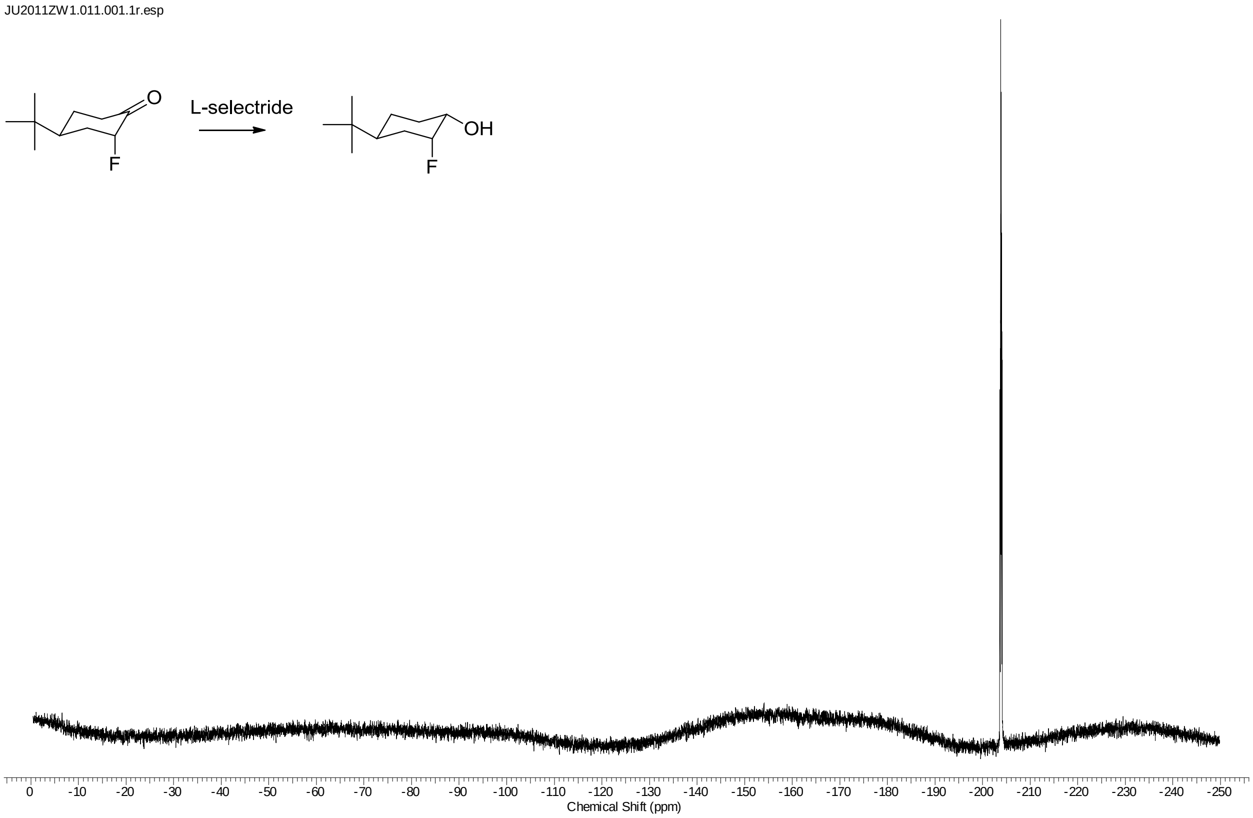

**1.4 Synthesis of fluorohydrins 1 and 4.**

**General procedures for reduction of ketone using $NaBH_4$ with EtOH in diethyl ether.**[2]

To a solution of ketone (0.3 mmol-20 mmol, 1 equiv.) in anhydrous diethyl ether was added $NaBH_4$ and several drops of ethanol. The resulting mixture was stirred overnight. The reaction was monitored by TLC. If completed, the reaction was stopped by quenching with water (6 mL/mmol), extracted with diethyl ether three times (14 mL/mmol), dried over $MgSO_4$ and concentrated. The crude was purified by flash chromatography.

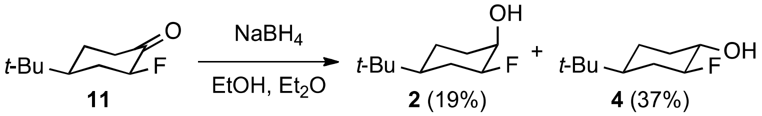


Ketone **11** (2.1 g, 12.2 mmol) was reduced according to the general procedures described above to afford **2** (400 mg, 2.30 mmol, 19%) as colourless oil and **4** (786 mg, 4.51 mmol, 37%) as white solid.

Compound **4**:

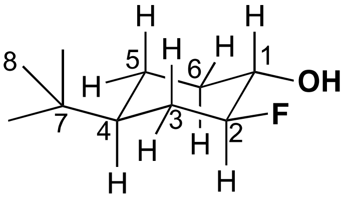


**mp** 48–52 ºC (lit[3] 47–48 °C); **IR** 3368(w, br), 2947(s), 2857(m), 1459(w), 1361(s), 1231(w), 1064(s), 1032(s), 977 (m) $cm^{-1}$; **$^1$H NMR** (400 MHz, $CDCl_3$) δ 4.17–4.38 (1H, m, a coupling of 51.9 can be observed, H2), 3.54–3.66 (1H, m, H1), 2.42 (1H, br. s, OH), 2.10–2.20 (1H, m, H3-eq), 1.99–2.09 (1H, m, H6-eq), 1.67–1.78 (1H, m, H5-eq), 0.99–1.35 (4H, m, H3-ax + H4 + H5-ax + H6-ax), 0.88 (9H, s, H8) ppm; **$^1$H{$^{19}$F} NMR** (400 MHz, $CDCl_3$) δ 4.23–4.33 (1H, m, H2), 3.55–3.65 (1H, m, H1), 2.42 (1H, br. s., OH), 2.10–2.19 (1H, m, H3-eq), 2.01–2.09 (1H, m, a coupling of 13.1 can be observed, H6-eq), 1.69–1.78 (1H, m, a coupling of 12.6 Hz can be observed, H5-eq), 1.0–1.35 (4H, m, H6-ax + H3-ax + H4 + H5-ax), 0.88 (9H, s, H8) ppm; **$^{13}$C NMR** (101 MHz, $CDCl_3$) δ 97.5 (d, *J* 174.2 Hz, C2), 73.7 (d, *J* 17.6 Hz, C1), 45.9 (d, *J* 8.8 Hz, C4), 32.2 (C7), 31.7 (d, *J* 17.6 Hz, C3), 31.0 (d, *J* 7.3 Hz, C6), 27.5 (C8), 24.7 (d, *J* 2.9 Hz, C5) ppm; **$^{19}$F NMR** (376.5 MHz, $CDCl_3$) δ -180.7 (m, a coupling of *J* 50.8 Hz can be observed) ppm; **MS**(ESI-) *m/z* 173.1 $[M-H]^-$.

The $^{19}$F NMR chemical shift, and $J_{C1\text{-}3\text{–}F}$ values correspond to the literature.[2]

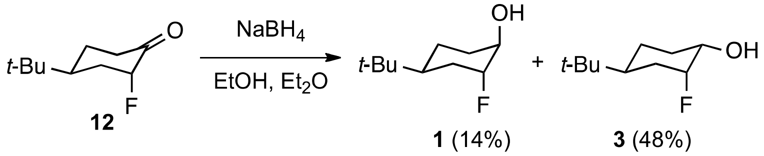


Ketone **12** (10.44 mmol) was reduced according to the general procedures described above to afford **1** (249 mg, 1.43 mmol, 14%) and **3** (868 mg, 4.98 mmol, 48%) as white solid.

Compound **1**:

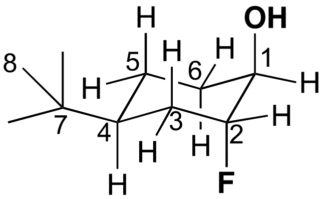


**mp** 69–70 ºC (lit[3] 61 °C); **IR** 3321(m, br), 2953(s), 2859(m), 1477(m), 1356(w), 1236(m), 1159(m), 1049(s), 1025(s), 988(s) $cm^{-1}$; **$^1$H NMR** (400 MHz, $CDCl_3$) δ 4.61–4.76 (1H, m, a coupling of 46.5 Hz can be observed, H2), 3.98 (1H, br. s, H1), 1.90–1.99 (1H, m, H3-eq), 1.72–1.88 (2H, m, H6-eq + H6-ax), 1.25–1.63 (5H, m, H5-eq + H3-ax + H4 + -OH + H5-ax, integration decreases by 1 following $D_2O$ exchange), 0.87 (9H, s, H8) ppm; **$^1$H{$^{19}$F} NMR** (400 MHz, $CDCl_3$) δ 4.66–4.72 (1H, m, H2), 3.96–4.00 (1H, br. s, H1), 1.95 (1H, br. d, *J* 14.7 Hz H3-eq), 1.73–1.88 (2H, m, H6-eq + H6-ax), 1.27–1.60 (5H, m, H5-eq + H3-ax + H4 + -OH + H5-ax), 0.87 (9H, s, H8) ppm; **$^{13}$C NMR** (101 MHz, $CDCl_3$) δ 91.0 (d, *J* 171.3 Hz, C2), 66.2 (d, *J* 29.3 Hz, C1), 40.8 (C4), 32.1 (C7), 28.6 (C6), 27.2 (C8), 27.1 (d, *J* 19.0 Hz, C3), 19.9 (C5) ppm; **$^{19}$F NMR** (282 MHz, $CDCl_3$) δ -186.0 (t, *J* 45.1 Hz) ppm; **MS**(ESI-) *m/z* 173.0 $[M-H]^-$.

The $^{19}$F NMR chemical shift, and $J_{C1\text{-}3\text{–}F}$ values correspond to the literature.[2]

## 1.5 Synthesis of 1,3-fluorohydrins 5 and 6.

### 1.5.1 Synthesis of 6

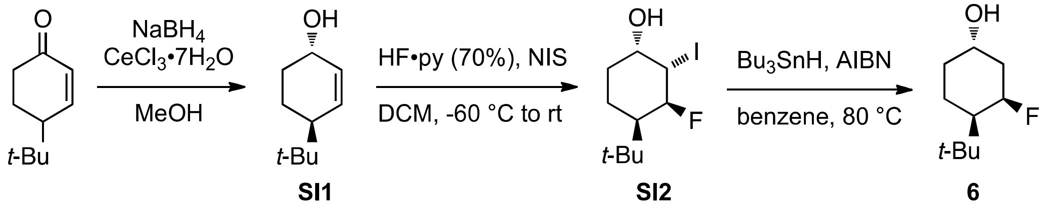


**Reduction of 4-*t*-butyl cyclohexanone to give SI1**

To a solution of 4-(*t*-butyl)cyclohex-2-enone (5.30 g, 34.8 mmol) and $CeCl_3$•$7H_2O$ (19.50 g, 52.2 mmol) in MeOH (85 mL) at 0 $^oC$ was added $NaBH_4$ (1.58 g, 41.8 mmol). The reaction was stirred at RT for 1 h, and then quenched by the addition of $H_2O$ (50 mL) and concentrated. Addition of HCl (50 mL, 1M) was followed by dilution with EtOAc (80 mL) and extraction. The organic layer was further washed with $H_2O$ (20 mL), and brine (20 mL), dried over $NaSO_4$ and concentrated in vacuo. The crude product (6.27 g) was purified by gradient column chromatography (7 → 10% acetone in petrol ether) to afford 2.99 g (56% yield) of pure product (light yellow coloured oil).

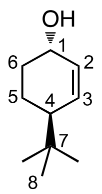


**$^1$H NMR** (400 MHz, $CDCl_3$) δ 5.69–5.80 (2H, m, H2 + H3), 4.20 (1H, br. s., H1), 2.11–2.19 (1H, m, H6'), 1.87–1.94 (1H, m, H4), 1.77–1.84 (1H, m, H5'), 1.56 (1H, br. s., OH), 1.24–1.44 (2H, m, H5'' + H6''), 0.87 (9H, s, H8) ppm. **$^{13}$C NMR** (101 MHz, $CDCl_3$) δ 132.1 (C3), 131.3 (C2), 67.7 (C1), 45.9 (C4), 33.3 (C6), 32.8 (C7), 27.1 (C8), 22.8 (C5) ppm**.**

$^1$H NMR data match with literature data.[4]

**Iodofluorination of SI1 to give SI2**

To a solution of **SI1** (2.17 g, 14.1 mmol) in DCM (20 mL) at -60 °C was added HF•py 70% (511 μL) (plastic syringe), immediately followed by NIS (6.33 g, 28.1 mmol). The reaction mixture was left in the dry ice bath for 2 h, and then allowed to warm up to room temperature while stirring for 24 h. To the reaction mixture was added a 17% solution of aq. ammonia (20 mL), and then extracted with $Et_2O$ (3 × 50 mL). The ether phases were washed with a sat. aq. $Na_2S_2O_3$ solution (30 mL), and with brine (50 mL). Following drying ($MgSO_4$), the solvent was evaporated, and the resulting crude mixture purified by gradient column chromatography (5 → 10% acetone in petrol ether) and preparative HPLC (5% acetone in hexane). This results in 1.95 g (46%) of pure **SI2** as a white solid. Other isomers were formed in much smaller amounts, but could not be obtained pure.

[4] D. Young, W. Kitching, G. Wickham, *Aust. J. Chem.* **1984**, *37*, 1841–1862.

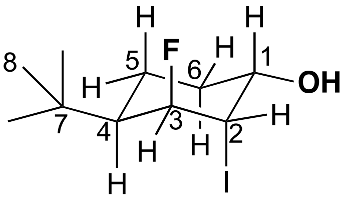


**mp** 73–75 °C; **IR** (neat) 3274 (m, br), 2949 (s), 2869 (m), 1478 (m), 1361 (m), 1270 (m), 1081 (s), 1058 (s), 960 (s), 915 (s) $cm^{-1}$; **$^1$H NMR** (400 MHz, $CDCl_3$) δ 5.24 (1H, br. d, *J* 47.7 Hz, H3), 4.77–4.84 (1H, m, H2), 2.95–3.04 (1H, m, H1), 2.07 (1H, ddd, *J* 40.9, 10.7, 4.4 Hz, H4), 1.78–1.85 (1H, br. d, *J* 12.85 Hz, H6-eq), 1.48–1.71 (4H, m, OH + 2H5 + H6-ax), 0.96 (9H, s, H8) ppm; **$^1$H{$^{19}$F} NMR** (400 MHz, $CDCl_3$) δ 5.23–5.27 (1H, m, H3), 4.78–4.82 (1H, m, H2), 2.95–3.03 (1H, m, H1), 2.04–2.11 (1H, m, H4), 1.78–1.85 (1H, m, H6-eq), 1.48–1.71 (4H, m, OH + 2H5 + H6-ax), 0.97 (9H, s, H8) ppm; **$^{13}$C NMR** (101 MHz, $CDCl_3$) δ 94.4 (d, *J* 178.6 Hz, C3), 66.7 (C1), 43.8 (d, *J* 23.4 Hz, C2), 43.1 (d, *J* 19.0 Hz, C4), 32.2 (C7), 31.8 (C6), 28.3 (C8), 20.1 (d, *J* 2.9 Hz, C5) ppm; **$^{19}$F NMR** (282 MHz, $CDCl_3$) δ -173.7 (1F, ddd, *J* 47.3, 38.7, 8.6 Hz) ppm; **MS** (EI) 173.2 (6, $M^{+\cdot}$-HI), 57.1 (100).

**Reduction of SI2 to give 1,3-fluorohydrin 6**

To a solution of **SI2** (2.54 g, 8.46 mmol) in degassed benzene (147 mL) was added $Bu_3SnH$ (4.6 mL, 16.9 mmol) and AIBN (111 mg, 0.68 mmol). The mixture was heated to reflux for 3 h, after which the solvent was removed under vacuo. The residue was purified by gradient column chromatography (9% → 15% acetone in petrol ether) to give 1.28 g (87%) of **6** as a white solid.

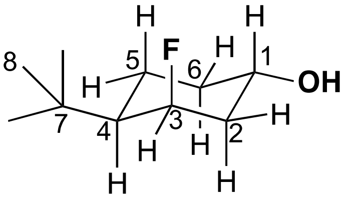


**mp** 65–70 °C; **IR** (neat) 3282 (m, br.), 2949 (s), 1481 (m), 1363 (s), 1063 (s), 734 (s) $cm^{-1}$; **$^1$H NMR** (400 MHz, $CDCl_3$) δ 5.12 (1H, d, *J* 48.8 Hz, H3), 3.91–4.01 (1H, m, H1), 2.31–2.41 (1H, m, H2-eq), 2.05–2.13 (1H, m, a coupling of 11.9 Hz can be observed, H6-eq), 1.75–1.65 (1H, m, H5-eq), 1.61 (1H, qd, *J* 13.1, 2.9 Hz), 1.56 (1H, d, *J* 4.3 Hz, OH), 1.36 (1H, dt, *J* 44.3, 12.4 Hz, H2-ax), 1.23–1.35 (1H, m, H6-ax), 1.06 (1H, ddd, *J* 40.3, 12.3, 3.4 Hz), 0.96 (9H, s, H8) ppm; **$^1$H{$^{19}$F} NMR** (400 MHz, $CDCl_3$) δ 5.13 (1H, br. s, H3), 3.92–4.01 (1H, m, simplifies to tt, *J* 11.2, 4.8 Hz upon $D_2O$ exchange, H1), 2.36 (1H, br. d, *J* 12.4 Hz, H2-eq), 2.06–2.13 (1H, m, a coupling of 12.1 Hz can be observed, H6-eq), 1.66–1.75 (1H, m, H5-eq), 1.61 (1H, qd, *J* 13.1, 3.0 Hz, H5-ax), 1.52 (1H, br. s., OH), 1.35–1.41

(1H, m, H2-ax), 1.29 (1H, qd, *J* 12.3, 3.5 Hz, H6-ax), 1.01–1.09 (1H, m, H4), 0.96 (9H, s, H8) ppm; **$^{13}$C NMR** (101 MHz, $CDCl_3$) δ 91.2 (d, *J* 171.3 Hz, C3), 66.5 (C1), 50.0 (d, *J* 19.0 Hz, C4), 41.2 (d, *J* 20.5 Hz, C2), 35.5 (C6), 32.3 (C7), 28.5 (C8), 19.9 (d, *J* 2.9 Hz, C5) ppm; **$^{19}$F NMR** (282 MHz, $CDCl_3$) δ -193.0– -192.5 (m, a coupling of 8.6 Hz can be observed) ppm; **MS** (EI) 154.2 (1.4, $M^{+\cdot}$-HF), 57.2 (100).

### 1.5.2 Synthesis of 5

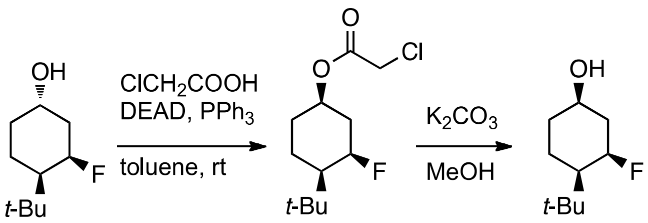


**6** **SI3** **5**

**Mitsunobu reaction of 6 to give SI3**

To a solution of **6** (1.16g, 6.66 mmol) in toluene (66 mL) was added triphenyl phosphine (3.49 g, 13.3 mmol) and chloroacetic acid (1.26 g, 13.3 mmol). The mixture was cooled to 0 °C, and after DEAD (2.09 mL, 13.3 mmol) was added dropwise, it was left stirring at room temperature for 18 h. The solvent was removed in vacuo, and the resulting mixture was directly purified by gradient column chromatography (2% → 10% acetone in petrol ether). This gave 1.08 g of ester **SI3** (65%) as a colourless oil.

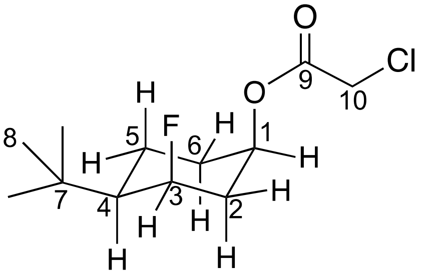


**IR** (neat) 2952 (s), 1746 (s), 1368 (m), 1312 (s), 1194 (s), 1168 (s), 1081 (s) $cm^{-1}$; **$^1$H NMR** (400 MHz, $CDCl_3$) δ ppm 4.96–5.14 (2H, m, H3 + H1), 4.08 (2H, s, H10), 2.37–2.48 (1H, m, H2-eq), 2.03–2.12 (1H, m, H6-eq), 1.82–1.94 (1H, m, H5-ax), 1.52–1.74 (3H, m, H2-ax, H5-eq, H6-ax), 1.09 (1H, br. dd, *J* 39.9, 11.4 Hz, H4), 0.98 (9H, s, H8) ppm; **$^1$H{$^{19}$F} NMR** (400 MHz, $CDCl_3$) δ 5.11 (1H, br. s, H1), 5.04 (1H, br. s, H3), 4.08 (2H, s, H10), 2.43 (1H, br. d, *J* 16.7, H2-eq), 2.04–2.12 (1H, m, a coupling of 14.0 Hz can be observed, H6-eq), 1.82–1.94 (1H, m, H5-ax), 1.52–1.69 (3H, m, H2-ax, H5-eq, H6-ax), 1.09 (1H, br. d, *J* 12.7 Hz, H4), 0.98 (9H, s, H8) ppm; **$^{13}$C NMR** (101 MHz, $CDCl_3$) δ ppm 167.1 (C9), 88.8 (d, *J* 174.2 Hz, C3), 70.1 (C1), 50.1 (d, *J* 20.5 Hz, C4), 41.4 (C10), 34.9 (d, *J* 20.5 Hz, C2), 32.6 (C7), 30.4 (C6), 28.3 (C8), 16.1 (C5) ppm; **$^{19}$F NMR** (282 MHz, $CDCl_3$) δ -192.2– -191.7 (m, a coupling of 12.9 Hz can be observed) ppm.

**Methanolysis of SI3 to give 5**

To a solution of **SI3** (627 mg, 2.50 mmol) in MeOH (10 mL) was added $K_2CO_3$ (346 mg, 2.50 mmol), and left stirring for 3 h. A half-saturated aqueous $NH_4Cl$ solution (30 mL) was added, and the mixture was four times extracted with DCM (30 mL each). The combined DCM phases were washed with brine (50 mL), dried ($MgSO_4$), and the solvent evaporated in vacuo. The residue was purified by column chromatography (5% acetone in petrol ether) to give **5** (281 mg, 65%) as a white solid.

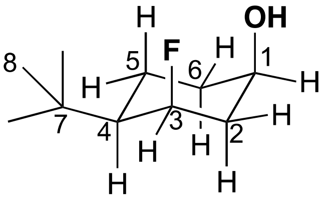


**mp** 57–60 °C; **IR** (neat) 3299 (m, br.), 2940 (s), 2903 (m), 1360 (m), 1313 (m), 1102 (s), 1090 (s), 942 (s) cm$^{-1}$; **$^1$H NMR** (400 MHz, $CDCl_3$) δ 5.20 (1H, br. d, *J* 49.1 Hz, H3), 3.96–4.04 (1H, m, H1), 2.48 (1H, dd, *J* 12.1, 9.5 Hz, OH), 2.28–2.38 (1H, m, H2-eq), 1.99–2.08 (1H, m, a coupling of 13.6 Hz can be observed, H6-eq), 1.87 (1H, qd, *J* 13.3, 2.9 Hz, H5-ax), 1.46–1.74 (3H, m, H2-ax, H5-eq, H6-ax), 1.09 (1H, dd, *J* 42.3, 13.6 Hz, H4), 0.97 (9H, s, H8) ppm; **$^1$H{$^{19}$F} NMR** (400 MHz, $CDCl_3$) δ 5.17–5.23 (1H, m, H3), 3.96–4.03 (1H, m, H1), 2.49 (1H, d, *J* 8.6 Hz, OH), 2.33 (1H, br. d, *J* 15.7 Hz, H2-eq), 1.99–2.07 (1H, m, a coupling of 13.7 Hz can be observed, H6-eq), 1.87 (1H, qd, *J* 13.3, 2.9 Hz, H5-ax), 1.46–1.74 (3H, m, H2-ax + H5-eq + H6-ax), 1.10 (1H, br. t, *J* 11.5 Hz, H4), 0.97 (9H, s, H8) ppm; **$^{13}$C NMR** (101 MHz, $CDCl_3$) δ 92.6 (d, *J* 166.9 Hz, C3), 65.9 (C1), 50.4 (d, *J* 19.0 Hz, C4), 37.7 (d, *J* 19.0 Hz, C2), 33.2 (C6), 32.6 (C7), 28.3 (d, *J* 2.9 Hz, C8), 15.3 (d, *J* 2.9 Hz, C5) ppm; **$^{19}$F NMR** (282 MHz, $CDCl_3$) δ -189.3– -188.7 (m) ppm; **MS** (EI) 154.2 (3.7, $M^{+\cdot}$-HF), 57.2 (100).

### 1.6 Synthesis of 2,2-difluoroalcohols 7 and 8

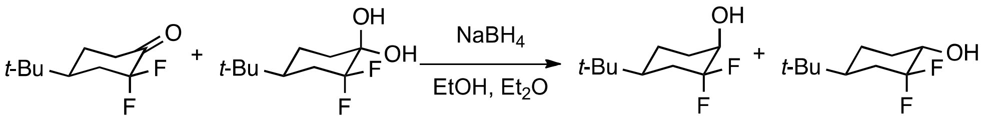


To a solution of **SI4** and **SI5**[1] (2.60 g, 13.67 mmol, 1 equiv.) in dry diethyl ether (100 mL, 7.5 mL/mmol) was added $NaBH_4$ (1.03 g, 27.34 mmol, 2 equiv.) and several drops of ethanol. The reaction mixture was stirred overnight at room temperature and then monitored by TLC. Upon completion, the reaction mixture was quenched with water (100 mL) and extracted with diethyl ether (3 × 200 mL). The combined organic layer was washed with brine, dried over $MgSO_4$ and concentrated. The resulting crude was purified by column chromatography and HPLC to afford **7** (236 mg, 2.00 mmol, 9%) and **8** (2.04 g, 10.61 mmol, 77%), both as a white solid.

Difluorohydrin **7**

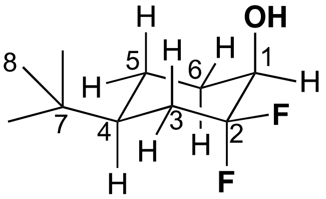


**mp** 43–45 °C; **IR** 3415(m, br), 2960(s), 2869(m), 1371(m), 1238(m), 1137(m), 1088(s), 1063(s), 1032(s) $cm^{-1}$; **$^{1}$H NMR** (400 MHz, $CDCl_3$) δ 3.86–3.92 (1H, m, H1), 2.02–2.06 (1H, m, -OH), 1.92–2.02 (2H, m, H3-eq + H6-eq), 1.78 (1H, ddt, *J* 36.6, 12.6, 4.2 Hz, H3-ax), 1.58–1.71 (1H, m, H6-ax), 1.47–1.57 (1H, m, H5-eq), 1.30–1.45 (2H, m, H4 + H5-ax), 0.90 (9H, s, H8) ppm; **$^{1}$H{$^{19}$F} NMR** (400 MHz, $CDCl_3$) δ 3.88–3.91 (1H, m, H1), 2.03 (1H, t, *J* 2.2 Hz, OH), 1.98–2.01 (1H, m, H3-eq), 1.94–1.97 (1H, m, H6-eq), 1.73–1.82 (1H, m, H3-ax), 1.61–1.71 (1H, m, H6-ax), 1.48–1.57 (1H, m, H5-eq), 1.30–1.45 (2H, m, H4 + H5-ax), 0.90 (9H, s, H8) ppm; **$^{13}$C NMR** (101 MHz, $CDCl_3$) δ 124.2 (dd, *J* 245.9, 243.0 Hz, C2), 67.9 (dd, *J* 35.1, 23.4 Hz, C1), 44.3 (d, *J* 8.8 Hz, C4), 32.1 (C7), 30.4 (dd, *J* 23.4, 20.5 Hz, C3), 28.9 (d, *J* 5.9 Hz, C6), 27.3 (C8), 19.1 (C5); **$^{19}$F NMR** (282 MHz, $CDCl_3$) δ ppm -101.7 (d, *J* 247.1 Hz, Feq), -107.1– -105.9 (m, couplings of 247.2 and 36.5 Hz can be observed, Fax).

Difluorohydrin **8**

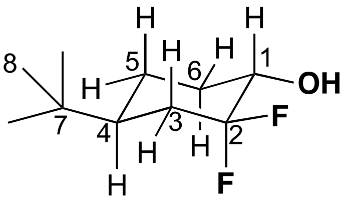


**mp** 49–50 ºC; **IR** 3346(m, br), 2959(m), 2872(m), 1365(s), 1185(m), 1171(m), 1106(s), 1081(s), 941(s) $cm^{-1}$; **$^{1}$H NMR** (300 MHz, $CDCl_3$) δ 3.59–3.70 (1H, ddt, *J* 20.2, 12.0, 4.9 Hz, H1), 2.55 (1H, br. s., OH), 2.11–2.21 (1H, m, H3'), 1.99–2.07 (1H, m, H6'), 1.74–1.82 (1H, m, a coupling of 13.3 Hz can be observed, H5'), 1.29–1.52 (3H, m, H6'' + H3'' + H4), 1.01–1.13 (1H, m, H5''), 0.87 (9H, s, H8) ppm; **$^{1}$H{$^{19}$F} NMR** (400 MHz, $CDCl_3$) δ 3.65 (1H, dd, *J* 12.0, 5.3 Hz, H1), 2.55 (1H, br. s., OH), 2.11–2.21 (1H, m, H3'), 1.99–2.06 (1H, m, H6-eq), 1.74–1.82 (1H, m, H5'), 1.46 (1H, qd, *J* 13.2, 3.8 Hz, H6-ax), 1.32–1.42 (2H, m, H3'' + H4), 1.00–1.13 (1H, m, H5''), 0.86 (9H, s, H8) ppm; **$^{13}$C NMR** (101 MHz, $CDCl_3$) δ ppm 122.9 (t, *J* 244.4 Hz, C2), 71.8 (t, *J* 22.0 Hz, C1), 44.3 (d, *J* 7.3 Hz, C4), 34.5 (dd, *J* 24.9, 20.5 Hz, C3), 31.9 (C7), 30.7 (d, *J* 7.3 Hz, C6), 27.3 (C8), 24.4 (C5); **$^{19}$F NMR** (282 MHz, $CDCl_3$) δ -103.6 (d, *J* 232.1 Hz, Feq), -122.0– -120.0 (m, a coupling of 232.1 Hz can be observed, Fax) ppm.

## 2. H-bond acidity measurements

Spectroscopic grade carbon tetrachloride was dried over freshly activated 4 Å molecular sieves. Owing to the observed weak stability with light, *N*-methylpyrrolidinone (NMP) 99.9+% was as well dried on 4 Å molecular sieves and stored in the dark. The synthesized *c*-hexanols were also carefully dried. Handling of the solutes and solvents was carried out in a glove box under dry atmosphere at room temperature. All these precautions allow the determination of the H-bond acidity of alcohols by avoiding the presence of water, a competitive H-bond donor and acceptor.

Infrared spectra were recorded with a Fourier transform spectrometer Bruker Tensor 27 at a resolution of 1 $cm^{-1}$. Before H-bond acidity measurements, we carried out the spectroscopic characterisation of the OH bond of each *c*-hexanol in $CCl_4$ using infrasil quartz cell of 1 cm path length. A Peltier effect regulation thermostatted the temperature at 25°C ± 0.2°C. The stretching vibration $\nu_{OH}$, the half-width $\Delta\nu_{1/2}$, the molar absorption coefficient $\varepsilon_{OH}$, the frequency shift upon H-bond complexation with *N*-methylpyrrolidinone $\Delta\nu_{OH}$ were determined for the synthesised compounds and are reported in Table S1. No fluorohydrin self-association was observed at the concentration employed.

Measurements of H-bonding complexation were carried out following the standard procedure established for H-bond basicity measurements,[5] and choosing NMP as the reference H-bond acceptor. For dilute ternary *c*-hexanol-NMP-$CCl_4$ solutions, the absorbance decrease of the characterised OH absorption of *c*-hexanols was followed at 25°C, leading to p$K_{AHY}$ values, where A denotes for the studied *c*-hexanol and Y for the H-bond acceptor reference, NMP.

***Table S1.*** Spectroscopic characteristics of the stretching vibration $\nu_{OH}$ of the studied *c*-hexanols.

| Entry | $\nu_{OH}$ [a] | $\Delta\nu_{1/2}$ [a] | $\varepsilon_{OH}$ [b] | $\nu_{OH\cdots B}$ [a] | $\Delta\nu_{OH}$ [a] |
|---|---|---|---|---|---|
| **9** | 3627 | 15 | 79 | 3454 | 173 |
| **10** | 3623 | 19 | 67 | 3445 | 178 |
| **1** | 3631 | 17 | 99 | 3417 | 214 |
| **2** | 3613 | 16 | 155 | 3419 | 194 |
| **3** | 3606 | 15 | 124 | 3415 | 191 |
| **4** | 3616 | 16 | 130 | 3419 | 197 |
| **5** | 3619 | 16 | 136 | --- | --- |
| **6** | 3626 | 17 | 80 | 3436 | 190 |
| **7** | 3618 | 17 | 148 | 3385 | 233 |
| **8** | 3612 | 21 | 115 | 3380 | 232 |

[a] in $cm^{-1}$. [b] in L $mol^{-1}$ $cm^{-1}$.

[5] C. Ouvrard, M. Berthelot, C. Laurence, *J. Chem. Soc., Perkin Trans. 2* **1999**, 1357–1362.

## 3. Quantum chemistry calculations

Theoretical calculations were performed using the Gaussian 09[6] program.

**3.1 Conformational study**. Owing to their excellent performance-to-cost ratio, DFT methods constitute very appealing approaches. In the present work, we selected the MPWB1K functional[7] which has been proven to surpass other functionals for energy-barrier prediction and non-bonded interactions[7] associated to the 6-31+G(d,p) atomic basis set to perform our conformational investigations in the gas phase. The harmonic frequencies were computed analytically in order to characterise the stationary points and to estimate the thermodynamic corrections derived from the MPWB1K/6-31+G(d,p) energies. The relative population of each conformer (Table S2) was evaluated from the Gibbs energies considering a Boltzmann distribution according to relation (1):

$$p_i = \frac{e^{-\Delta G_i / RT}}{\sum_{i=1}^{n} e^{-\Delta G_i / RT}} \qquad (1)$$

The influence of bulk solvent effects (here carbon tetrachloride, as in the experiments) has been simulated through the Polarisable Continuum Model (PCM).[8] It appears that no fundamental differences are found between relative populations of conformers calculated *in vacuo* Table S1) or in $CCl_4$ as solvent, although the chelated conformations are slightly less populated.

[6] M. J. T. Frisch, G. W.; Schlegel, H. B.; Scuseria, G. E.; Robb, M. A.; Cheeseman, J. R.; Scalmani, G.; Barone, V.; Mennucci, B.; Petersson, G. A.; Nakatsuji, H.; Caricato, M.; Li, X.; Hratchian, H. P.; Izmaylov, A. F.; Bloino, J.; Zheng, G.; Sonnenberg, J. L.; Hada, M.; Ehara, M.; Toyota, K.; Fukuda, R.; Hasegawa, J.; Ishida, M.; Nakajima, T.; Honda, Y.; Kitao, O.; Nakai, H.; Vreven, T.; Montgomery, J., J. A.; Peralta, J. E.; Ogliaro, F.; Bearpark, M.; Heyd, J. J.; Brothers, E.; Kudin, K. N.; Staroverov, V. N.; Kobayashi, R.; Normand, J.; Raghavachari, K.; Rendell, A.; Burant, J. C.; Iyengar, S. S.; Tomasi, J.; Cossi, M.; Rega, N.; Millam, J. M.; Klene, M.; Knox, J. E.; Cross, J. B.; Bakken, V.; Adamo, C.; Jaramillo, J.; Gomperts, R.; Stratmann, R. E.; Yazyev, O.; Austin, A. J.; Cammi, R.; Pomelli, C.; Ochterski, J. W.; Martin, R. L.; Morokuma, K.; Zakrzewski, V. G.; Voth, G. A.; Salvador, P.; Dannenberg, J. J.; Dapprich, S.; Daniels, A. D.; Farkas, O.; Foresman, J. B.; Ortiz, J. V.; Cioslowski, J.; Fox, D. J., RevA.02 ed., Gaussian, Inc., Wallingford CT, **2009**.

[7] Y. Zhao, D. G. Truhlar, *J. Phys. Chem. A* **2004**, *108*, 6908–6918.

[8] J. Tomasi, B. Mennucci, R. Cammi, *Chem. Rev.* **2005**, *105*, 2999–3093.

***Table S2.*** Relative populations of the 4-*t*-butyl *c*-hexanol derivatives in the gas phase and in $CCl_4$. Electrostatic potential $V_\alpha$(r) computed *in vacuo* for each conformer.

| Entry | *Trans* | | *g-* | | *g+* | |
|---|---|---|---|---|---|---|
| | $p_i$[a] | $V_\alpha$(r) [b] | $p_i$[a] | $V_\alpha$(r) [b] | $p_i$[a] | $V_\alpha$(r) [b] |
| **9** | 24.9% (25.5%) | 0.3151 | 75.1% (74.5%) [d] | 0.3194 | [d] | |
| **10** | 26.8% (31.1%) | 0.3167 | 73.2% (68.9%) [d] | 0.3181 | [d] | |
| **1** | 12.5% (12.3%) | 0.3277 | 54.4% (49.4%) | 0.3315 | 33.0% (38.3%) | 0.3328 |
| **2** | 0.2% (0.7%) | 0.3238 | 1.3% (2.7%) | 0.3280 | **98.4% (96.6%)** | 0.3124 |
| **3** | **97.5% (93.1%)** | 0.3104 | 0.9% (3.8%) | 0.3248 | 1.6% (3.2%) | 0.3250 |
| **4** | 0.7% (1.9%) | 0.3244 | 1.2% (2.6%) | 0.3258 | **98.1% (95.4%)** | 0.3131 |
| **5** | **99.7% ()** | 0.2914 | 0.3% () | 0.3206 | [f] | |
| **6** | 16.4% () | 0.3224 | 47.0% () | 0.3228 | 36.6% () | 0.3228 |
| **7** | 0.2% (0.7%) | 0.3340 | 2.0% (4.1%) | 0.3374 | **97.8% (95.2%)** | 0.3260 |
| **8** | **43.4% (48.9%)** | 0.3181 | 0.5% (1.9%) | 0.3317 | **56.1% (49.2%)** | 0.3202 |

[a] in vacuo (in $CCl_4$). [b] in a.u..

**3.2 H-bond acidity characterisation**. Since the original works of Murray and Politzer,[9,10] it has been consistently demonstrated that H-bond acidity and the electrostatic potential are rather well correlated, owing to the mainly electrostatic character of the H-bond interaction. Kenny has recently introduced and recommended[11] the $V_\alpha$(r) descriptor for such correlations. $V_\alpha$(r) is defined as the molecular electrostatic potential calculated at a distance, $r$ = 0.55Å, from the donor hydrogen atom on the axis defined by the nuclei of the hydrogen atom and the atom to which it was bonded. The $V_\alpha$(r) values were calculated on the OH hydrogen atom of studied *c*-hexanols, and weighted by the conformers Boltzmann populations. These results are indeed found to be strongly correlated to the experimental H-bond acidity as shown in the manuscript.

Some of these compounds are likely to give intramolecular interactions between the OH group and the fluorine atom. Those interactions were characterised from electron densities computed at the critical points of the corresponding H-bond, within the framework of Atoms in Molecules (AIM) theory [12,13] as implemented in the AIM2000 program.[14] A Natural Bond Orbital (NBO)[15,16] analysis was also carried

9 J. S. Murray, P. Politzer, *J. Chem. Res., Synop.* **1992**, 110–111.
10 H. Hagelin, J. S. Murray, T. Brinck, M. Berthelot, P. Politzer, *Can. J. Chem.* **1995**, *73*, 483–488.
11 P. W. Kenny, *J. Chem. Inf. Model.* **2009**, *49*, 1234–1244.
12 R. F. W. Bader, *Chem. Rev.* **1991**, *91*, 893–928.
13 R. F. W. Bader, *Atoms in Molecules: A Quantum Theory*, Clarendon, Oxford, **1994**.
14 F. W. Biegler-Koenig, J. Schonbohm, D. Bayles, *J. Comput. Chem.* **2001**, *22*, 545–559.
15 A. E. Reed, L. A. Curtiss, F. Weinhold, *Chem. Rev.* **1988**, *88*, 899–926.

out, considering the interaction energy, $E^{(2)}_{n\rightarrow\sigma^*}$, between the occupied non-bonded orbital of the fluorine atom, $n_F$, and the unoccupied anti-bonding orbital of the OH bond, $\sigma^*_{OH}$.

***Table S3.*** Estimation of the loss of H-bond acidity in fluorohydrins in which an F···HO interaction can occur.

| Entry | *Trans* | | *g-* | | *g+* | |
|---|---|---|---|---|---|---|
| | p$K_{AHY}$(calc) [a] | $\delta\Delta G$ [b] | p$K_{AHY}$(calc) [a] | $\delta\Delta G$ [b] | p$K_{AHY}$(calc) [a] | $\delta\Delta G$ [b] |
| **2** | 0.95 | +3.0 | 1.10 | +3.8 | | |
| **3** | | | 0.98 | +2.7 | 0.99 | +2.5 |
| **4** | 0.98 | +2.4 | 1.02 | +2.6 | | |
| **5** | | | 0.84 | +5.6 | | |
| **7** | 1.32 | +1.6 | 1.44 | +2.3 | | |
| **8** | | | 1.24 | +2.2 | | |

[a] p$K_{AHY}$(calc) = 35.88$V_{\alpha}$(r) -10.67. [b] $\delta\Delta G$ = -5.708 (p$K_{AHY}$(exp) - p$K_{AHY}$(calc)).

[16] E. D. Glendening, A. E. Reed, J. E. Carpenter, F. Weinhold, Theoretical Chemistry Institute, University of Wisconsin, Madison.

## 4. Copies of $^{1}$H, $^{13}$C, and $^{19}$F NMR spectra (in numerical order)

## 4.1 Fluorohydrin 1

### $^{1}$H NMR (400 MHz)

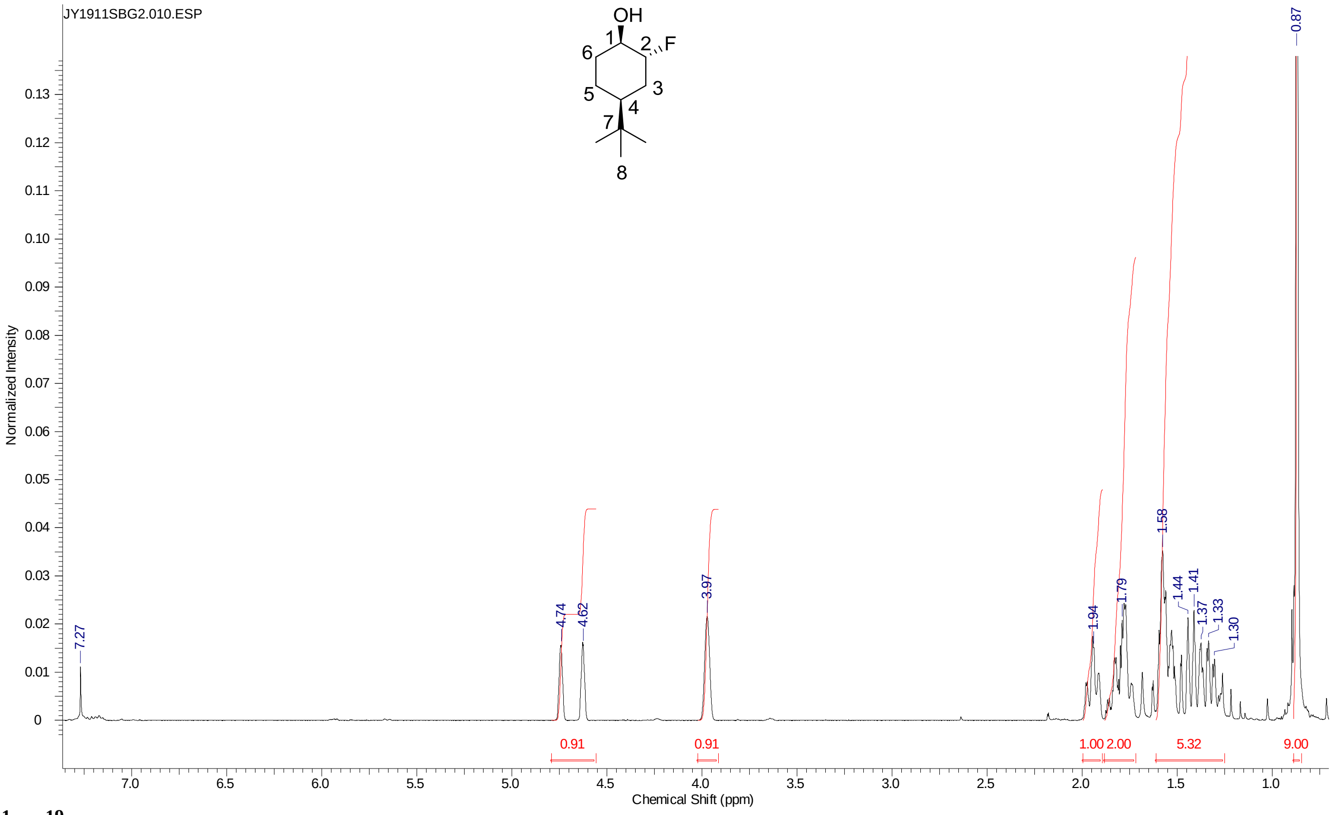


### $^{1}$H{$^{19}$F} NMR (400 MHz)

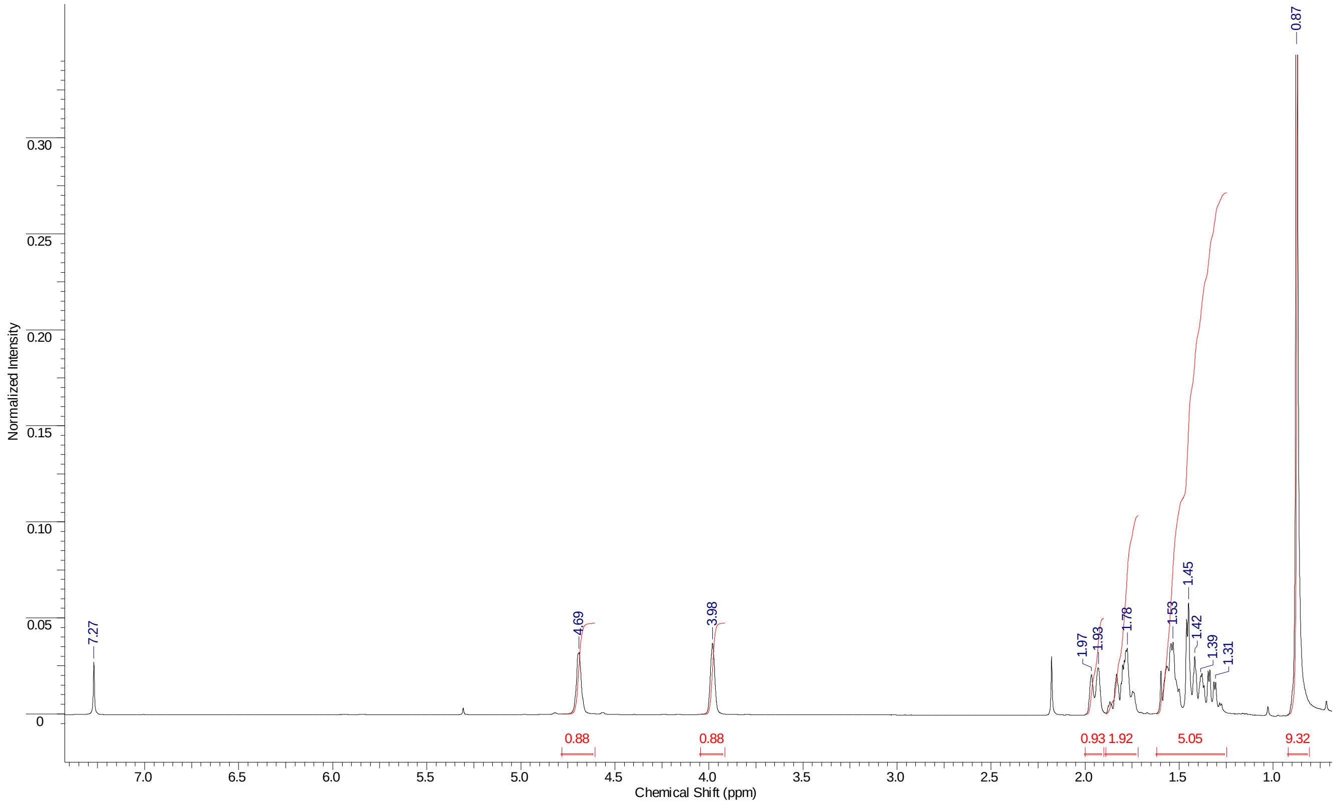

## $^{13}$C NMR (101 MHz)

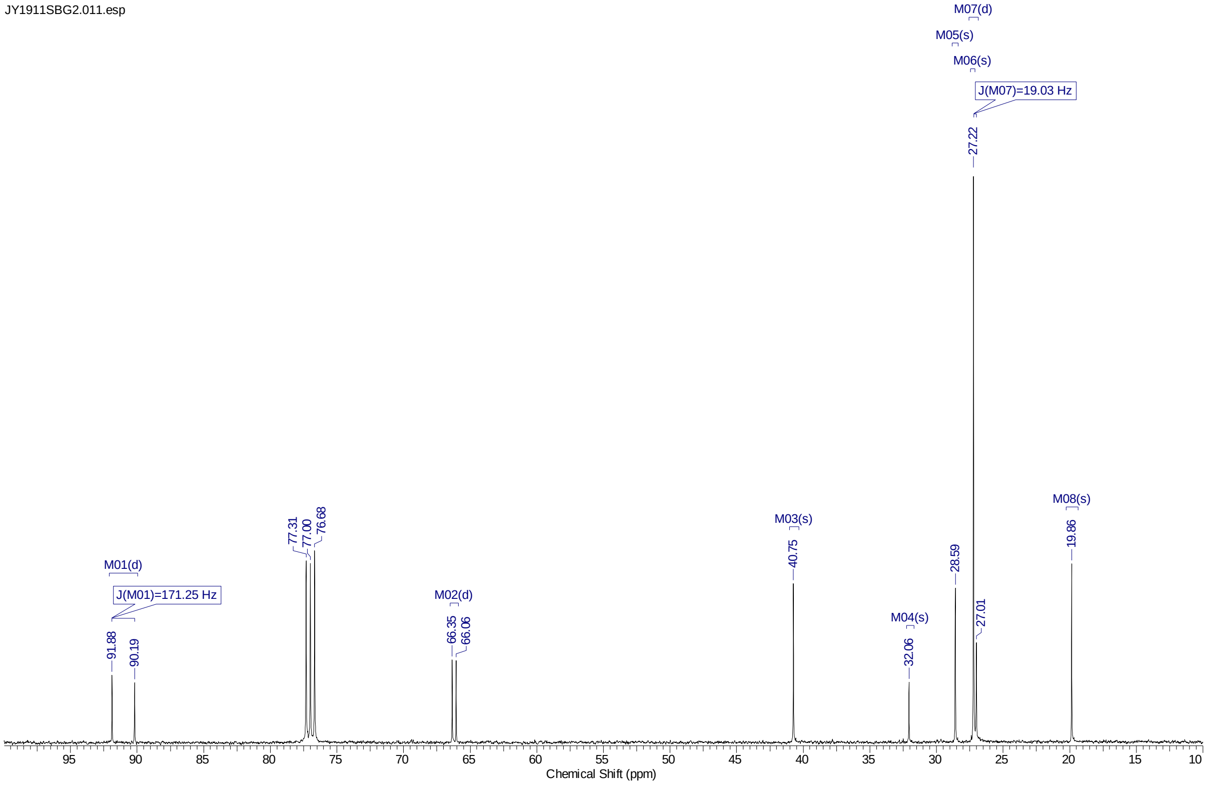


## $^{19}$F NMR (282 MHz)

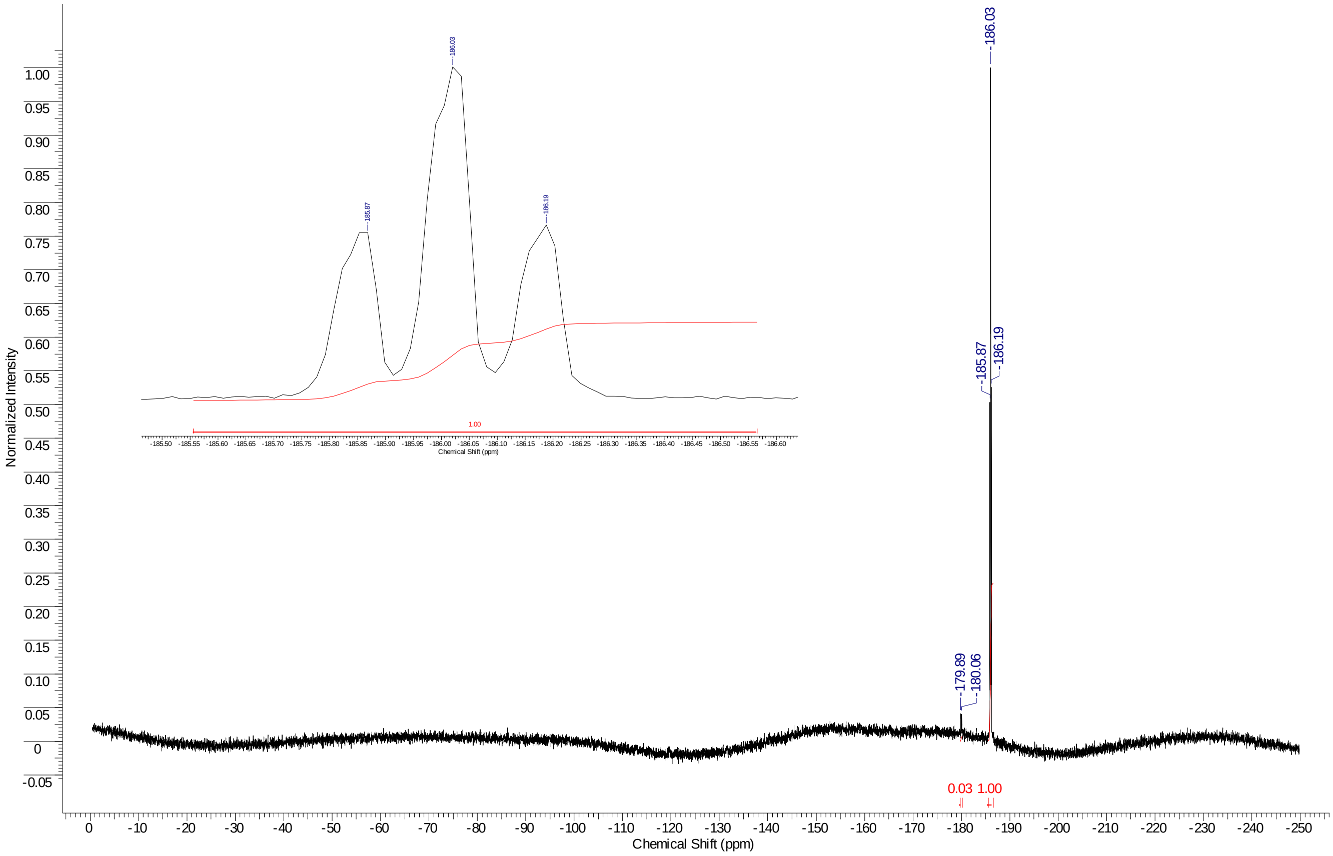

## 4.2 Fluorohydrin 2

## $^1$H NMR (400 MHz)

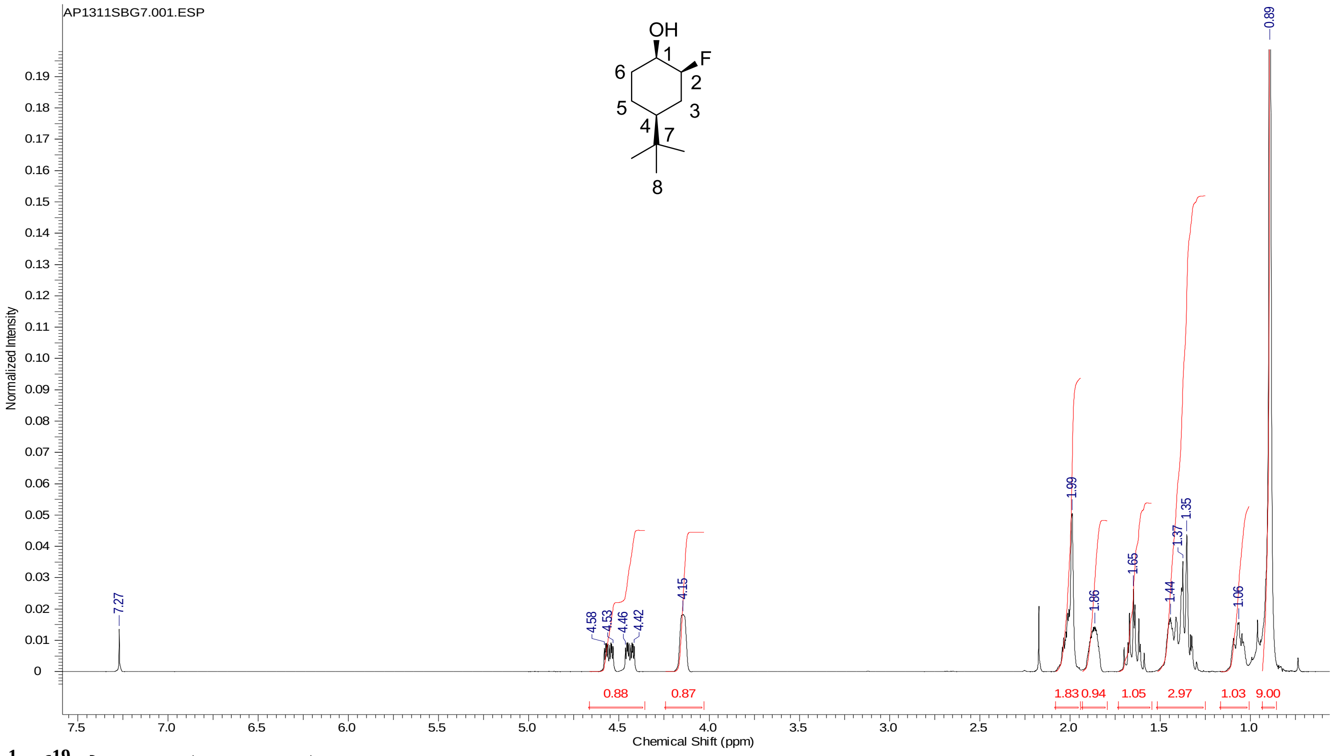


## $^1$H{$^{19}$F} NMR (400 MHz)

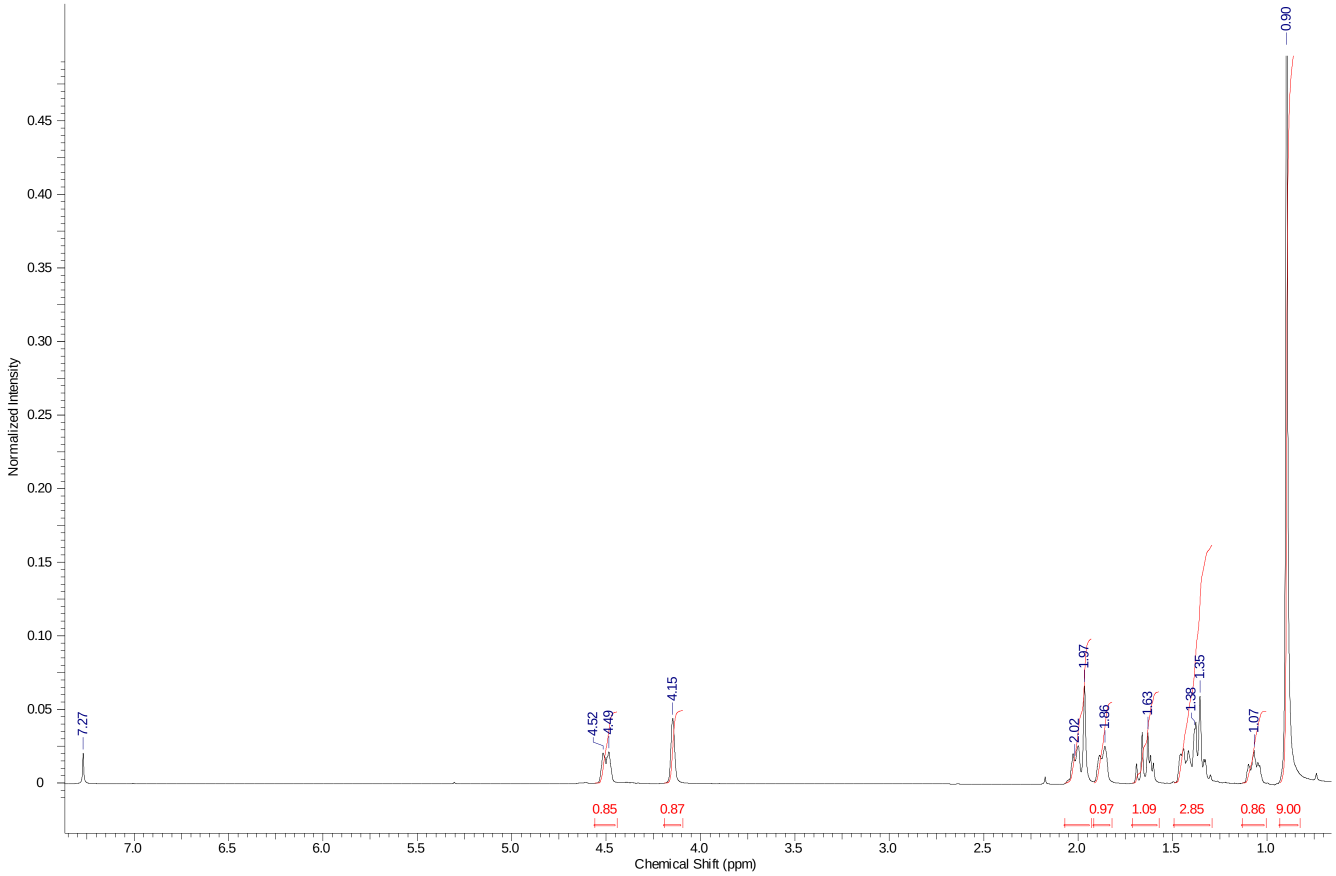

## $^{13}$C NMR (75 MHz)

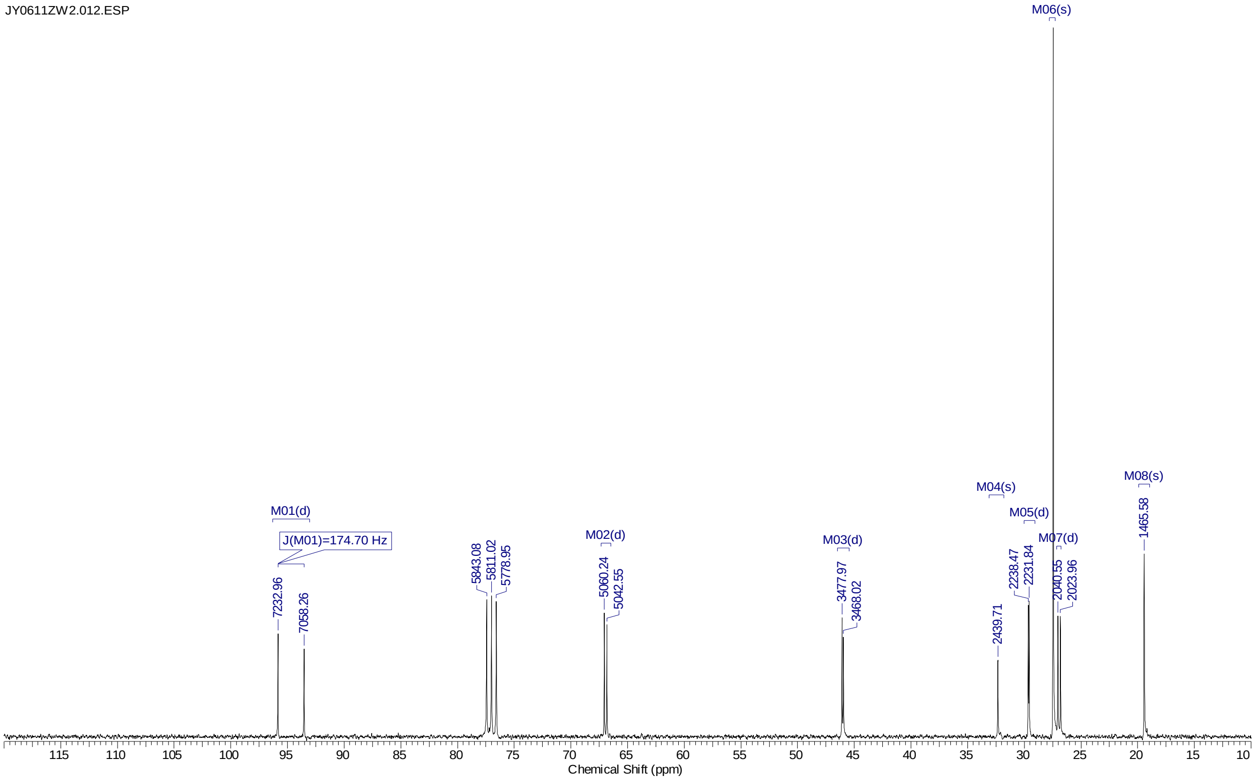


## $^{19}$F NMR (282 MHz)

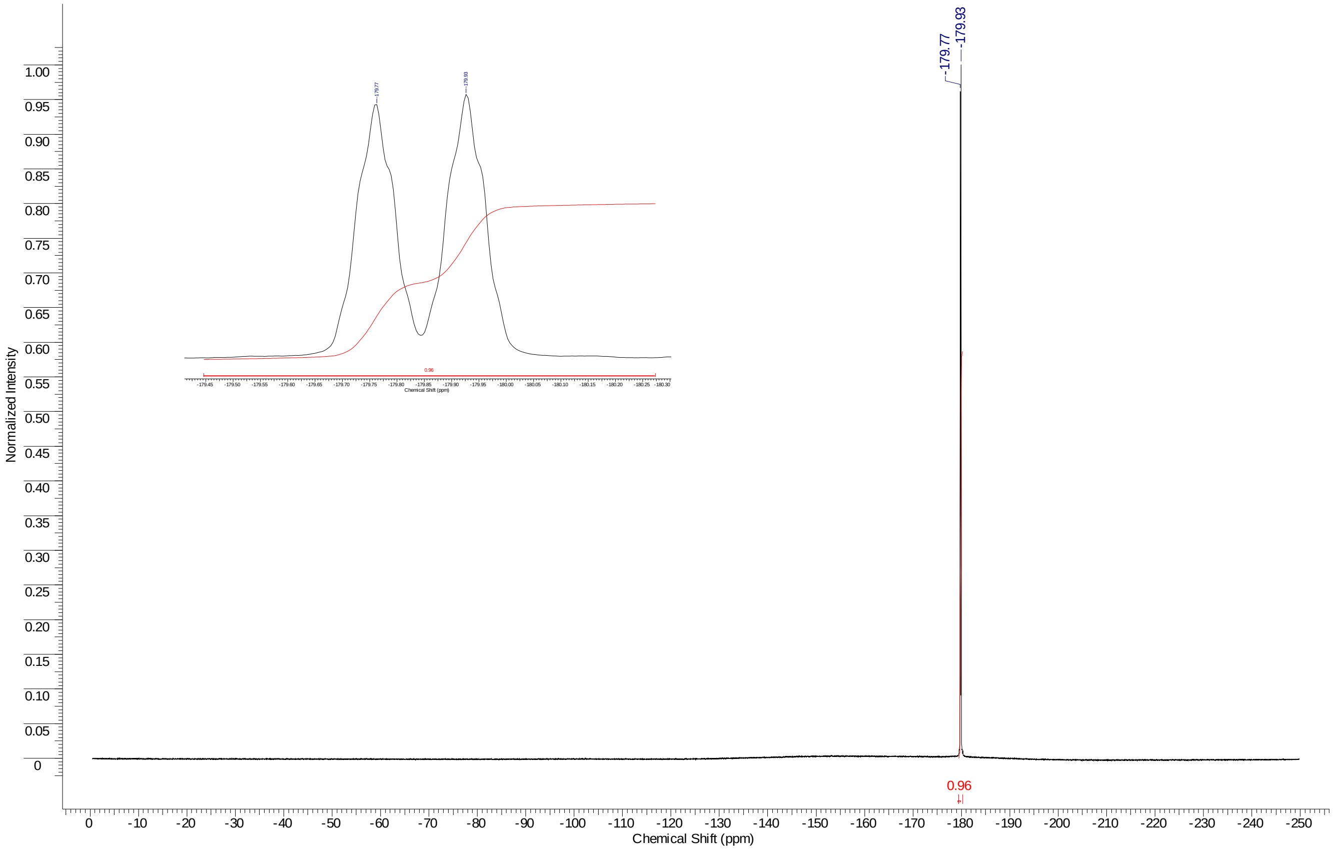

## 4.3 Fluorohydrin 3

## $^{1}$H NMR (400 MHz)

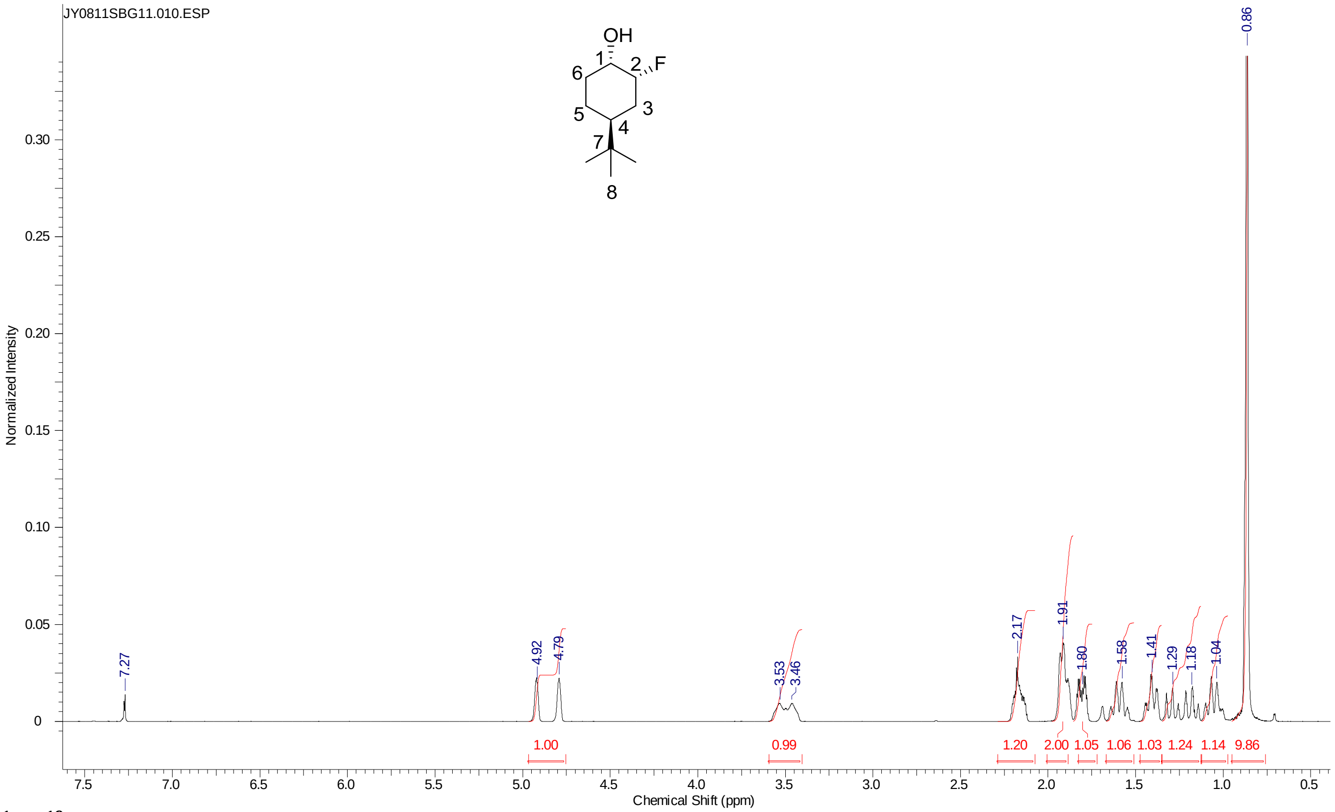


## $^{1}$H{$^{19}$F} NMR (400 MHz)

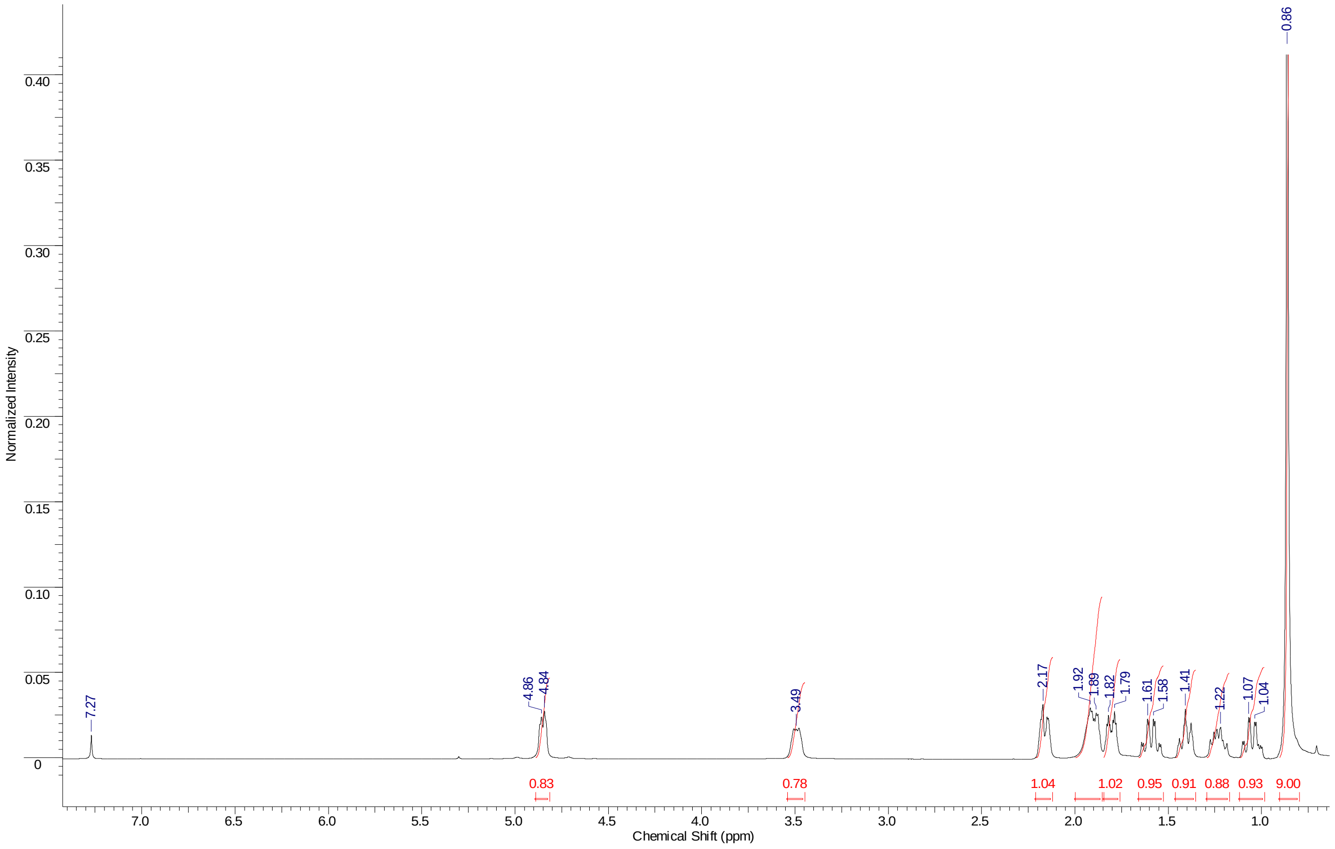

## $^{13}$C NMR (101 MHz)

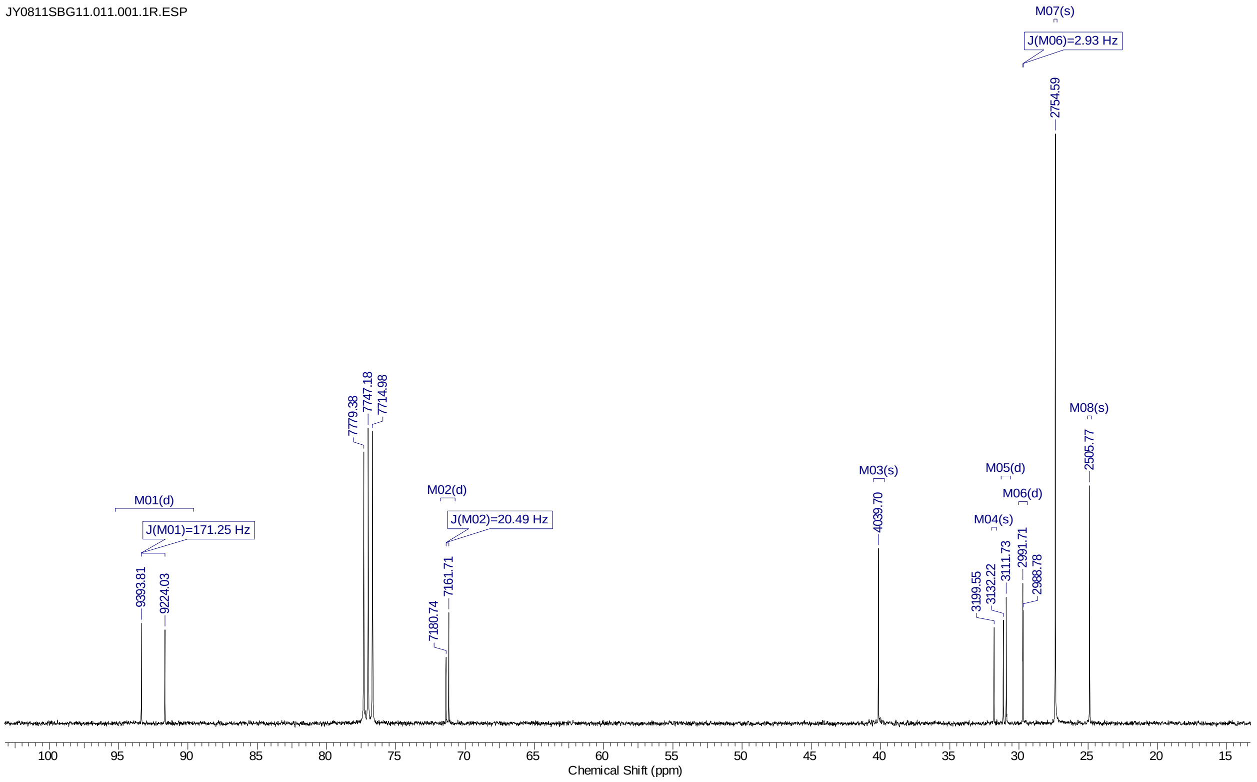


## $^{19}$F NMR (282 MHz)

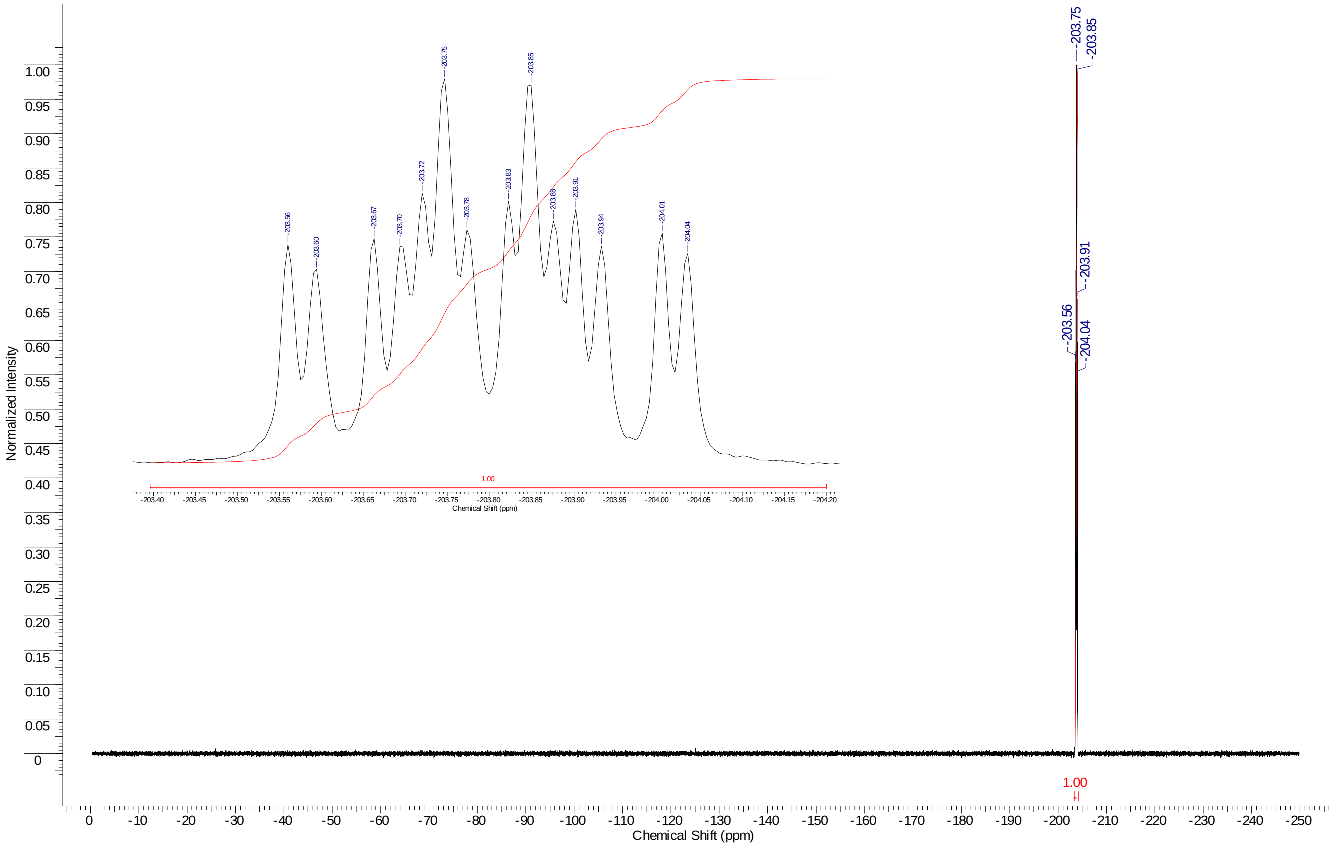

## 4.4 Fluorohydrin 4

### $^1$H NMR (400 MHz)

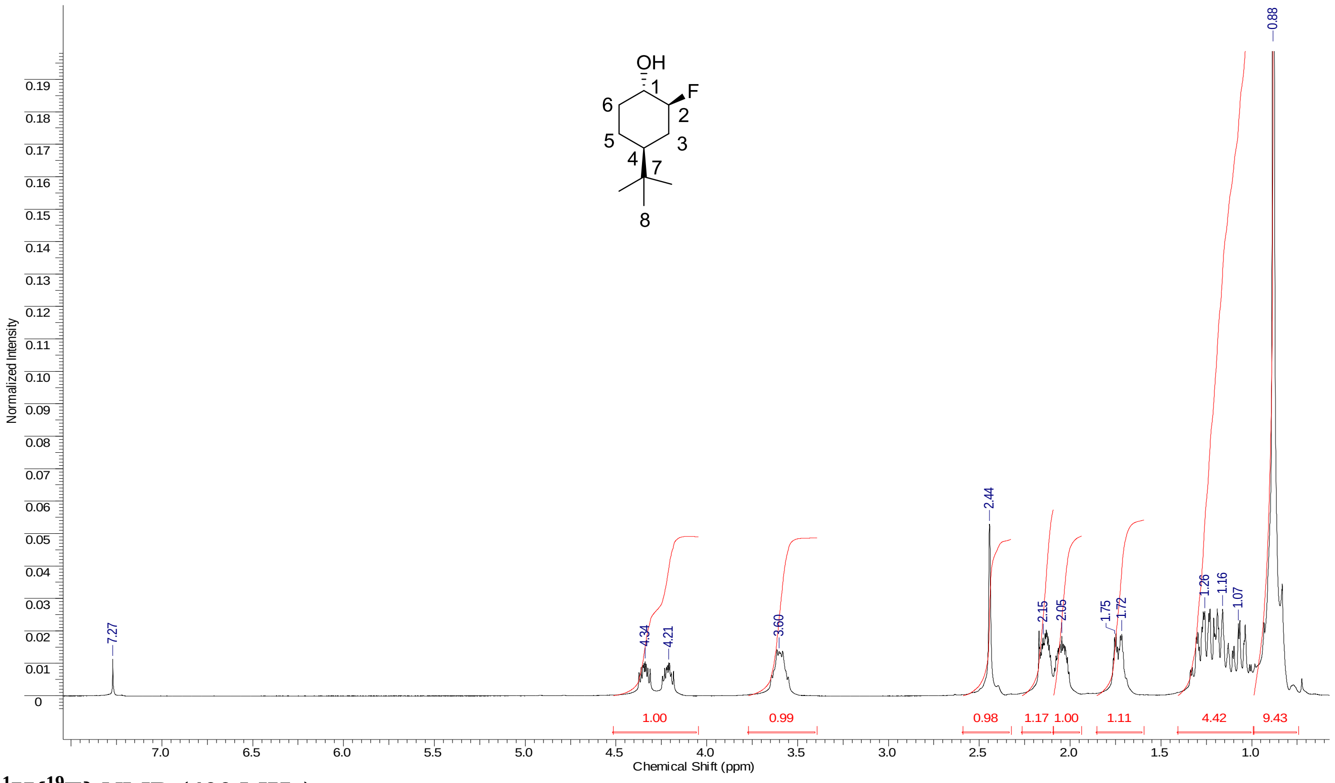


### $^1$H{$^{19}$F} NMR (400 MHz)

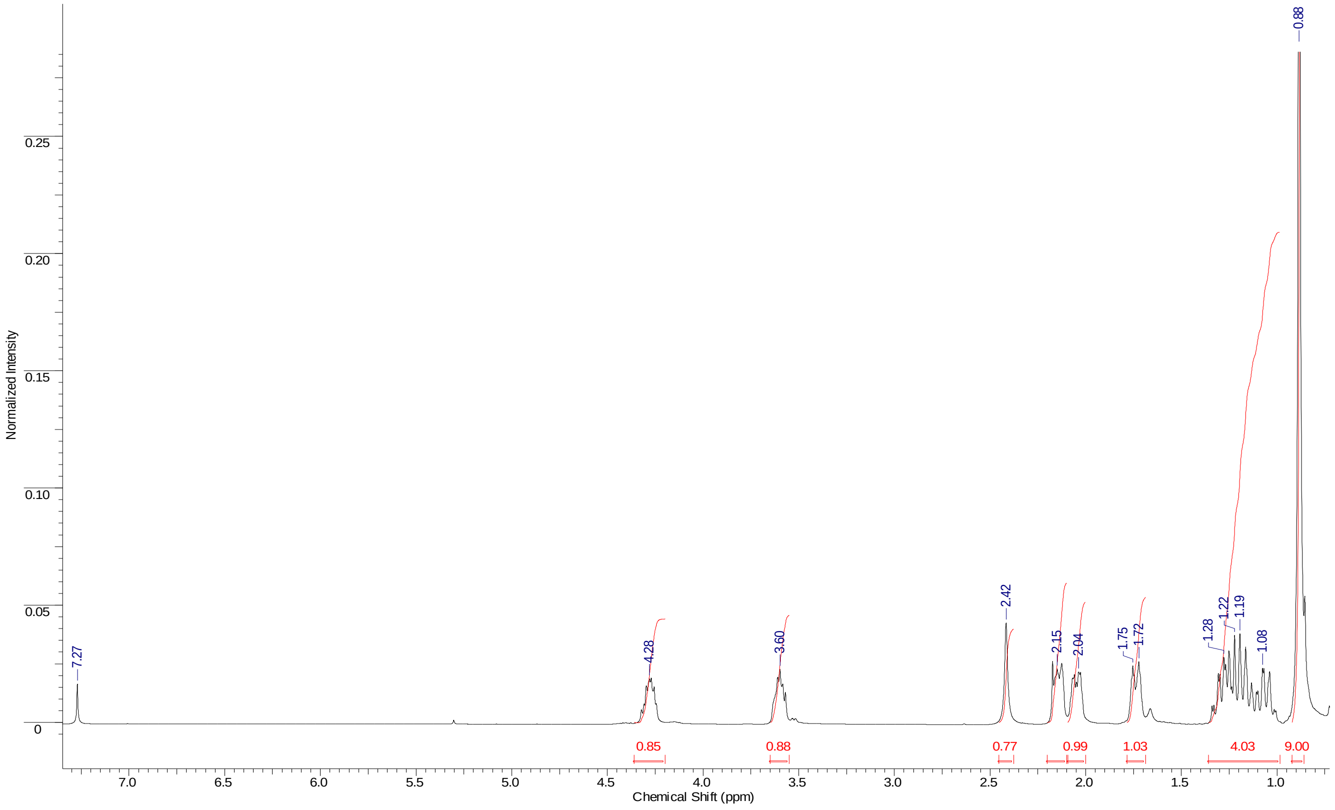

## $^{13}$C NMR (101 MHz)

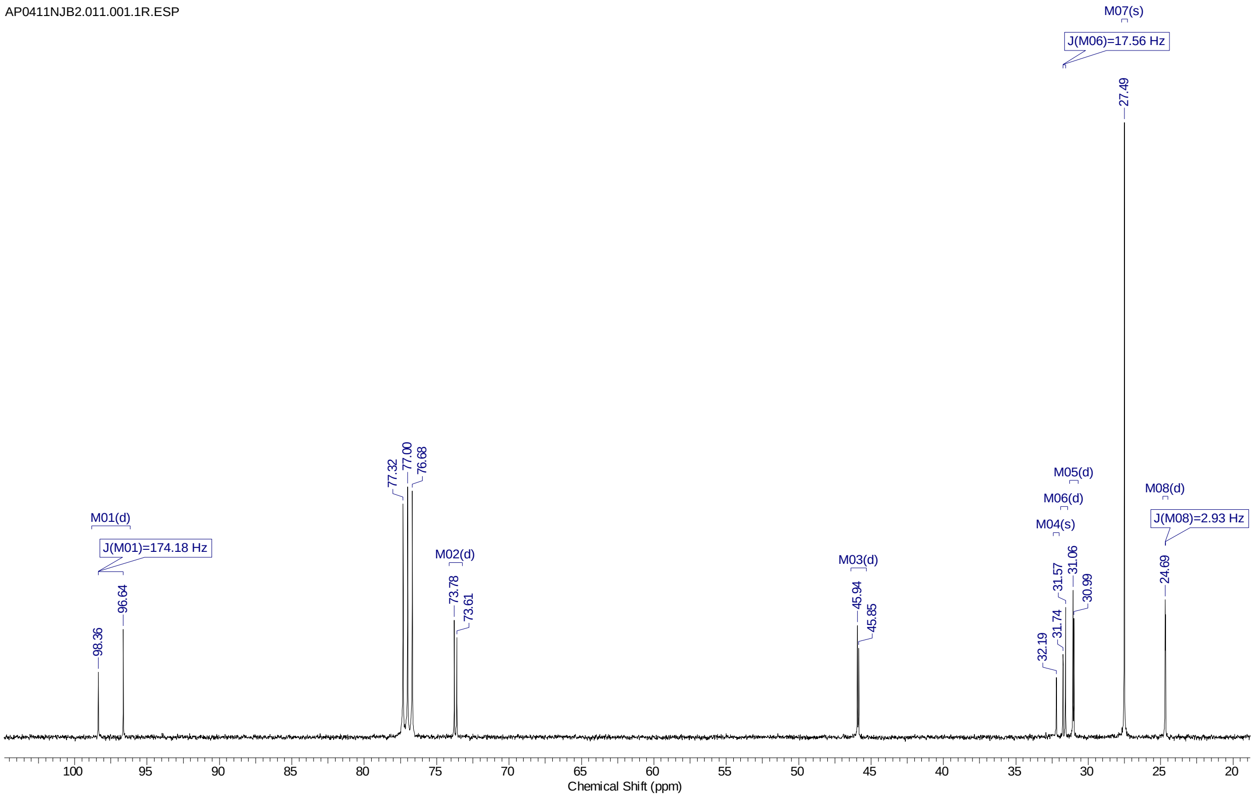


## $^{19}$F NMR (282 MHz)

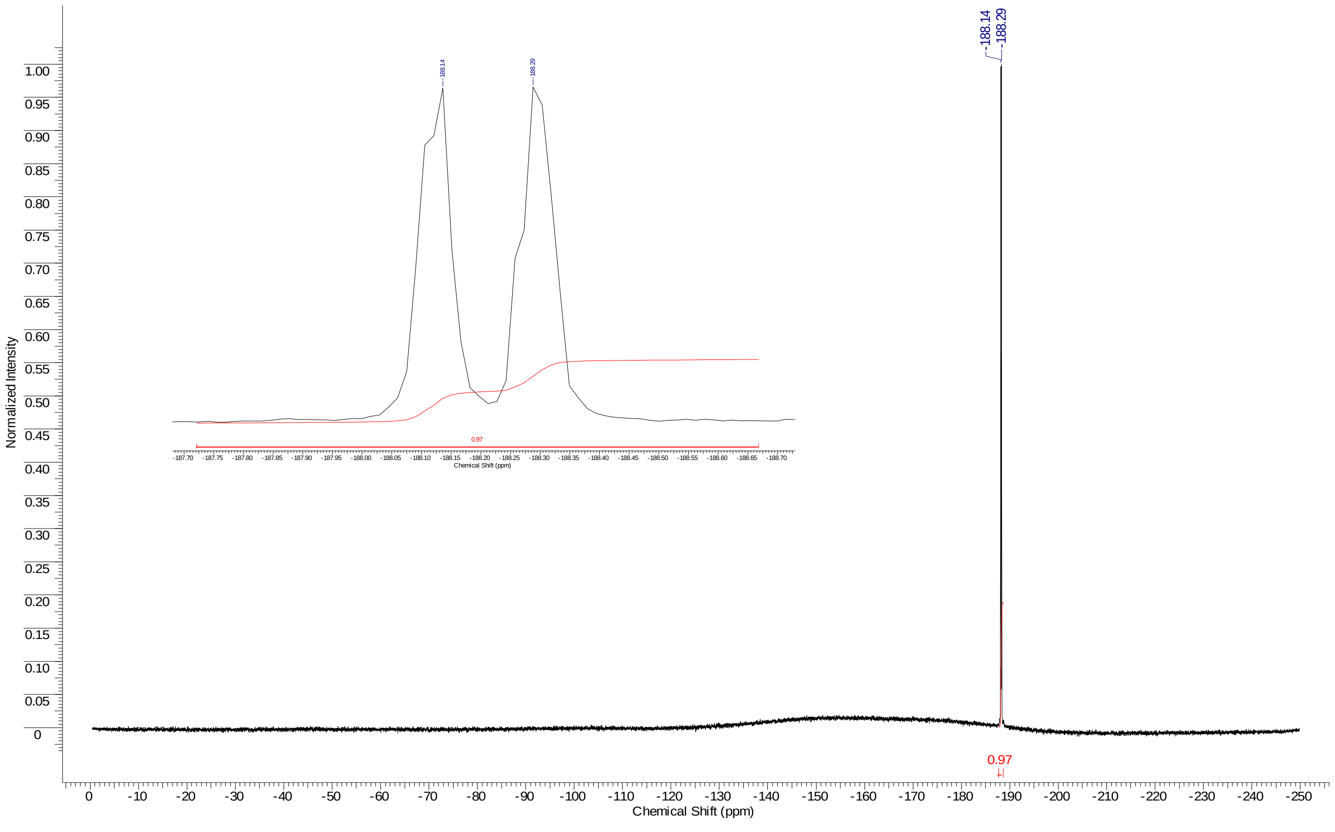

### 4.5 Fluorohydrin 5

## $^{1}$H NMR (400 MHz)

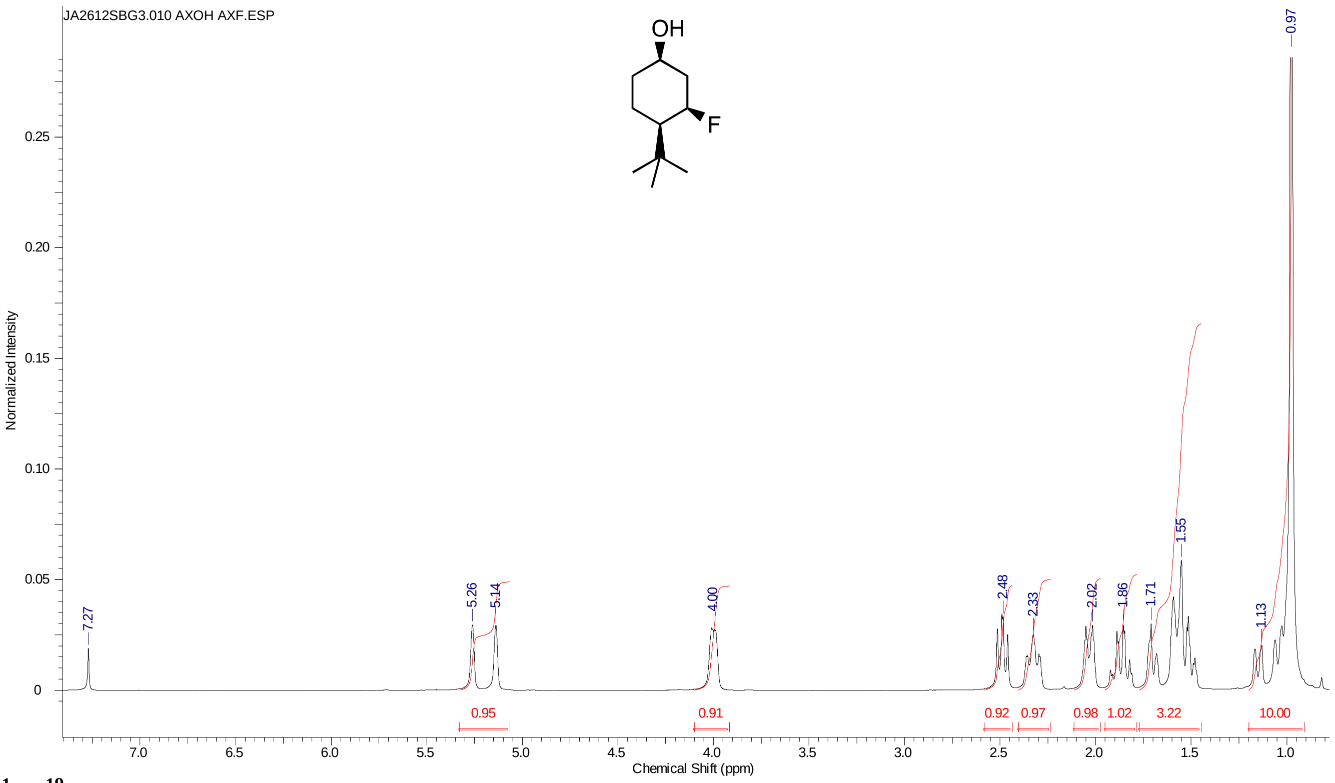


## $^{1}$H{$^{19}$F} NMR (400 MHz)

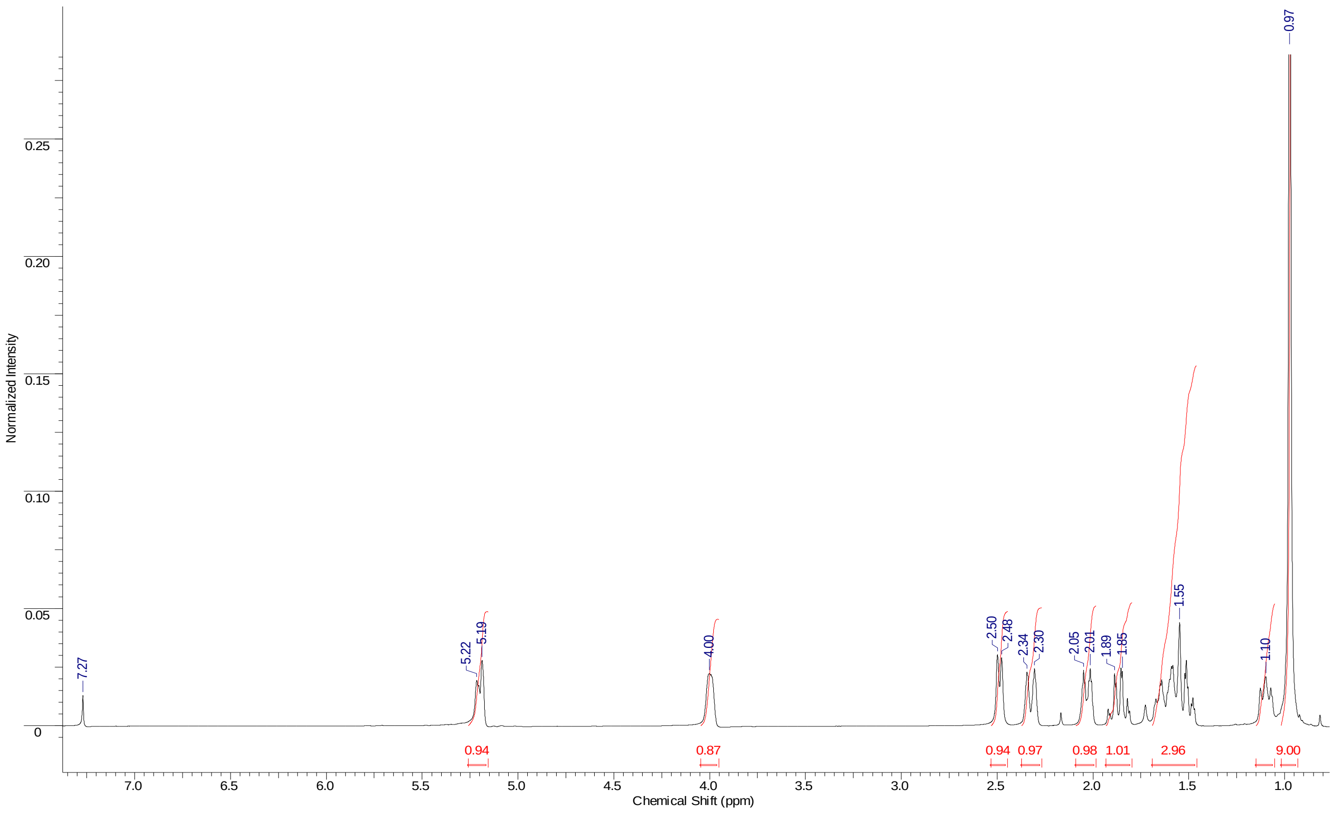

## $^{13}$C NMR (101 MHz)

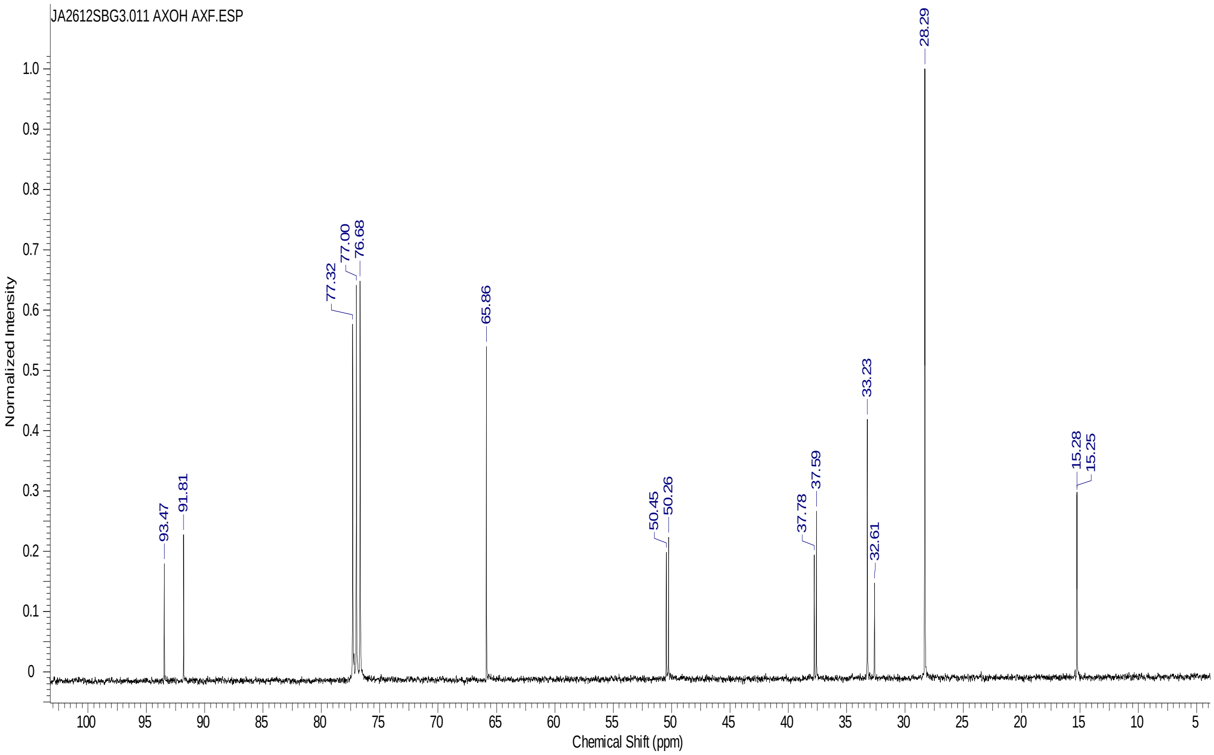


## $^{19}$F NMR (282 MHz)

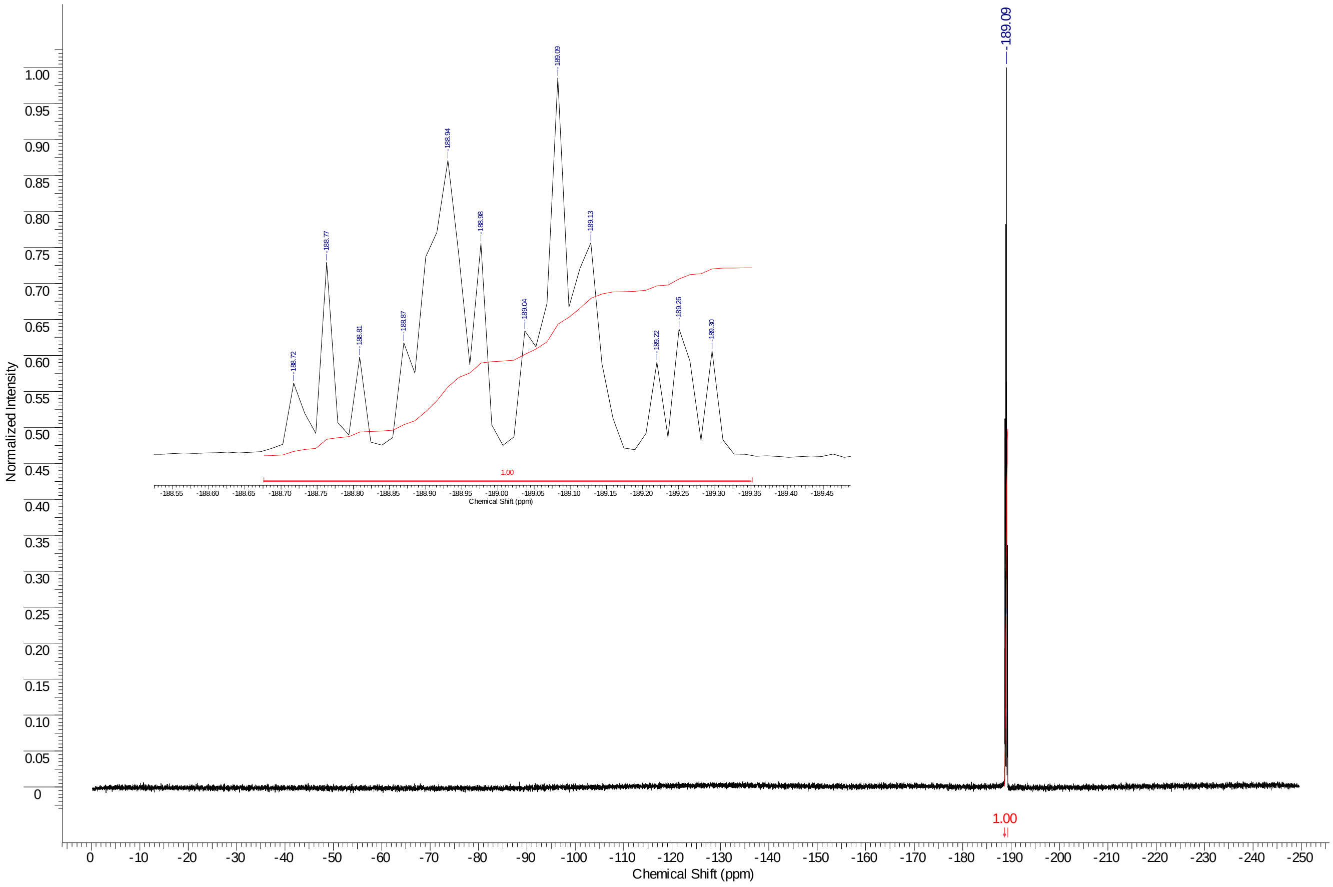

## 4.6 Fluorohydrin 6

### $^{1}$H NMR (400 MHz)

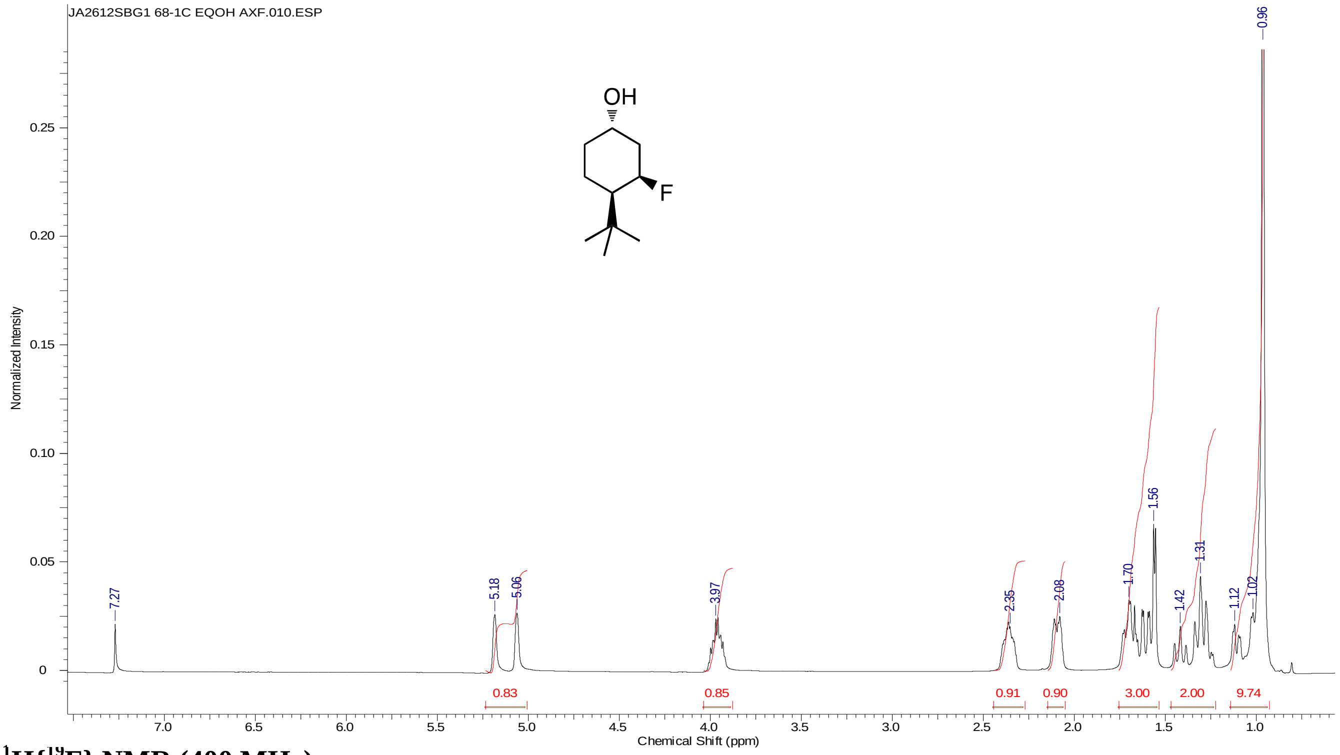


### $^{1}$H{$^{19}$F} NMR (400 MHz)

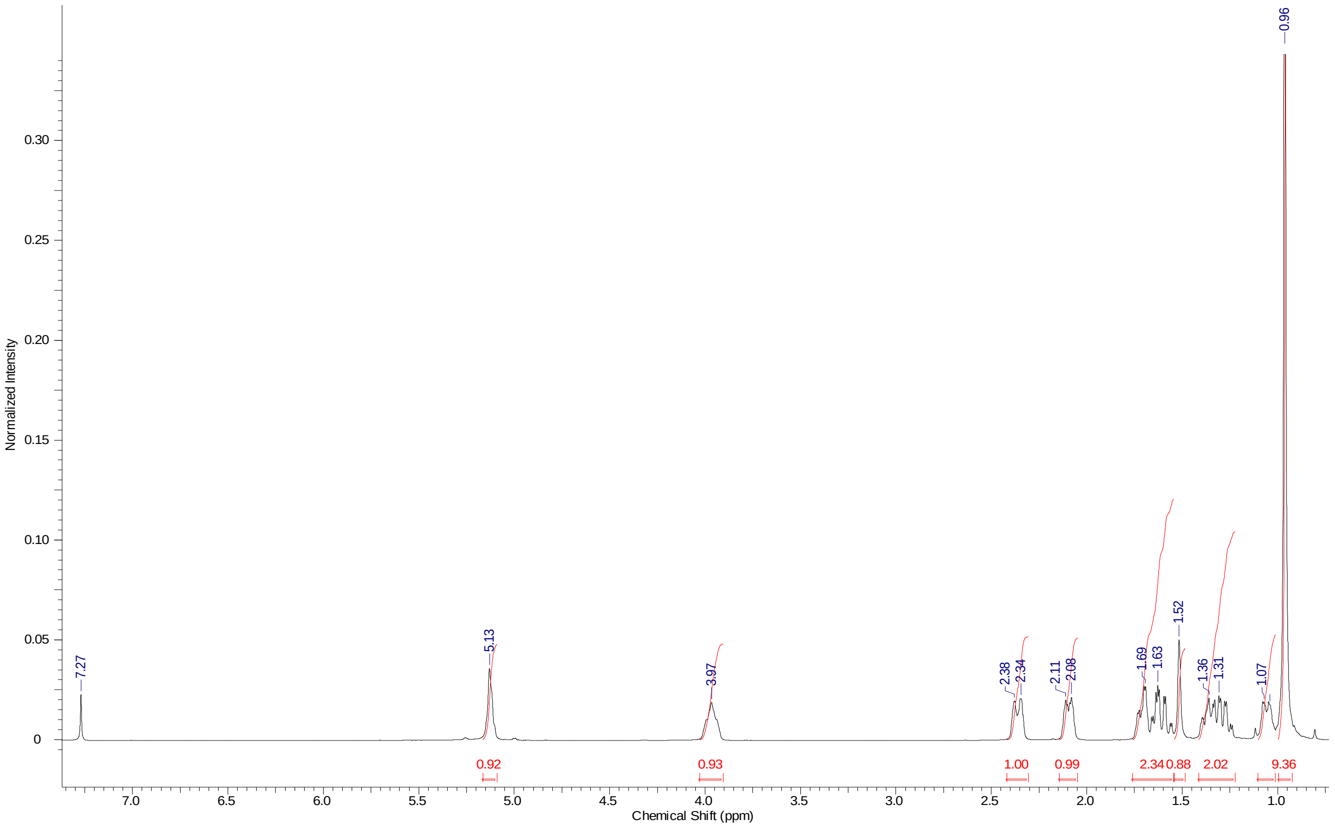

$^{13}$C NMR (101 MHz)

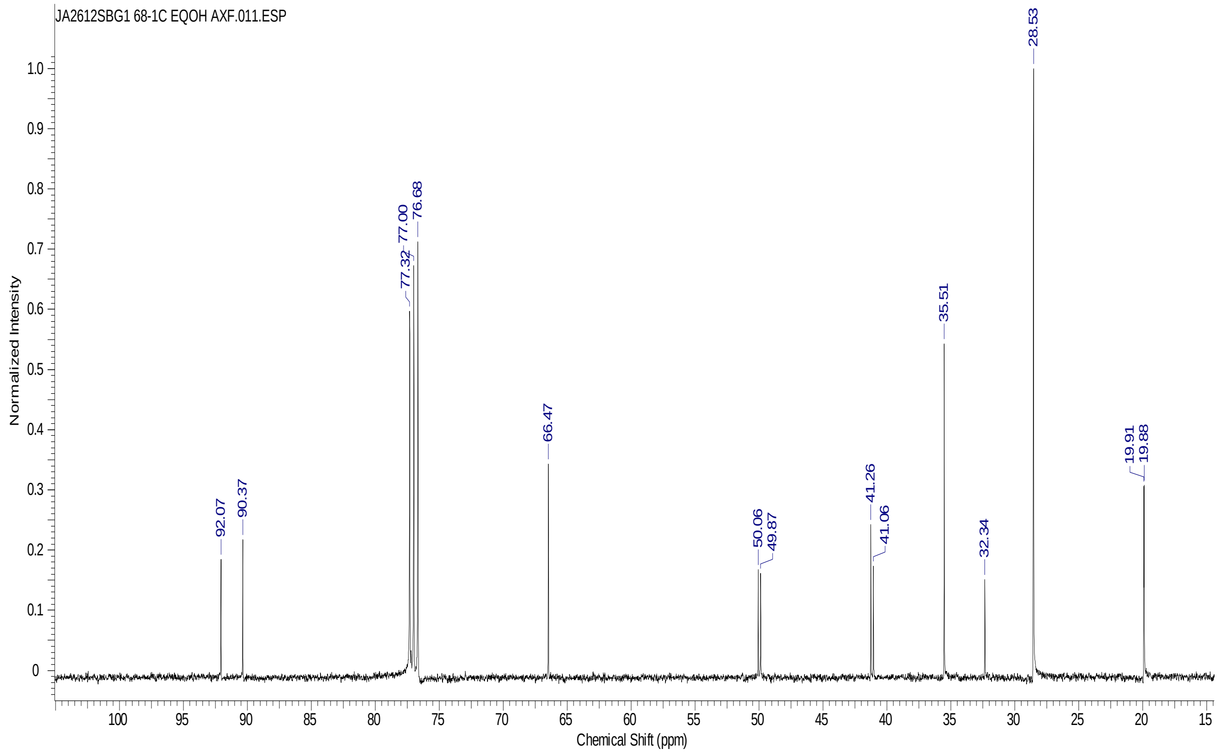


$^{19}$F NMR (282 MHz)

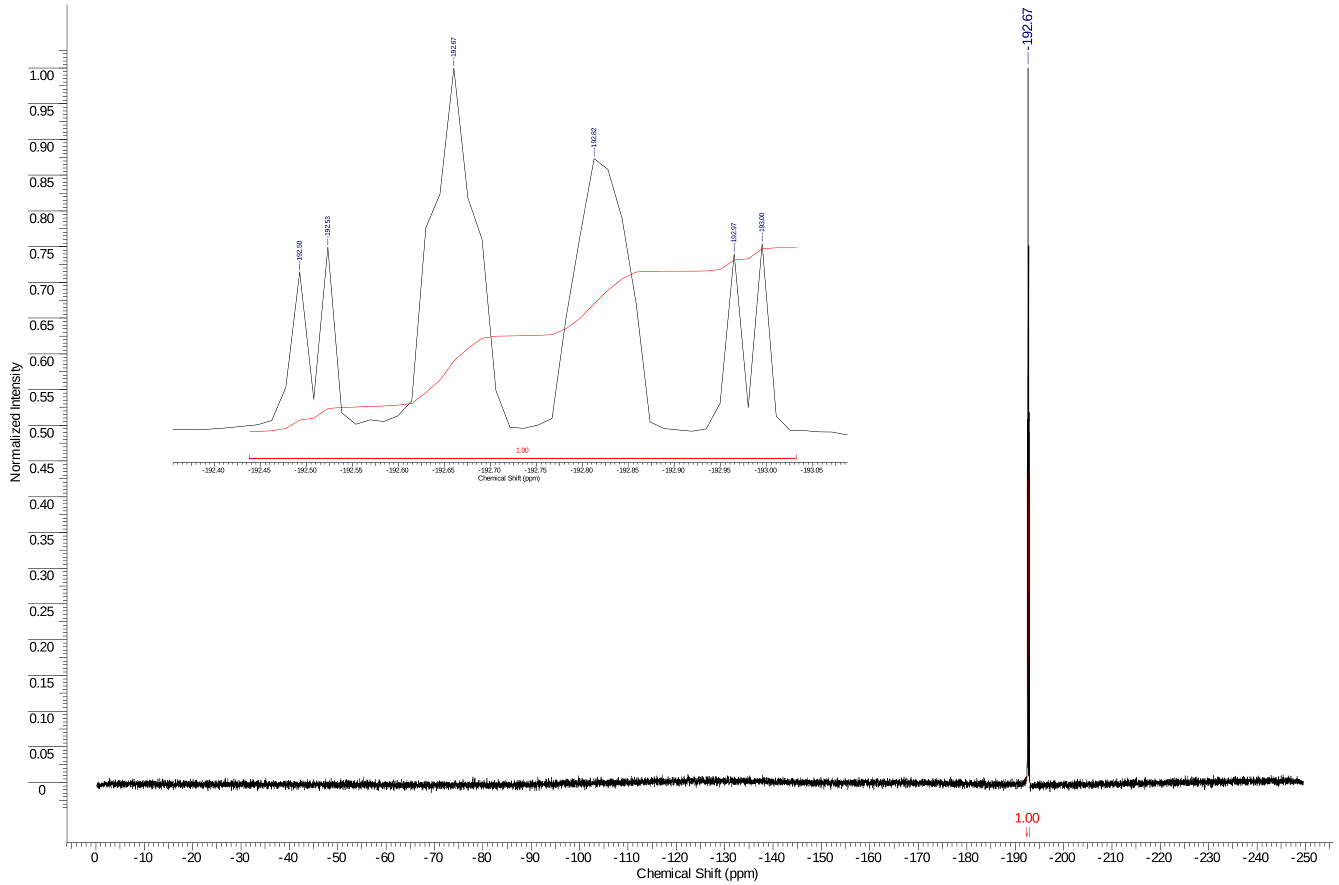

## 4.7 Difluorohydrin 7

### $^{1}$H NMR (400 MHz)

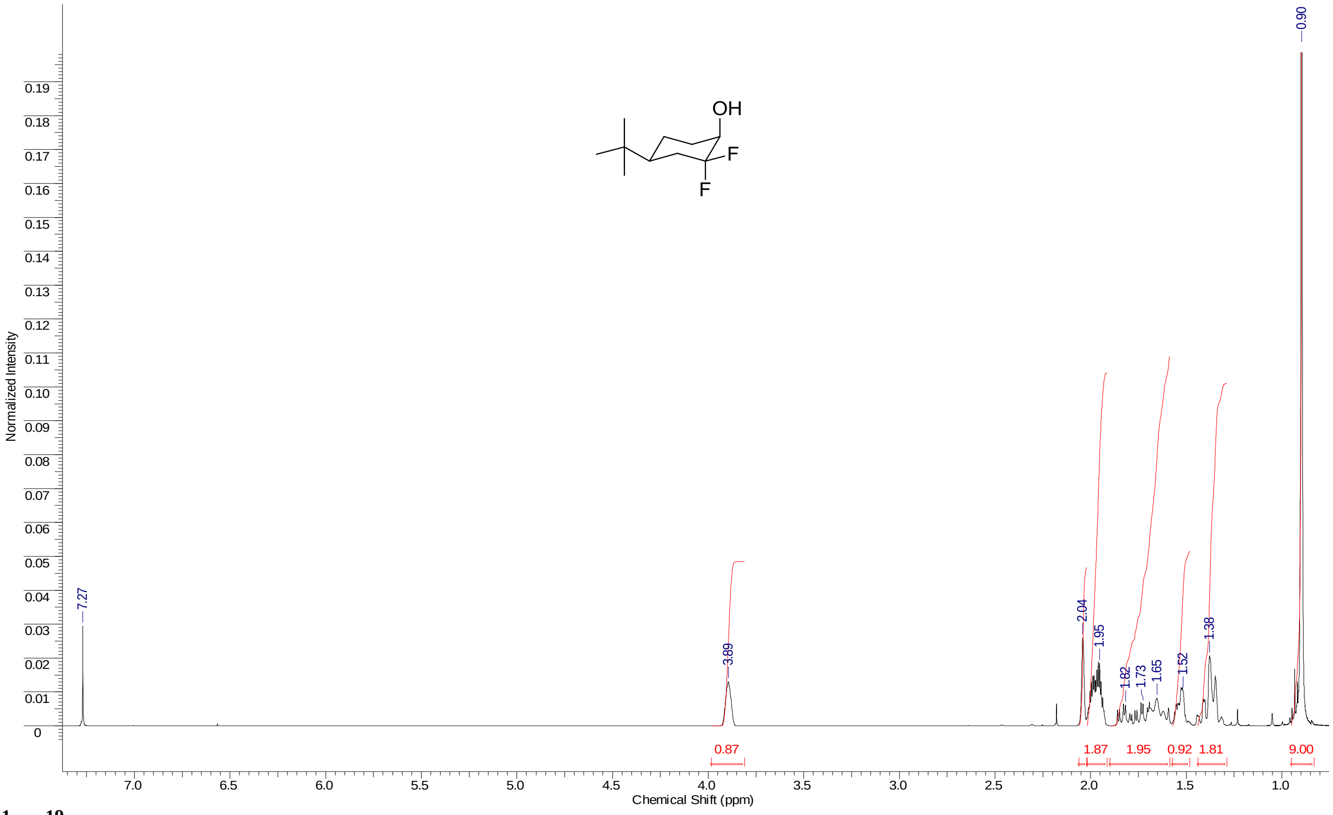


### $^{1}$H{$^{19}$F} NMR (400 MHz)

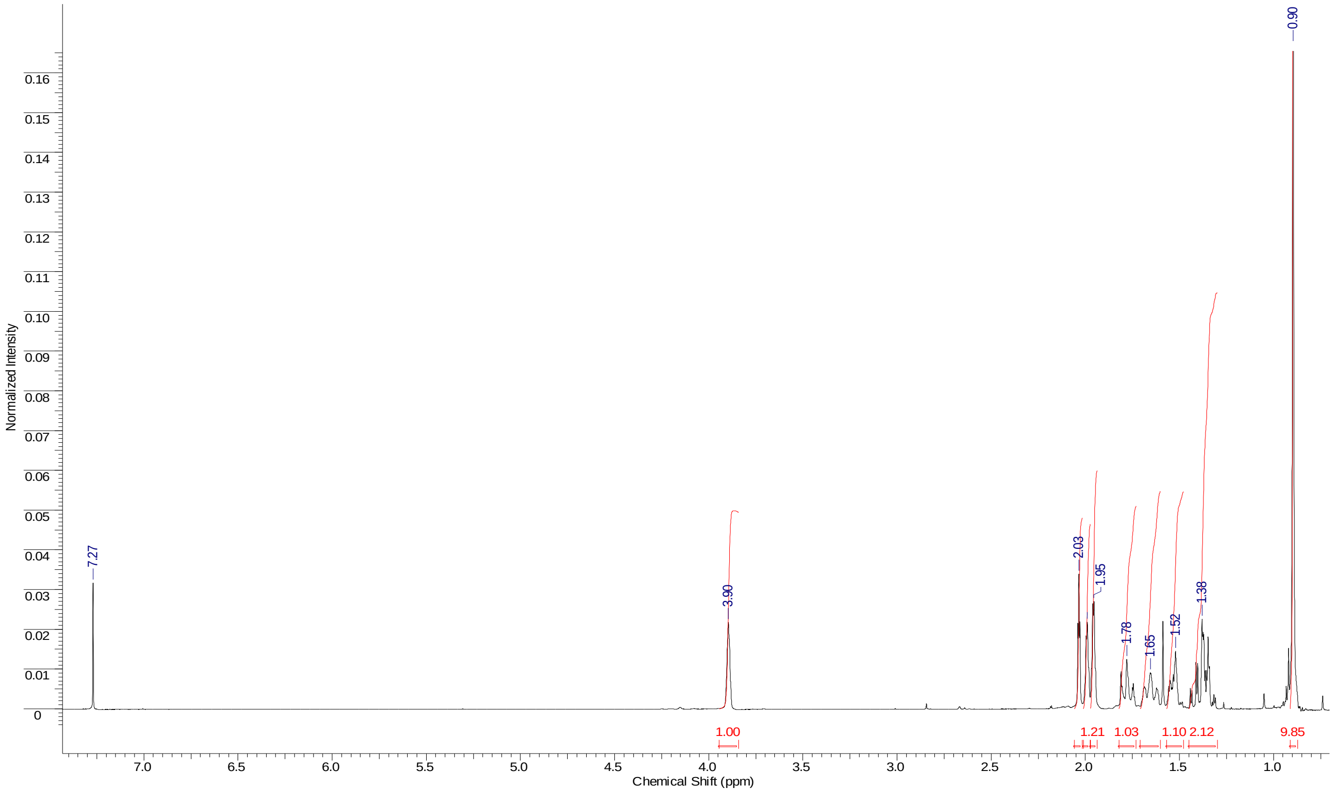

## $^{13}C$ NMR (101 MHz)

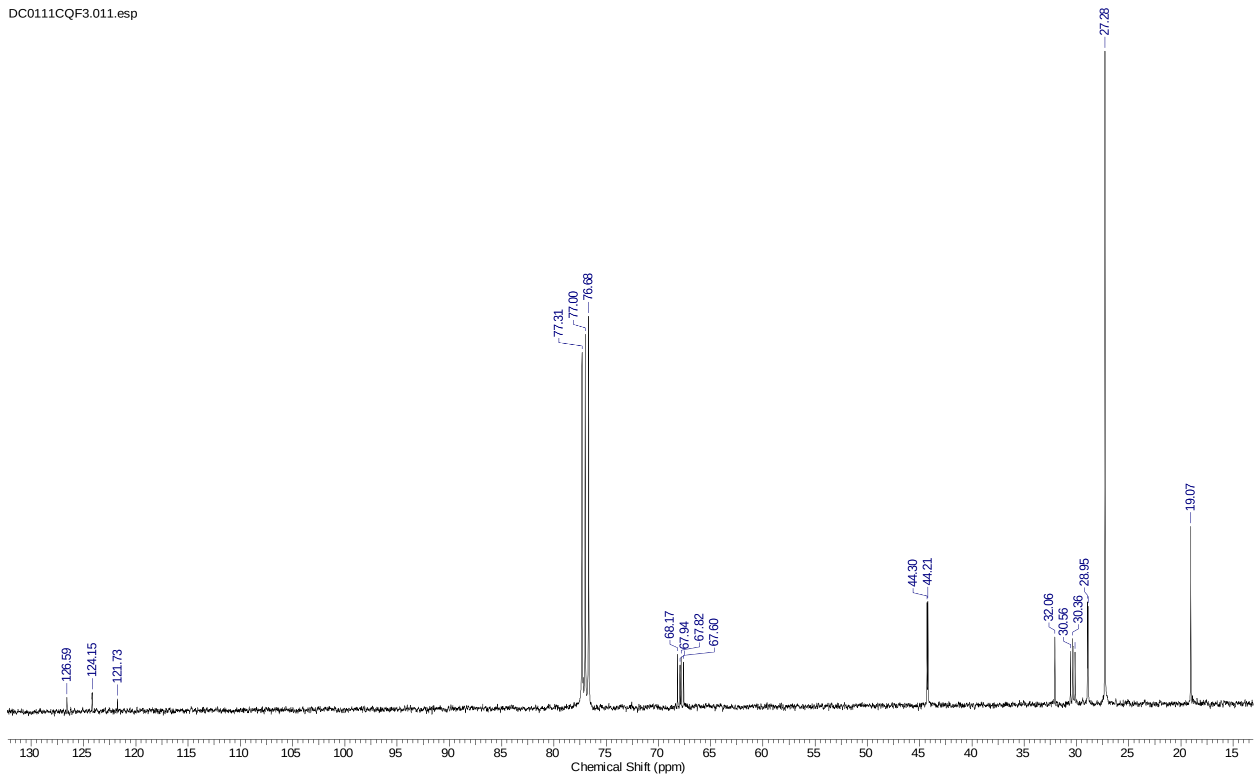


## $^{19}F$ NMR (282 MHz)

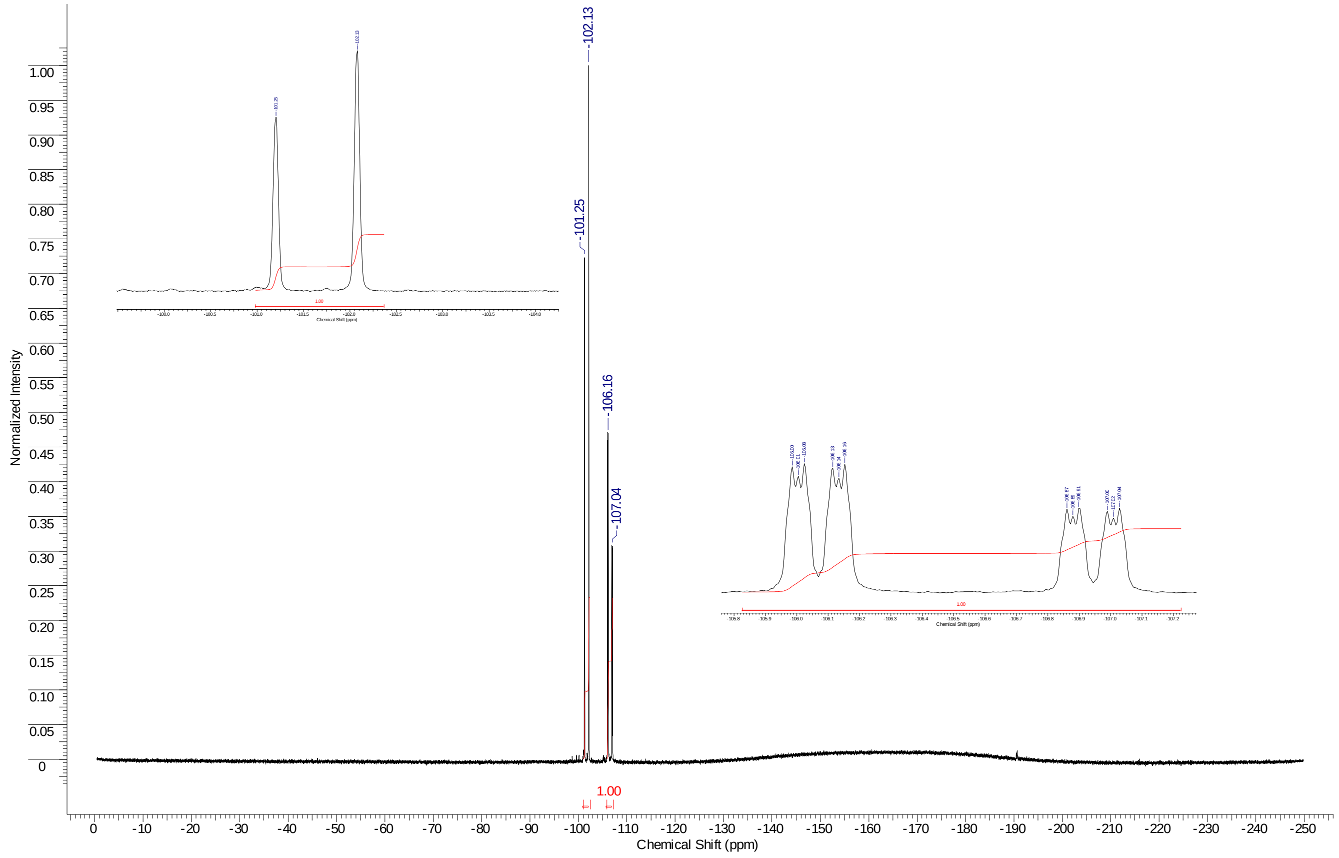

## 4.8 Difluorohydrin 8

### ¹H NMR (400 MHz)

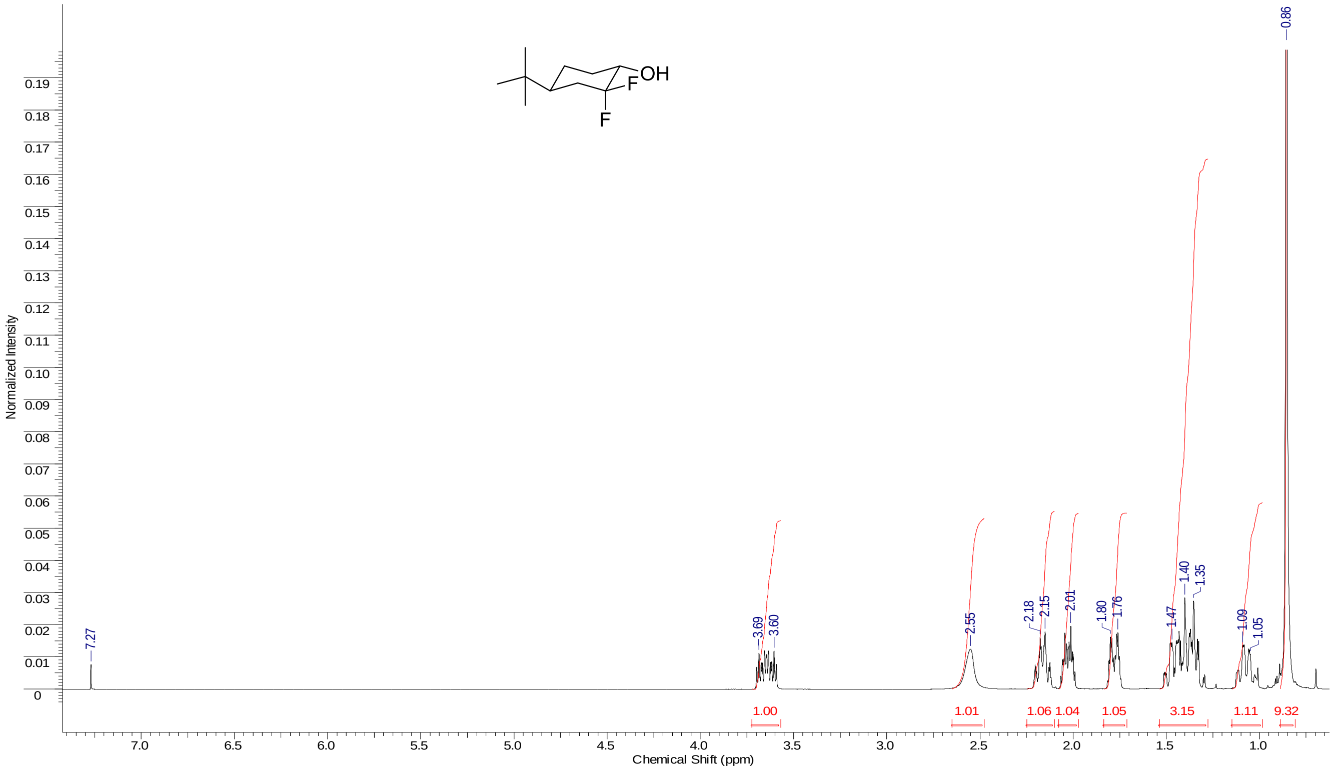


### ¹H{¹⁹F} NMR (400 MHz)

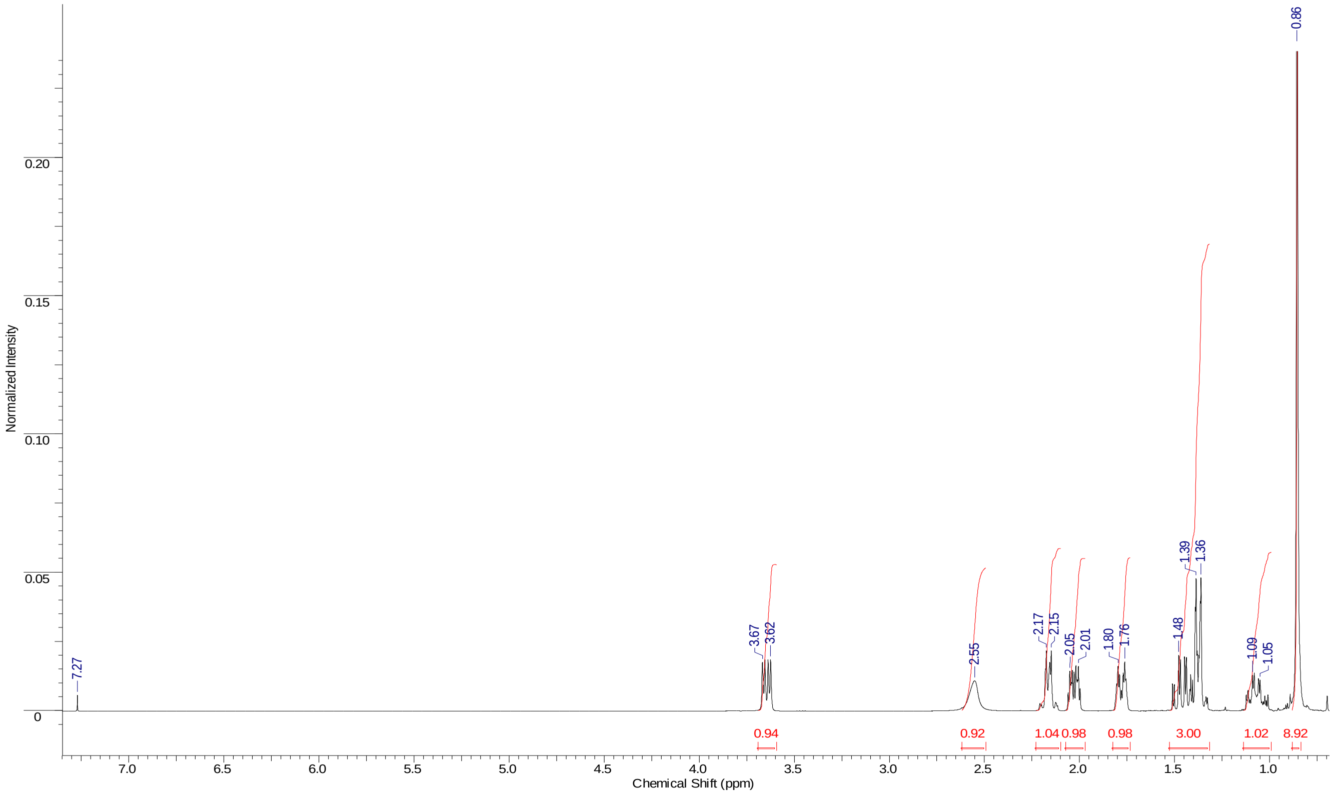

$^{13}$C NMR (101 MHz)

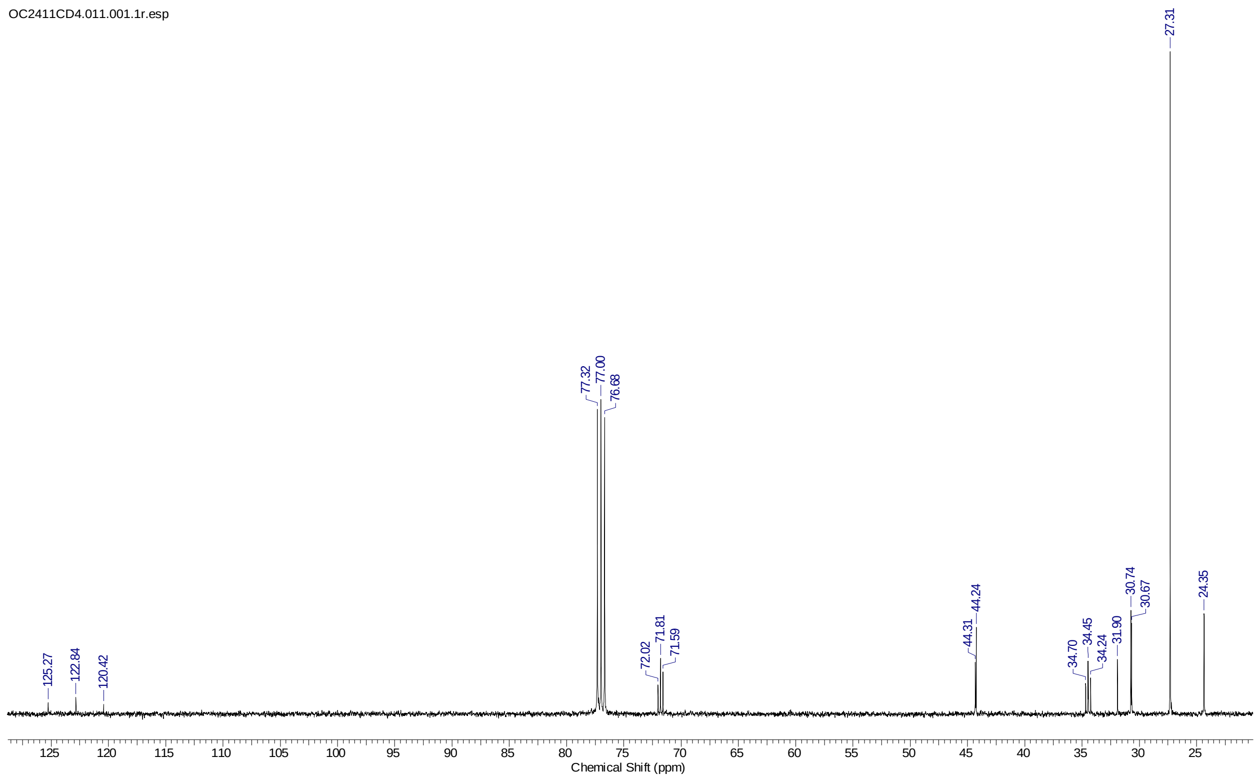


$^{19}$F NMR (282 MHz)

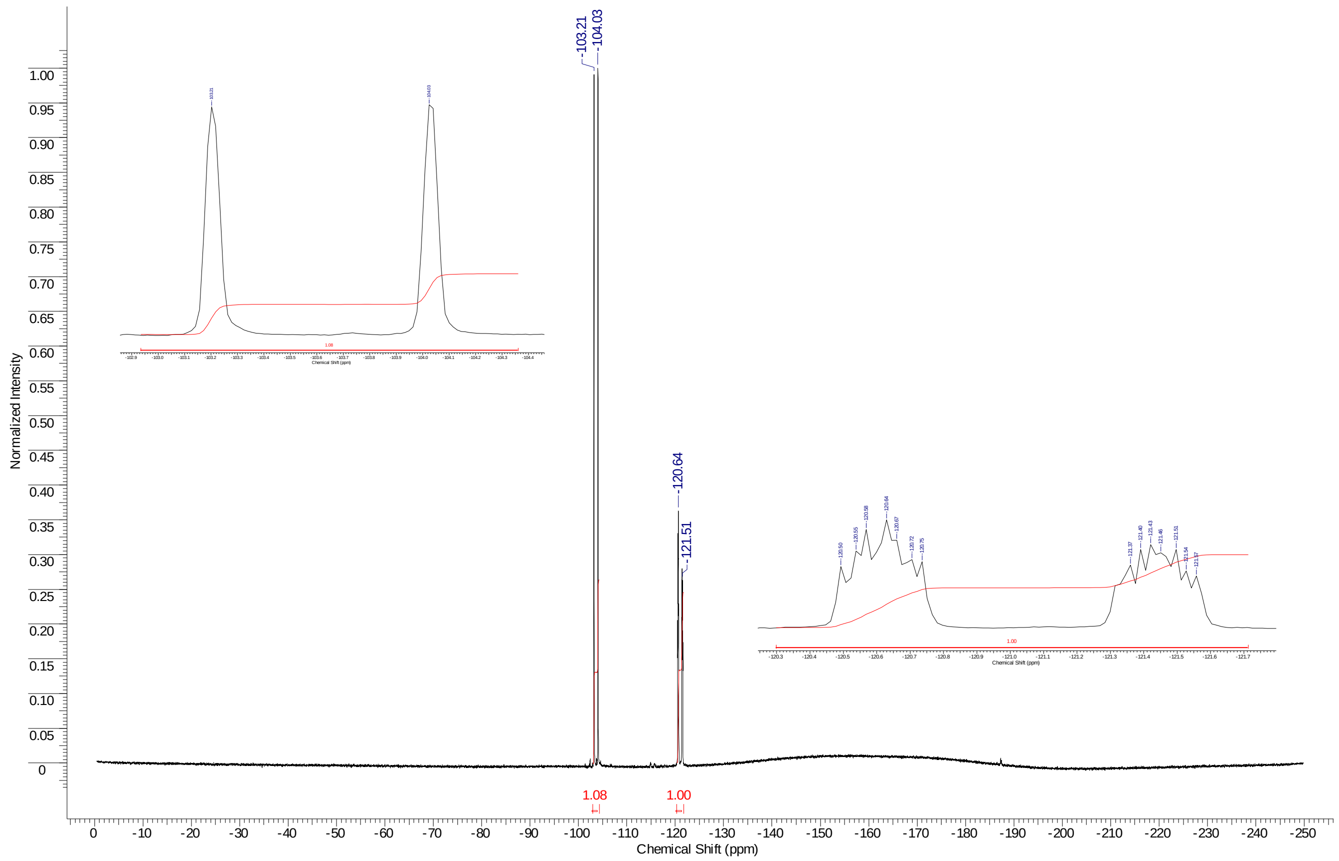

## 4.9 Allylic alcohol SI1

### $^{1}$H NMR (400 MHz)

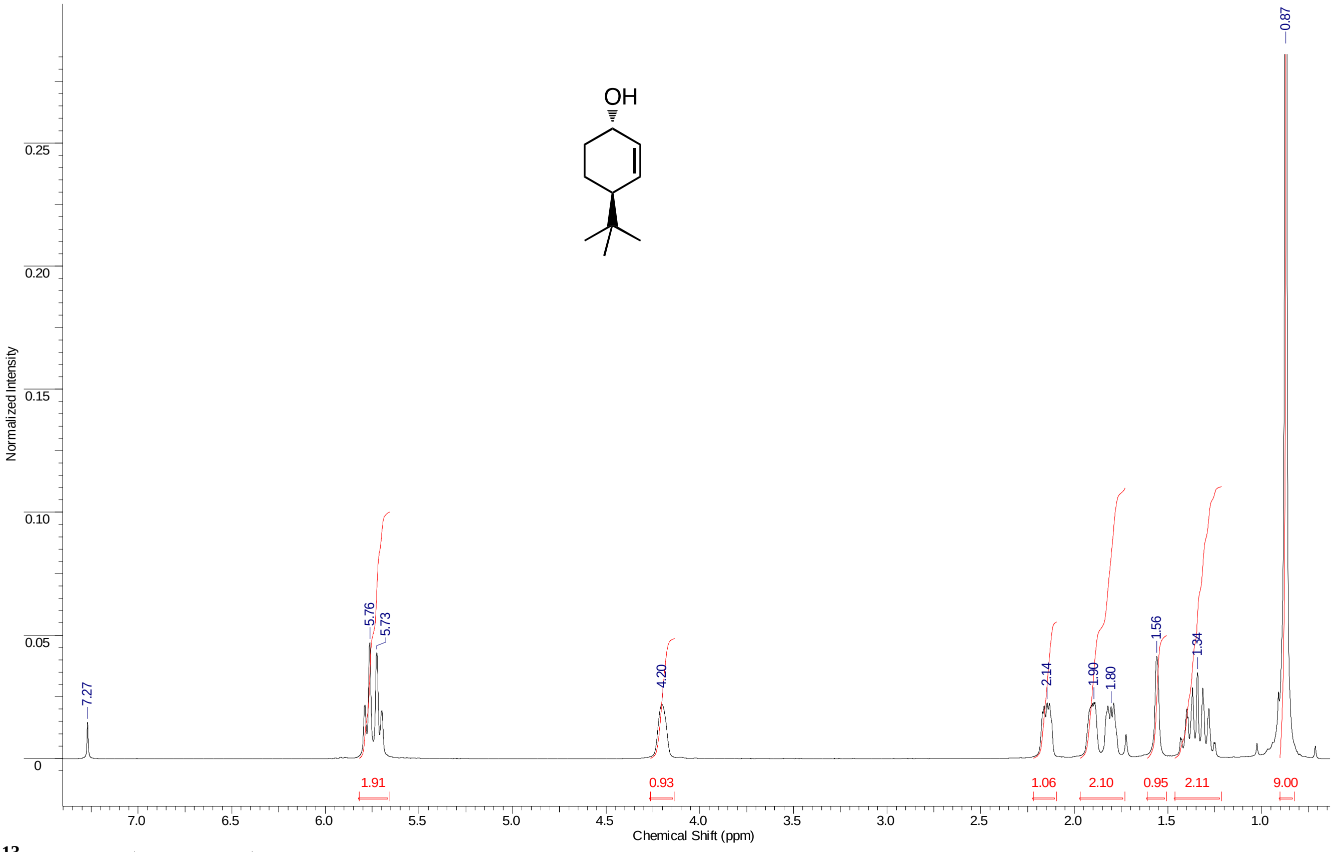


### $^{13}$C NMR (101 MHz)

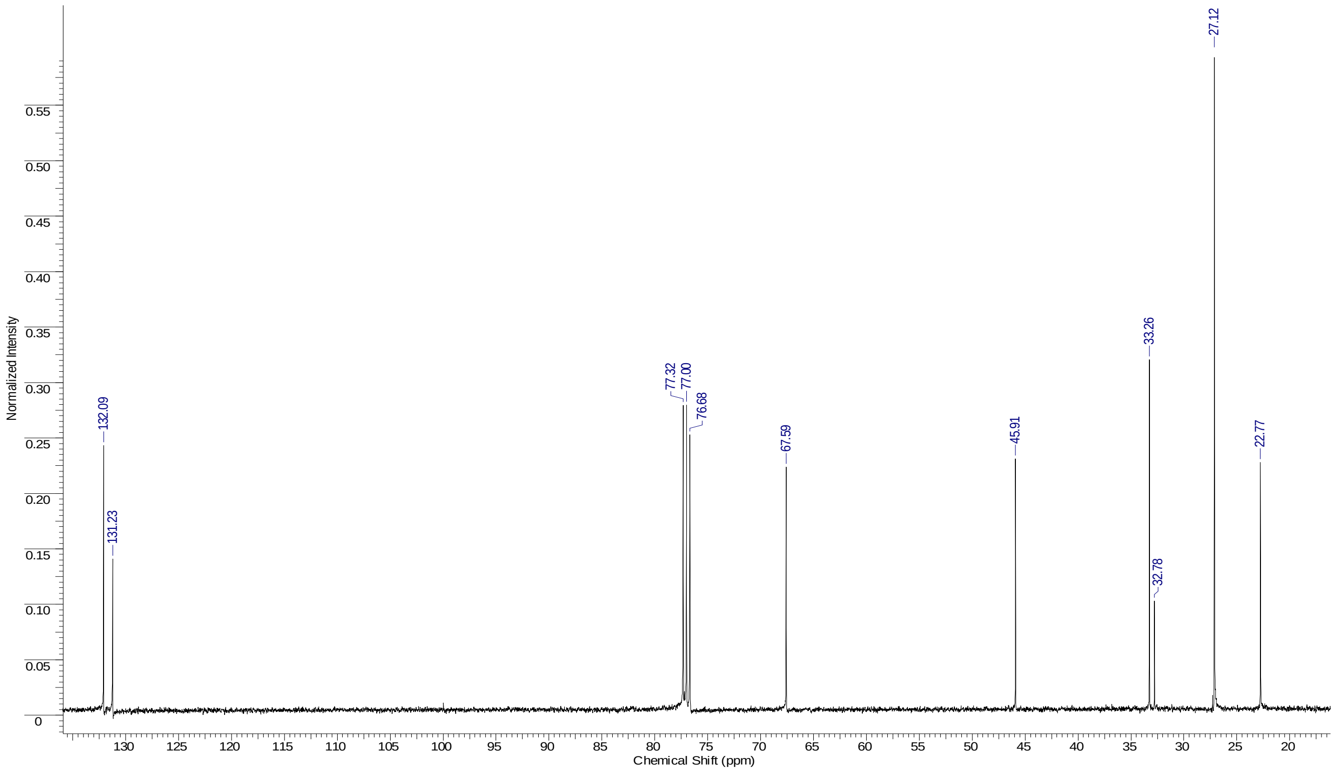

## 4.10 Iodofluorination product SI2

### $^1$H NMR (400 MHz)

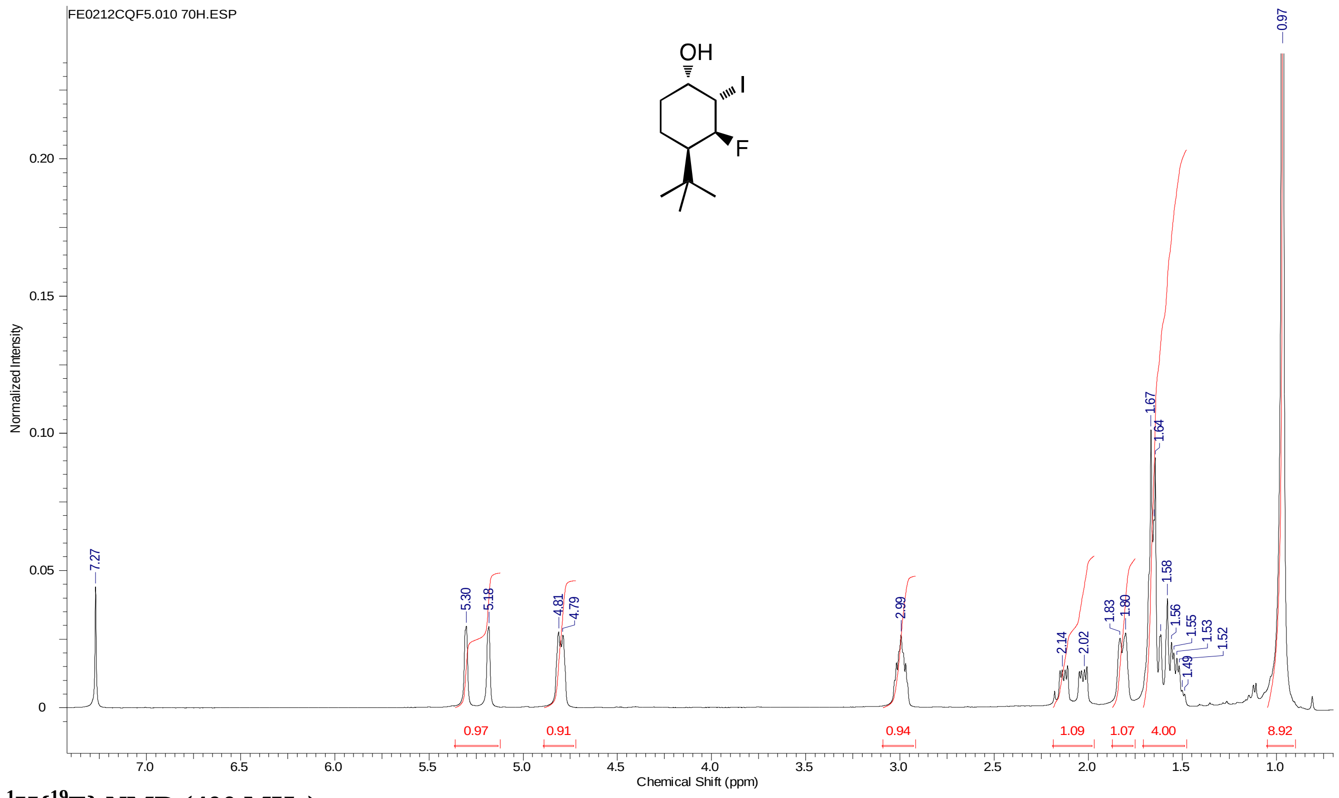


### $^1$H{$^{19}$F} NMR (400 MHz)

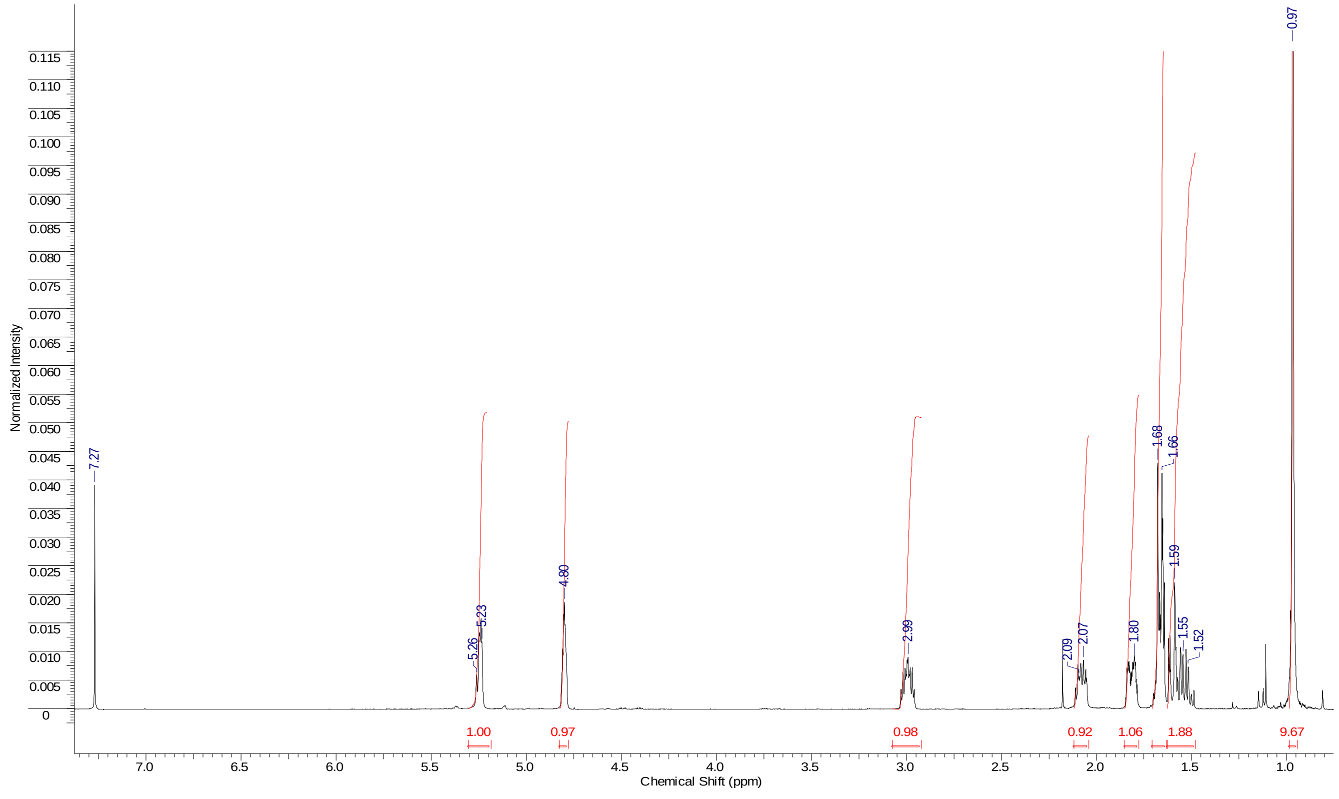

## $^{13}$C NMR (101 MHz)

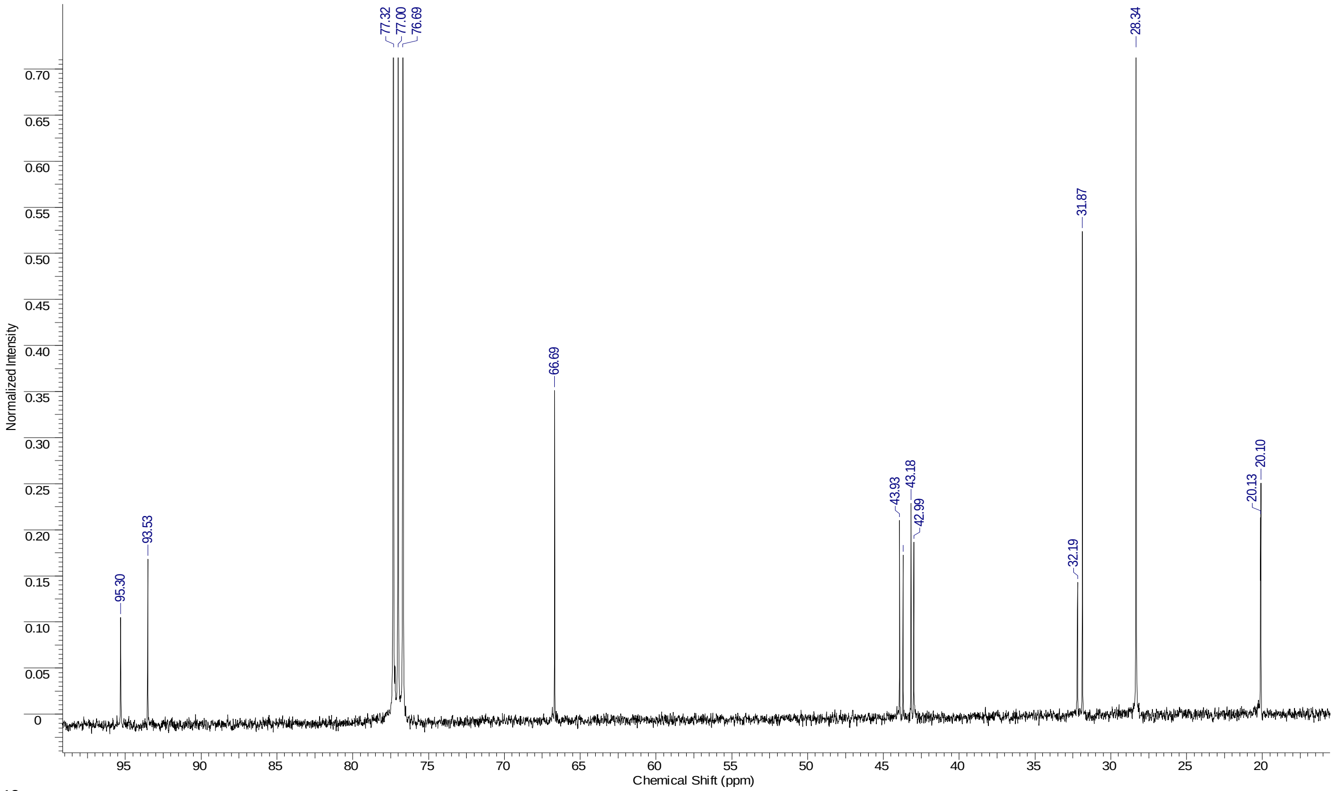


## $^{19}$F NMR (282 MHz)

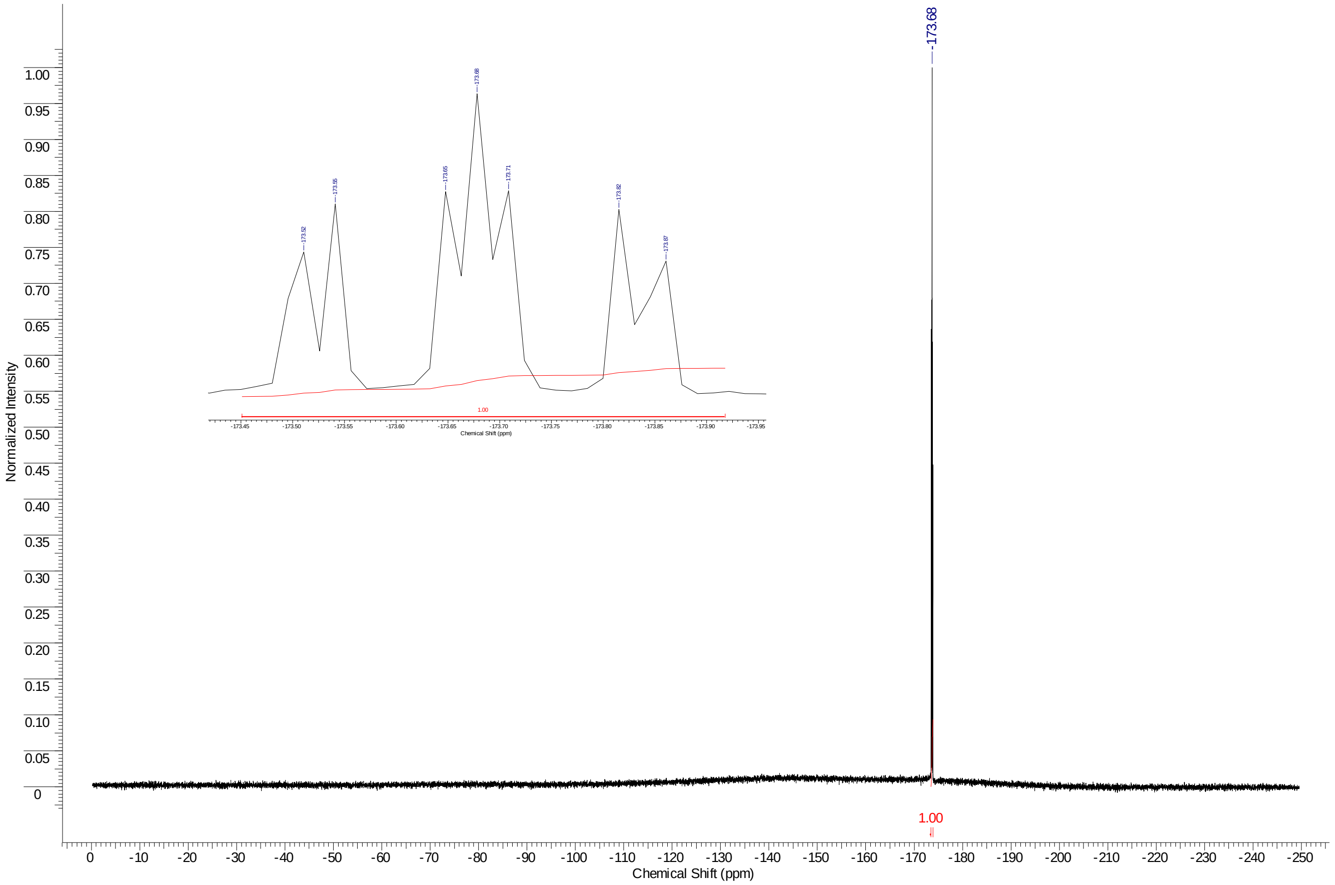

### 4.11 Mitsunobu product SI3

## $^{1}$H NMR (400 MHz)

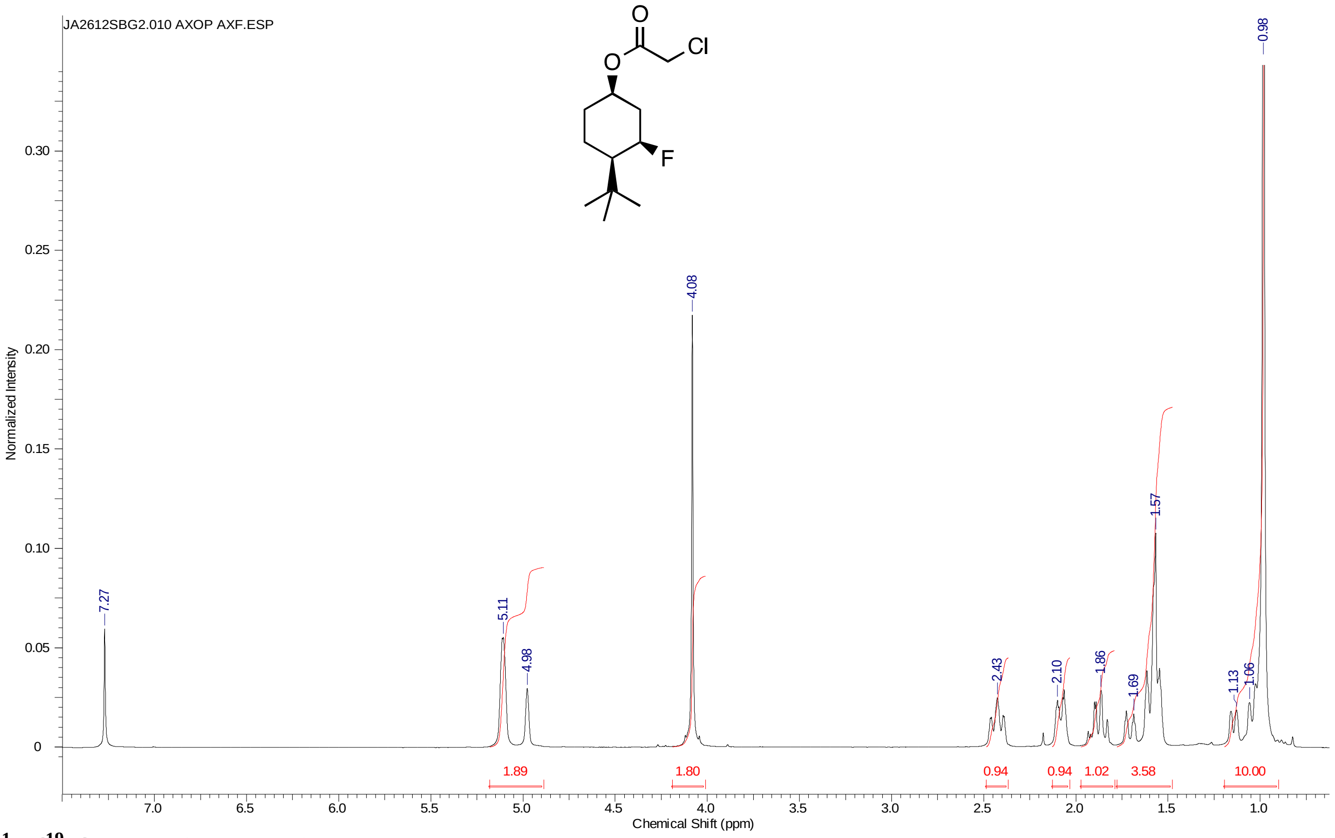


## $^{1}$H{$^{19}$F} NMR (400 MHz)

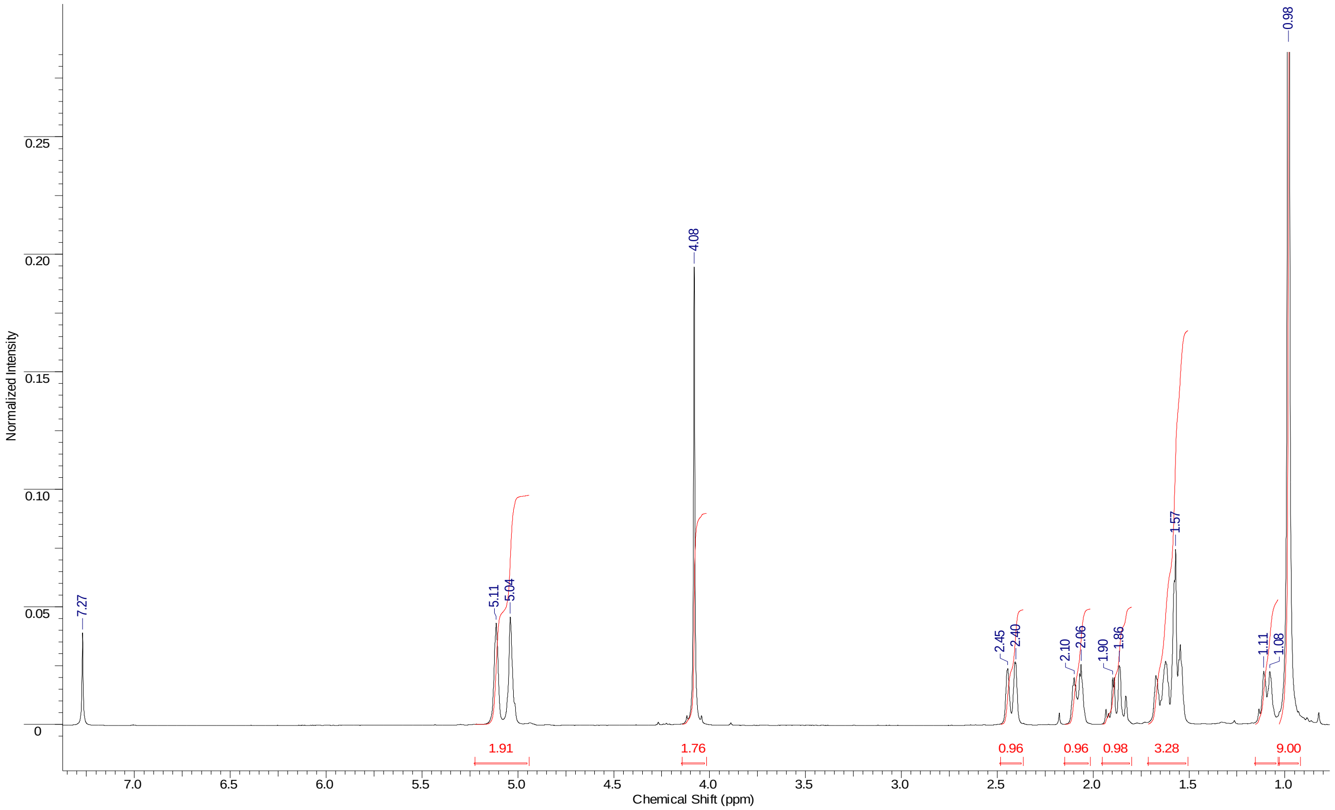

## $^{13}$C NMR (101 MHz)

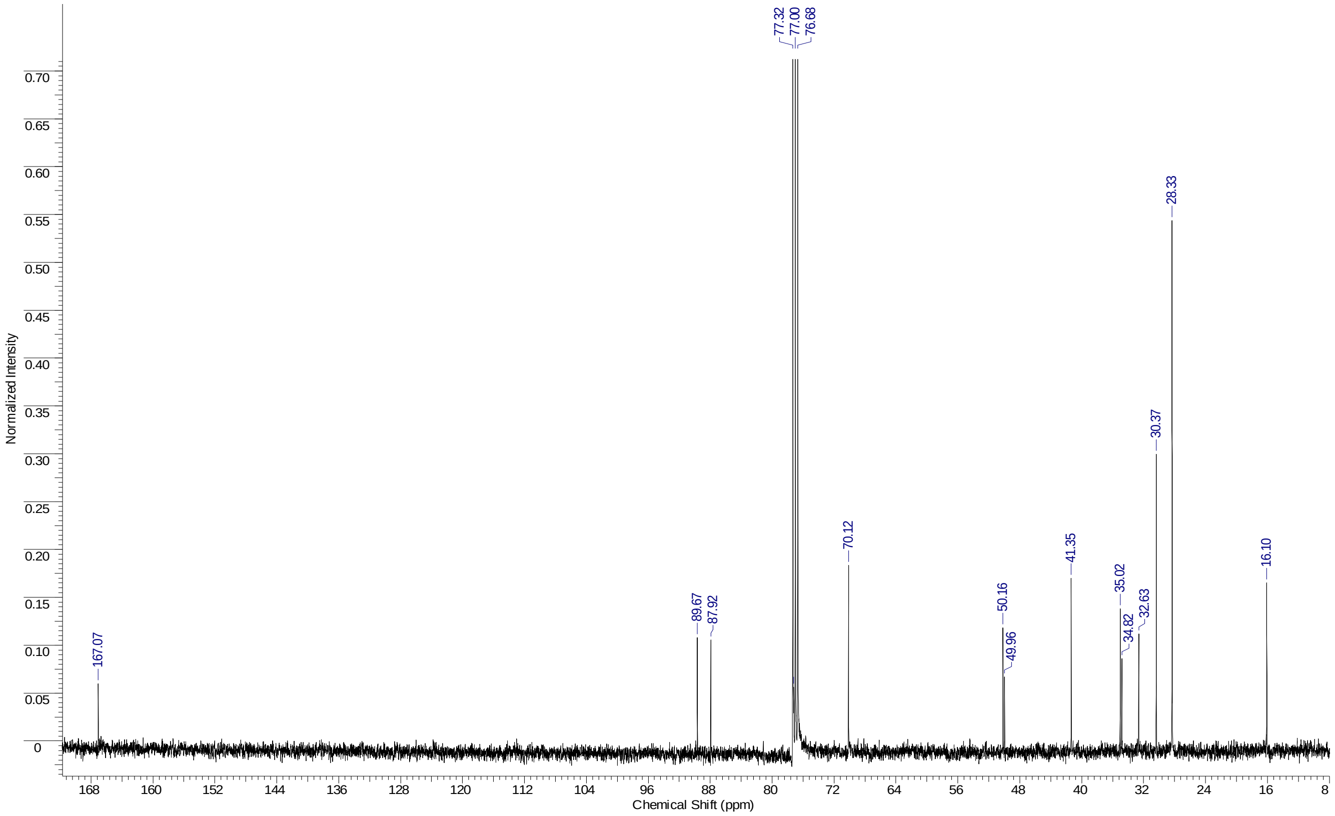


## $^{19}$F NMR (282 MHz)

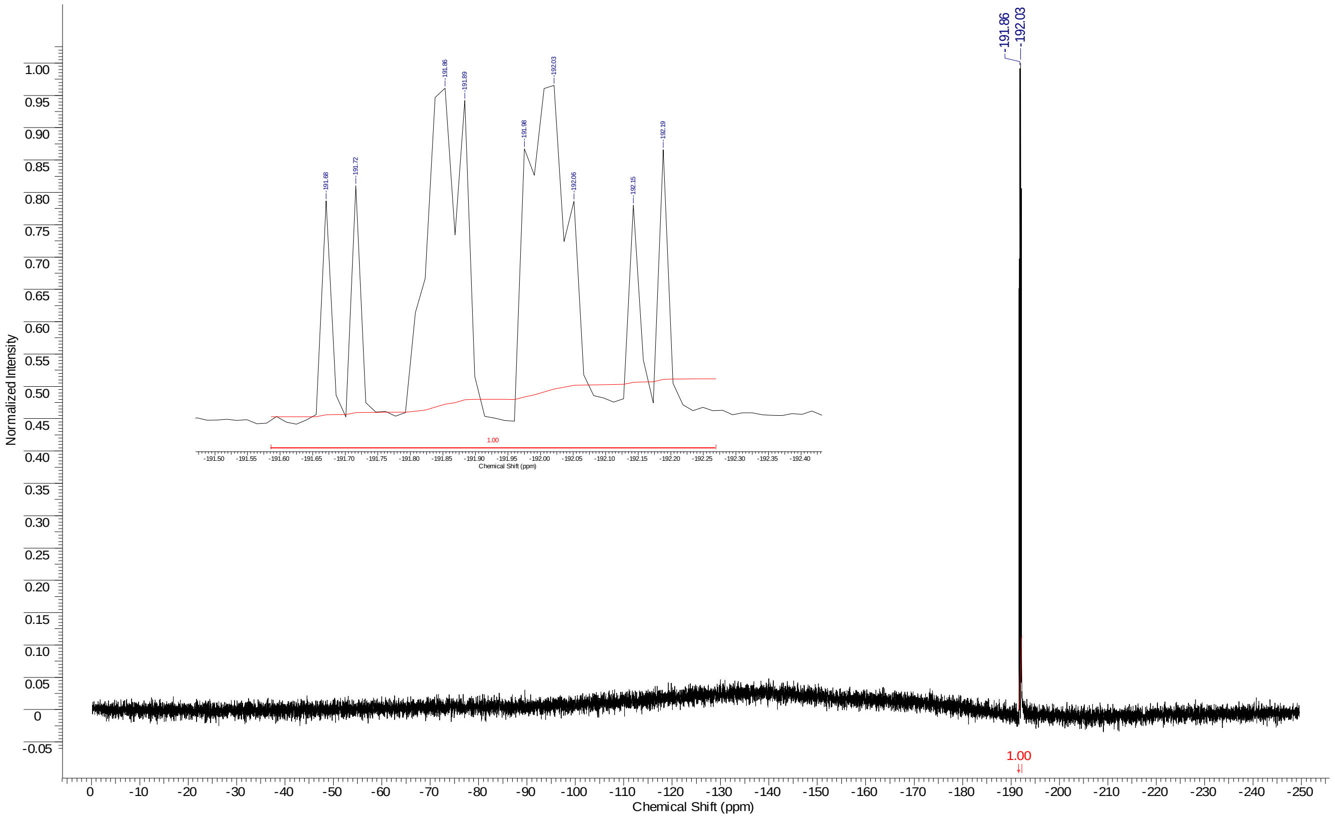

Supplement: Supplementary file 1 [file anie0051-6176-SD1.pdf]
